# Layer-specific changes in sensory cortex across the lifespan in mice and humans

**DOI:** 10.1038/s41593-025-02013-1

**Published:** 2025-08-11

**Authors:** Peng Liu, Juliane Doehler, Julia U. Henschke, Alicia Northall, Angela Knaf-Serian, Laura C. Loaiza-Carvajal, Eike Budinger, Dietrich S. Schwarzkopf, Oliver Speck, Janelle M. P. Pakan, Esther Kuehn

**Affiliations:** 1 Institute for Cognitive Neurology and Dementia Research (IKND), Otto von Guericke University Magdeburg, Magdeburg, Germany; 2 Hertie Institute for Clinical Brain Research (HIH), Tübingen, Germany; 3 German Centre for Neurodegenerative Diseases (DZNE), Magdeburg, Germany; 4 German Centre for Neurodegenerative Diseases (DZNE), Tübingen, Germany; 5 Leibniz Institute for Neurobiology, Magdeburg, Germany; 6 Department of Neuropsychology and Psychological Resilience Research, Research Group Learning and Brain Plasticity in Mental Disorders, Central Institute of Mental Health, Medical Faculty, Mannheim Heidelberg University, Mannheim, Germany; 7 Centre for Behavioural Brain Sciences (CBBS) Magdeburg, Magdeburg, Germany; 8 School of Optometry & Vision Science, The University of Auckland, Auckland, New Zealand; 9 Experimental Psychology, University College London, London, UK; 10 Department of Biomedical Magnetic Resonance, Otto von Guericke University Magdeburg, Magdeburg, Germany

**Keywords:** Sensory processing, Cortex

## Abstract

The segregation of processes into cortical layers is a convergent feature in animal evolution. However, how changes in the cortical layer architecture interact with sensory system function and dysfunction remains unclear. Here we conducted functional and structural layer-specific in vivo 7T magnetic resonance imaging of the primary somatosensory cortex in two cohorts of healthy younger and older adults. Input layer IV is enlarged and more myelinated in older adults and is associated with extended sensory input signals. Age-related cortical thinning is driven by deep layers and accompanied by increased myelination, but there is no clear evidence for reduced inhibition. Calcium imaging and histology in younger and older mice revealed increased sensory-evoked neuronal activity accompanied by increased parvalbumin expression as a potential inhibitory balance, with dynamic changes in layer-specific myelination across age groups. Using multimodal imaging, we demonstrate that middle and deep layers show specific sensitivity to aging across species.

## Abstract (web summary)

The principal layer architecture of the sensory cortex is altered with aging. The authors show that overall thinning of the primary somatosensory cortex is driven by deep layer degeneration but that layer IV is more pronounced in old age.

## Main

Sensory processing is organized in a layered architecture with segregated input, output and modulatory circuits. The layered architecture of sensory systems is a convergent feature in animal evolution^1^. A model of functional and dysfunctional sensory systems requires a detailed understanding of alterations in the layer-specific architecture and their associated phenotypes. Critically, this knowledge is thus far lacking not only for sensory systems but also for cortical dysfunction in general.

Sensory dysfunction has been associated with different cortical phenotypes, including functional overactivation^2^, increases in receptive field sizes^2,3^, decreases in lateral inhibition^2^ and structural alterations such as cortical thinning^4^. However, it is unclear how changes in the cortical layer architecture may contribute to the specific functional and structural alterations that characterize sensory cortices with reduced functionality. Cortical aging serves as a suitable model system to investigate this question as structural and functional plasticity is observed at different levels of the processing hierarchy and affects behavior^2^.

In the present study, we tested four major hypotheses of how structural and functional alterations in the cortical layer architecture characterize cortical dysfunction. The ‘preserved layer hypothesis’ assumes that the structural layer architecture cannot be distinguished between younger and older adults with present methodology, motivated by the preserved layer architecture of primary motor cortex (MI) in older adults^5^. Conversely, the ‘altered input channel hypothesis’ assumes that age-related functional plasticity is characterized by a more (or less) pronounced input layer IV in older compared with younger adults. Previous studies showed structural sensitivity of layer IV to sensory input statistics^6^, which are higher in older adults due to increased age but also weakened due to reduced peripheral nerve receptor density^7^. Our third ‘altered sensory modulation hypothesis’ assumes that older compared with younger adults are characterized by a degradation of deep layers V and VI, which mediate changes in the excitatory–inhibitory modulation of sensory inputs. In primates, deep layers of sensory cortex mediate inhibition^8^, whereas, in older adults, inhibition is reduced^2^. Finally, the ‘degraded border hypothesis’ assumes that low-myelin borders that exist inferior to the thumb representation^9^, and occasionally between single-finger representations^10^, in input layer IV degrade with cortical aging and are associated with less precise functional representations^3^.

**Fig. 1:**
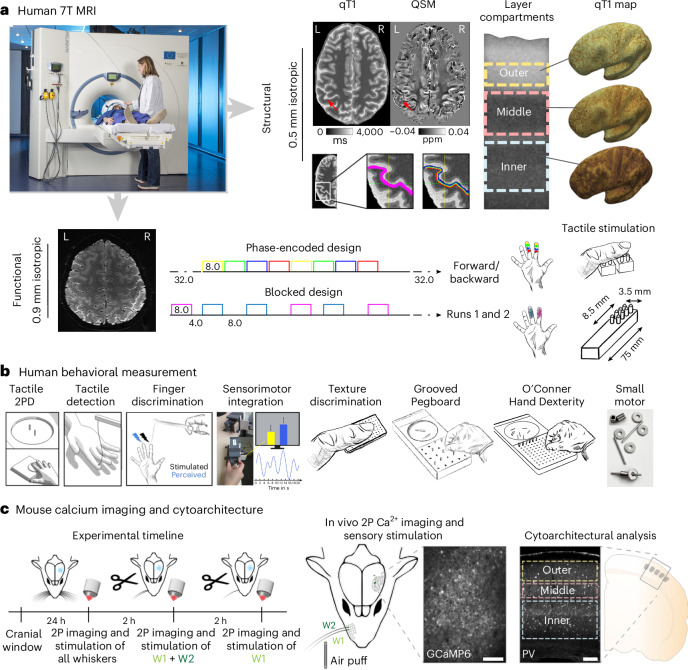
Overview experimental design. **a**, 7T MRI was used to investigate the layer-specific structural architecture of the SI in younger and older adults (red arrows indicate ROI). qT1 values were used to define SI layer compartments in vivo. Layer-specific qT1 and QSM values were mapped onto the individuals’ inflated cortical surfaces. Myelin staining was remodeled according to Dinse et al.^14^. 7T fMRI was used to investigate age-specific and layer-specific functional changes in SI. Participants underwent several passive tactile stimulation paradigms (phase-encoded design and blocked design) in the scanner where fingers of the right hand were stimulated. L, left side of the human brain; R, right side of the human brain. ppm, parts per million. **b**, Younger and older adults underwent a behavioral test battery including tactile two-point discrimination (2PD), tactile detection, tactile finger discrimination, sensorimotor integration and tactile texture discrimination tasks as well as the Grooved Pegboard Test, the O’Connor Hand Dexterity Test and the Small Motor Test. **c**, Barrel cortex two-photon calcium imaging (2PCI) of excitatory neurons expressing a genetically encoded calcium indicator (GCaMP6f) during air puff whisker stimulation was used to investigate younger and older adult mice: (i) during baseline conditions with all whiskers, (ii) after all whiskers were cut except two on the right side (W1 + W2; double stimulation condition), and (iii) after another whisker was cut, leaving only one (W1; single stimulation condition). Schematic indicating region used for postmortem histological analysis examining underlying cytoarchitectural differences across cortical layers and aging. Scale bars, 100 µm. h, hours. Source data

To test these hypotheses, we employed a unique approach and acquired layer-specific functional and structural magnetic resonance imaging (fMRI) data using 7T MRI of the primary somatosensory cortex (SI) of healthy younger and older adults together with behavioral assessments. To better understand the mechanistic underpinnings, we analyzed younger, older and senescent mice using in vivo two-photon calcium imaging and histological analyses (Fig. 1). In humans, analyses were restricted to area 3b—the homolog of mouse SI^11^. This study inspires the precise investigation of cortical layer dynamics to reveal fundamental mechanisms that underlie cortical dysfunction.

## Results

### Age-related cortical thickness changes are layer specific

**Fig. 2:**
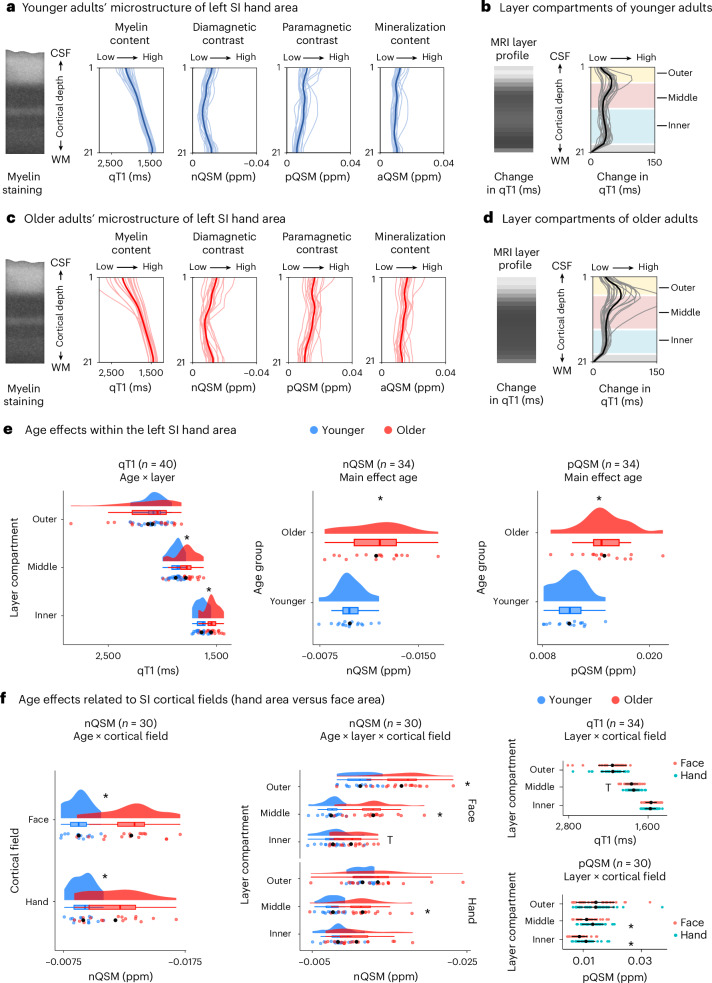
Age-related differences in the structural layer architecture of human SI. **a**,**c**, Younger (**a**) and older (**c**) adults’ microstructure described by qT1-based myelin (*n* = 20 each), nQSM-based diamagnetic contrast (younger: *n* = 18, older: *n* = 16), pQSM-based paramagnetic contrast (younger: *n* = 18, older: *n* = 16) and aQSM-based mineralization (younger: *n* = 18, older: *n* = 16) sampled between the CSF/gray matter border and the gray matter/WM border (group mean plotted in bold). Low qT1 and nQSM indicate high myelin content and high diamagnetic contrast, respectively. Myelin staining was remodeled according to Dinse et al.^14^. QSM values are given in ppm. **b**,**d**, Layer compartments of younger (**b**) and older (**d**) adults based on the local rate of change in qT1 (ref. ^10^). MRI layer profiles visualize the local rate of change in qT1 as shades of gray. **e**, Age-related differences in qT1, nQSM and pQSM in the SI hand area (permutation mixed-effects ANOVAs). *P < 0.0045, significant differences at Bonferroni-corrected significance level. For exact values, see Supplementary Tables 10 and 11. **f**, Age-related differences in SI cortical fields. Shown are permutation mixed-effects ANOVAs with factors age (younger adults, older adults), layer and cortical field (hand area, face area) on residual qT1, nQSM and pQSM after regressing out the effect of map size. *P < 0.002, significant differences at Bonferroni-corrected significance level. Trends above Bonferroni-corrected threshold are marked by ‘T’. For exact values, see Supplementary Tables 8 and 9. For similar data of *n* = 1 participant with congenital arm loss, see Extended Data Fig. 2, Supplementary Fig. 2 and Supplementary Table 5. In **e** and **f**, individual data are shown as colored dots and group means are shown as black dots. Box plots are drawn within the interquartile range (box), medians are shown as vertical lines, and whiskers connect the minimum and the maximum with the lower and upper quartiles. Horizontal black lines represent the lower and upper Gaussian confidence limits. Two-sided tests are reported. CSF, cerebrospinal fluid; WM, white matter. Source data

**Extended Data Fig. 1:**
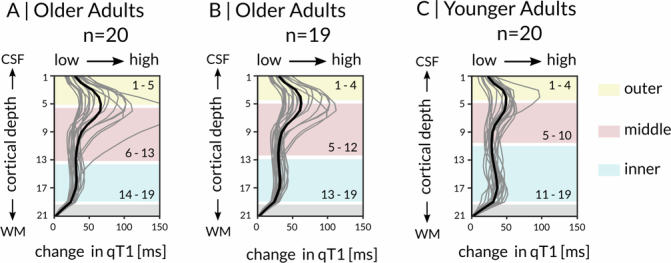
Anatomically-relevant cortical layer compartments. **(a)** Cortical layer compartments included in statistical analyses. The layer definition (that is, minima and maxima of the first derivative of raw qT1 values sampled in 21 depths from superficial to deep; group mean plotted in black, individual data plotted in gray) was based on the left area 3b hand area (identified by vibrotactile stimulation to the five fingertips of the right hand, see more details on layer definition in^10^) using the full sample of older adults (that is n = 20). **(b)** Cortical layer compartments after removing one outlier (participant 40) from the full sample, leaving n = 19 older adults to define the cortical layer compartments. **(c)** Cortical layer compartments of younger adults shown as reference. Source data

Our first hypothesis expects the structural layer architecture to be similar between younger and older adults (‘preserved layer hypothesis’). According to this view, if cortical thinning occurs with aging, we expect homogenous thinning of all layers. To target this fundamental aspect of cortex organization, we analyzed structural and functional 7T MRI data of younger (*n* = 20) and older (*n* = 20) adults’ SI (cohort 1) using the SI hand area as a model system. We localized the SI hand area using tactile population receptive field (pRF) modeling^3^. The SI layer definition followed a data-driven approach, assigning biological layers II and III to the outer compartment, input layer IV to the middle compartment and layers V and VI to the inner compartment^10^. In agreement with previous findings^12^, the SI hand area was, on average, 2.00 ± 0.10 mm thick (mean ± s.d.) but thinner in older compared with younger adults^4^ (older − younger = −0.12 mm; Table 1). Critically, overall cortical thinning is driven by reduced thickness of the inner compartment, whereas the middle compartment is thicker in older compared with younger adults (Table 1 and Fig. 2b,d; see Supplementary Table 2 for cortical thickness analysis without outlier removal; see Supplementary Table 3 for a different localization approach; see Supplementary Table 4 for a different layer compartmentalization scheme; see Extended Data Fig. 1 for the exact definition of cortical layer compartments; and see Supplementary Fig. 3 for the distribution of layer-specific cortical thickness values).

**Table 1:** Layer-specific cortical thickness values of the SI hand area

Hand area	All *n* = 39	Younger *n* = 20	Older *n* = 19	Group differences			
	Mean ± s.d.	Mean ± s.d.	Mean ± s.d.	*t*	*df*	*P*_perm_	CI_perm_
Total	2.00 ± 0.10	2.06 ± 0.07	1.94 ± 0.08	5.0	35.8	<10^−5^*	0.062, 0.183
Outer	0.40 ± 0.02	0.41 ± 0.02	0.40 ± 0.02	1.3	34.7	0.196	−0.004, 0.023
Middle	0.70 ± 0.15	0.56 ± 0.02	0.85 ± 0.03	−34.6	28.3	<10^−5^*	−0.386, −0.198
Inner	0.90 ± 0.21	1.10 ± 0.06	0.69 ± 0.04	26.5	31.3	<10^−5^*	0.275, 0.539

Shown are total and layer-specific (outer, middle, inner) mean cortical thickness values (mean) and standard deviations (s.d.) in millimeters for the SI hand area. Independent-sample random permutation Welch *t*-tests were calculated to investigate group differences (*t*, test statistic; *df*, degrees of freedom; *P*_perm_, Monte Carlo permutation *P* value; CI_perm_, 95% Monte Carlo permutation confidence interval; number of permutations = 100,000; minimum value of *P*_perm_ = 1 / number of permutations). *P < 0.0125, significant Bonferroni-corrected differences (corrected for 4 tests). Two-sided tests are reported. See Supplementary Table 1 for Bayesian independent-sample *t*-tests computed on these differences. Data of *n* = 19 older adults are presented here after outlier removal. The full dataset can be found in Supplementary Table 2.

Source data

Bayesian independent-sample *t*-tests confirm this result, showing extreme evidence for reduced total cortical thickness (BF_10_ = 1,383.79), reduced inner layer thickness (BF_10_ = 2.05 × 10^26^) and increased middle layer thickness (BF_10_ = 8.93 × 10^21^) in older adults compared with younger adults and anecdotal evidence for the null hypothesis of no group difference for the outer layer (BF_10_ = 0.635) (Supplementary Table 1). This result of layer-specific cortical thickness differences between younger and older adults rejects the ‘preserved layer hypothesis’.

**Extended Data Fig. 2:**
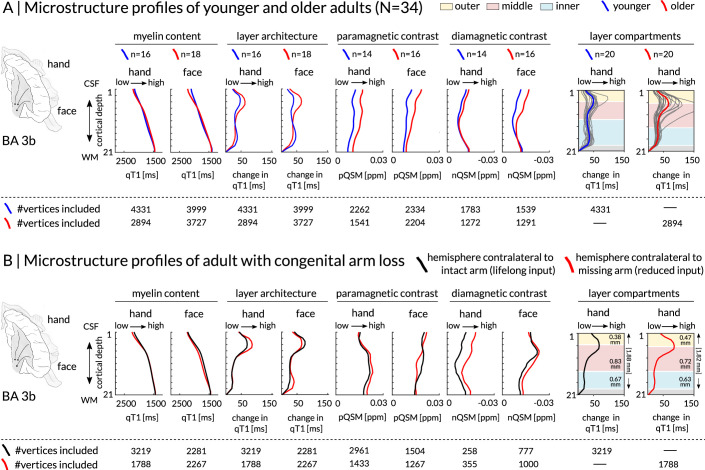
Structural SI Layer Architecture in Younger Adults, Older Adults and with Congenital Arm Loss. **(a)** Microstructural profiles of younger adults and older adults, extracted from the left hemisphere (contralateral to finger and tongue movements). qT1 values are given in milliseconds (ms), nQSM and pQSM values in parts per million (ppm). Three layer compartments were extracted based on localizing maxima and minima of the first derivative of raw qT1 values. For qT1 and nQSM, lower values indicate higher substance concentration. **(b)** Microstructural profiles of a healthy adult (male, age=52 years) with congenital arm loss on the right side, extracted from the hemispheres contralateral and ipsilateral to the missing arm. The cortex of the hand area contralateral to the missing arm (identified via mental imagery of finger movements, see Supplementary Fig. 1) is thinnest (hand contralateral: 1.82 mm; hand ipsilateral: 1.88 mm; face contralateral: 1.96 mm; face ipsilateral: 1.93 mm). Layer-specific cortical thickness extraction reveals a thinner middle compartment for the hand area contralateral compared to ipsilateral to the missing limb (hand contralateral: outer=0.47 mm, middle=0.72 mm, inner=0.63 mm; hand ipsilateral: outer=0.38 mm, middle=0.83 mm, inner=0.67 mm). Note that layer-specific thickness values of the adult with congenital arm loss are in a plausible data range (taking the range of two-handed participants for the hand area as reference: outer=0.38–0.58 mm, middle=0.53–0.83 mm, inner=0.60–1.17 mm). Source data

We also examined a healthy adult (male, age 52 years) with congenital arm loss on the right side to further investigate variations in layer-specific cortical thickness in the area 3b hand area (see Supplementary Fig. 1 for functional localizer). Detailed results are shown in Extended Data Fig. 2 and Supplementary Fig. 2.

### Stable low-myelin borders in aging and in a one-handed person

Next, we tested the ‘degraded border hypothesis’. This hypothesis assumes that the low-myelin borders that exist inferior to the thumb representation^9^, and occasionally between single-finger representations^10^, in input layer IV of SI degenerate with age. We first confirmed that the overall structural topographic architecture of SI follows the expected pattern—that is, individual-finger representations do not significantly differ in their microstructural composition (Supplementary Tables 6 and 7), whereas the hand and the face areas show microstructural differences, indicating distinct cortical fields^10^. In particular, we show more pronounced diamagnetic contrast in the face area in older adults (Fig. 2f and Supplementary Tables 8 and 9).

**Fig. 3:**
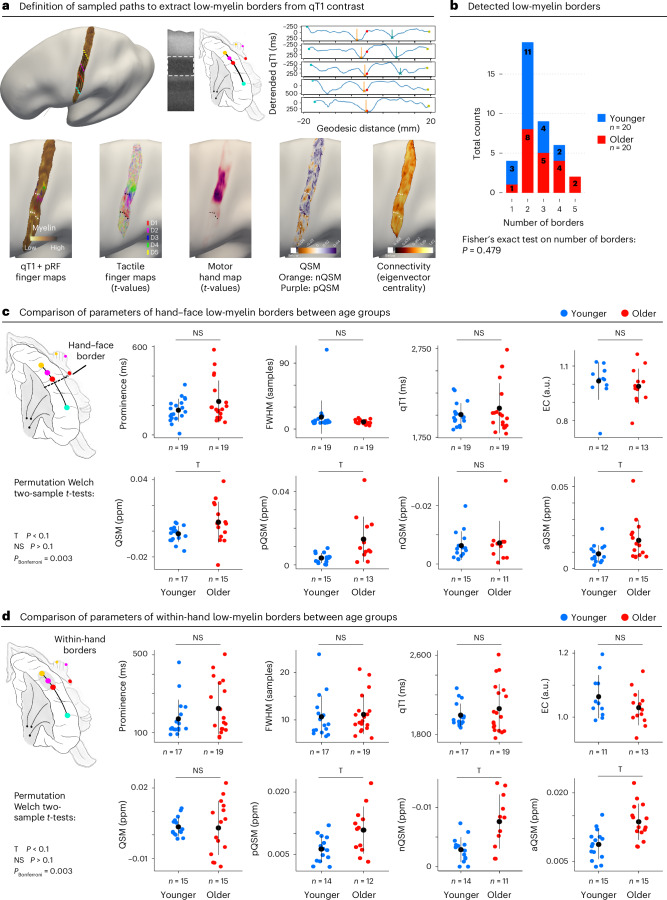
Structural border results using an automated detection approach. **a**, Low-myelin borders (vertical lines) were detected based on detrended qT1 values sampled along multiple geodesic paths from the face representation to the little finger representation of the hand. The analysis was based on qT1 values extracted from the middle layer compartment (dotted white line in myelin stain (remodeled according to Dinse et al.^14^)). Additional seeds were placed at the thumb representation (anchor to calculate geodesic distances) and the index finger representation. Geodesic paths were sampled along the inferior-to-superior axis. Five paths were extracted from anterior to posterior within area 3b using equal spaces between neighboring paths. Detected low-myelin borders were back-projected to cortical surfaces (white dots in enlarged surface plots) and are shown together with different contrasts (from left to right): middle qT1 together with pRF center location maps of individual fingers, finger activation maps (*t*-values) extracted from vibro-tactile blocked design paradigm, hand activation map (*t*-values) extracted from motor blocked design paradigm (that is, individual finger movements), QSM indicative of diamagnetic (nQSM) and paramagnetic (pQSM) areas and connectivity map (eigenvector centrality (EC) values). **b**, Total counts of detected low-myelin borders for younger and older adults. Fisher’s exact test indicated no difference in the number of detected borders between age groups. **c**, Comparison of hand–face low-myelin border composition between age groups. No significant differences were observed between age groups with respect to border prominence, full width at half maximum (FWHM), qT1 intensity, EC, signed QSM values (QSM), pQSM values, nQSM values or aQSM values. For *n* = 2 participants, no hand–face border was detected. **d**, Comparison of within-hand low-myelin border composition between age groups. No significant differences were observed between age groups. Trends above Bonferroni-corrected threshold of *P* = 0.003 (correcting for 16 tests) are marked by ‘T’. NS, not significant. Source data

To test our hypothesis, we developed an automatic border detection algorithm (Fig. 3a), which did not detect significant age-related differences with respect to the existence or composition of layer IV low-myelin borders between younger and older adults (Fig. 3b–d). Statistical trends that indicate differences in iron and calcium content in the hand–face border between age groups (Fig. 3c,d) are not specific to the border area but are generally observed in SI.

The same automatic border detection algorithm was applied to the data acquired from *n* = 1 congenital one-handed person, revealing that low-myelin borders exist in both hemispheres of this individual—that is, contralateral and ipsilateral to the missing arm (Supplementary Fig. 5). Low-myelin borders, therefore, have a similar architecture in younger adults, older adults and in *n* = 1 individual with congenital arm loss. This rejects the ‘degraded border hypothesis’, confirming a previous observation in human MI^5^.

### More pronounced layer IV input signals in older adults

**Fig. 4:**
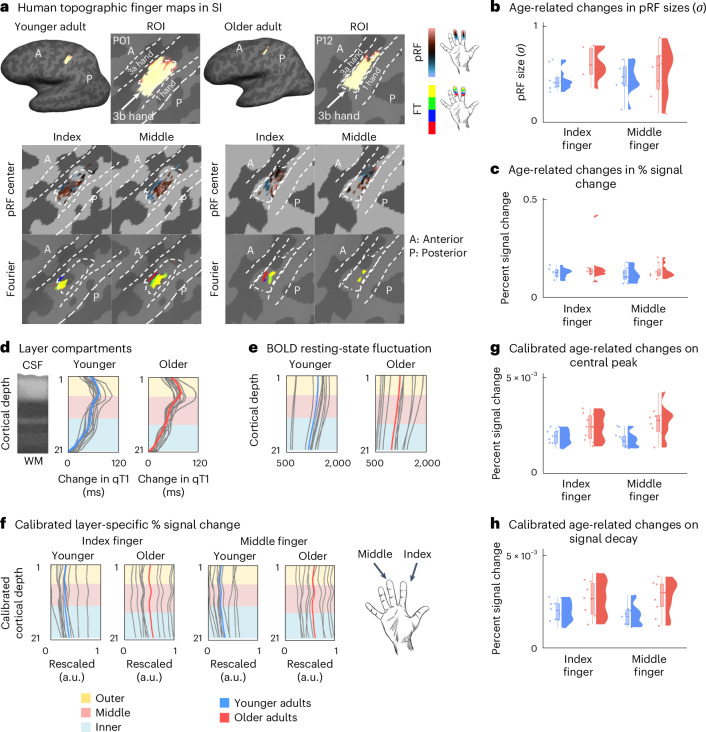
Layer-specific functional architecture of human SI. **a**, Topographic representations of the index and middle fingers shown for one younger adult (P01) and one older adult (P12) (randomly chosen; see Supplementary Fig. 6 for all individuals). Topographic maps are based on pRF modeling (first row) and Fourier-based analyses (second row). ROI (hand area of contralateral area 3b). FT, Fourier transform. **b**, pRF size (*σ*) estimates of index and middle finger representations (a.u.); individual data shown as colored dots: younger adults (*n* = 11), older adults (*n* = 10). Box plots are drawn within the interquartile range (box), medians are shown as vertical lines, and whiskers connect the minimum and the maximum with the lower and upper quartiles. **c**, Percent signal change of index and middle finger representations; individual data shown as colored dots: younger adults (*n* = 11), older adults (*n* = 10). Box plots are drawn within the interquartile range (box), medians are shown as vertical lines, and whiskers connect the minimum and the maximum with the lower and upper quartiles. **d**, Layer compartments for younger (*n* = 11, blue) and older (*n* = 10, red) adults defined based on rate of change in qT1 (ref. ^10^). **e**, Resting-state signal fluctuation extracted at different cortical depths for younger (*n* = 11) and older (*n* = 10) adults. **f**, Calibrated percent signal change (scaled between 0 and 1) of index and middle finger representations extracted at different cortical depths (see Supplementary Fig. 7b for data without calibration). **g**, Central peak of index and middle finger representations; individual data shown as colored dots: younger adults (*n* = 11), older adults (*n* = 10). Box plots are drawn within the interquartile range (box), medians are shown as vertical lines, and whiskers connect the minimum and the maximum with the lower and upper quartiles. **h**, Signal decay of index and middle finger representations; individual data shown as colored dots: younger adults (*n* = 11), older adults (*n* = 10). Box plots are drawn within the interquartile range (box), medians are shown as vertical lines, and whiskers connect the minimum and the maximum with the lower and upper quartiles. Source data

The above-reported results reject both the ‘preserved layer hypothesis’ and the ‘degraded border hypothesis’. However, the increased thickness of input layer IV in older adults is in line with the ‘altered input channel hypothesis’. To follow this up, we investigated, in an independent cohort (cohort 2, *n* = 11 younger adults, *n* = 10 older adults), if there is evidence for extended sensory input signals in layer IV in older compared with younger adults. We extracted both resting-state and tactile stimulation-induced percent signal change of index and middle finger representations (Fig. 4; see Supplementary Figs. 6 and 7 for data of all participants) in contralateral SI for each individual at each modeled cortical depth. In both younger and older adults, there is an antagonistic center-surround relationship between signals and cortical depth: signals peaked in the input layer IV but were minimal in neighboring depths. Given that we observed this relationship only during sensory stimulation but not during the resting state (Fig. 4e versus Fig. 4f), and the peak occurred at the expected layer compartment encompassing layer IV, the signal peak is a marker to measure input signals to layer IV in SI.

Older adults present with a more pronounced sensory input peak compared with younger adults (Fig. 4f–h; for detailed statistics, see Supplementary Table 14; note that analyses were performed on calibrated blood oxygen level dependent (BOLD) data, reducing the likelihood that effects are driven by age-related differences in neurovascular coupling). This provides further evidence for the ‘altered input channel hypothesis’, according to which the cortical input circuit changes with aging, although reduced inhibition could also explain this effect (‘altered modulation hypothesis’).

### Altered functional response profile in the SI of older adults

Thus far, we have rejected both the ‘preserved layer hypothesis’ and the ‘degraded border hypothesis’ and have provided evidence for the ‘altered input channel hypothesis’. With respect to the third ‘altered modulation hypothesis’, we hypothesized that deep layer degeneration (evidenced above by age-related deep layer thinning) is related to coarser functional selectivity and/or reduced co-activated inhibition in older adults^2^. To test for functional selectivity, we computed the sharpness of index and middle finger pRFs (sigma parameter (*σ*)). To test for co-activated inhibition, we compared mean percent signal change and mean pRF sizes between a ‘double stimulation condition’ and a ‘single stimulation condition’^13^. Note that the double versus single stimulation comparison implies comparing single finger versus double finger stimulation in humans and single versus double whisker stimulation in mice (for the latter, see the ‘Altered neuronal activity and layer architecture in mice’ subsection).

Results show strong evidence for coarser functional selectivity of the index finger representation in older compared with younger adults, whereas the middle finger shows anecdotal evidence favoring the null hypothesis (for detailed statistics, see Extended Data Table 1). Against our expectation, statistical evidence for age differences in markers of co-activated inhibition is inconclusive (for detailed statistics, see Extended Data Table 2). This suggests that middle layer expansion and deep layer thinning co-occur with coarser spatial selectivity of the index finger representation, but we cannot confirm that this co-occurs with decreased inhibition in older adults.

### Altered microstructural layer composition in older adults

Next, we investigated how the microstructural composition of SI layers changes with age. We extracted quantitative T1 (qT1) values as a proxy for cortical myelin^14^ and quantitative susceptibility maps (QSMs) as proxies for cortical iron (positive values, pQSM) and calcium/metabolism (negative values, nQSM)^15^, as well as overall mineralization (absolute values, aQSM)^16^, from different depths of the human hand area. We confirm previously reported across-layer age effects^5,16^: (1) more negative nQSM values (older: −0.0118 ± 0.0006 parts per million (ppm); younger: −0.0098 ± 0.0003 ppm), (2) higher pQSM values (older: 0.0149 ± 0.007 ppm; younger: 0.0110 ± 0.0004 ppm) and (3) higher aQSM values (older: 0.0138 ± 0.0006 ppm; younger: 0.0103 ± 0.0003 ppm) in older compared with younger adults (Supplementary Tables 10 and 11).

Interestingly, age effects in qT1 values are layer specific, with lower qT1 values (reflecting higher myelin) in the inner and middle compartments in older compared with younger adults (older: inner = 1,547.6 ± 14.8 ms (mean ± s.e.); middle = 1,783.2 ± 21.9 ms; younger: inner = 1,636.3 ± 12.3 ms; middle = 1,874.4 ± 12.9 ms; Fig. 2e; see Supplementary Tables 10 and 11 for exact results; see Supplementary Tables 12 and 13 for control analyses using a different compartmentalization scheme). Controlling for effects of cortical atrophy, no significant difference was observed in hand mask (full hand map) size (qT1 (*n* = 40): *t*_33.7_ = −0.48, *P* = 0.632; nQSM (*n* = 34): *t*_31.8_ = 0.67, *P* = 0.510; pQSM (*n* = 34): *t*_30.1_ = −0.39, *P* = 0.696; aQSM (*n* = 34): *t*_29.4_ = 0.02, *P* = 0.981) between younger adults (qT1: 1,249 ± 90; nQSM: 512 ± 53; pQSM: 717 ± 62; aQSM: 1271 ± 98 vertices, mean ± s.e.) and older adults (qT1: 1,325 ± 130; nQSM: 462 ± 53; pQSM: 756 ± 75; aQSM: 1,268 ± 124 vertices).

These results are in line with the ‘altered input channel hypothesis’: the input layer IV does not only appear thicker and shows more pronounced sensory input signals in older compared with younger adults, but it is also characterized by higher myelin content. These results are also in line with the ‘altered modulation hypothesis’: the deeper layers of SI are thinner in older adults, and the functional spatial selectivity of the index finger representation is coarser, but this is not accompanied by evidence for reduced functional inhibition. The reason may be the higher myelin content in deeper layers in older adults. To clarify the role of age-related alterations in layer-specific cellular composition more precisely, additional analyses were conducted in younger and older mice.

### Altered neuronal activity and layer architecture in mice

Although BOLD responses are difficult to relate to precise neural excitatory or inhibitory drive, and MRI data are difficult to relate to precise histological changes, the use of animal models can help clarify mechanistic aspects related to the cytoarchitectural changes observed in specific layers and associated functional changes. We examined the aging whisker barrel cortex in mice as an analogous sensory system that shares multiple topological features and aspects of cortical representation with the human hand and fingers^11^. We investigated the activity of individual neurons in the equivalent of younger adults (mice 19.6 ± 2.5 weeks, *n* = 8—that is, between 2 months and 6 months) and older adults (mice 76.7 ± 2.2 weeks, *n* = 8—that is, between 12 months and 20 months) and combined this with postmortem histological analysis across cortical layers and age.

**Fig. 5:**
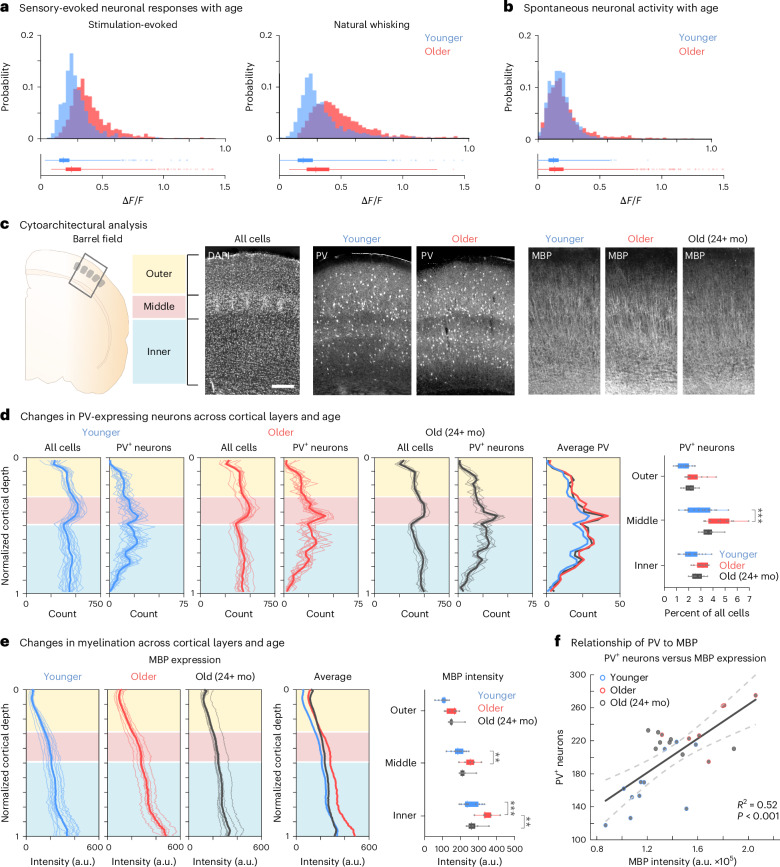
Age-related functional and structural changes in mouse barrel cortex. **a**,**b**, Average change in fluorescence (Δ*F*/*F*) for all neurons across younger (*n* = 1,519 neurons) and older (*n* = 1,958 neurons) adult mice (from eight mice for each age group) during sensory-evoked neuronal activity after air puff stimulation (left) and during periods of natural whisking (right) (**a**) and spontaneous neuronal activity during periods with no whisker pad movement (**b**). **c**, Schematic of the mouse barrel field where images of DAPI staining of all cells and PV for younger adult mice (*n* = 17, 2–6 months), older adult mice (*n* = 13, 12–20 months) and mice in old age (*n* = 8, +24 months) and MBP immunohistochemistry from the barrel cortex were collected for analysis in a subset of these mice (younger adult mice (*n* = 11, 2–6 months), older adult mice (*n* = 7, 12–20 months) and mice in old age (*n* = 8, +24 months)) and across cortical layers (inner, layer II/III; middle, layer IV; outer, layer V/VI). Scale bar, 100 µm. **d**,**e**, Quantification of the binned sum of PV-expressing cells (**d**) and the intensity of MBP staining (**e**) is shown across the cortical depth, normalized from the brain’s surface (0) to the white matter (1); shaded lines, individual mice; thicker lines, average across mice for each age group. The percentage of PV-expressing cells (**d**, right, normalized to the DAPI count per section) and the mean intensity of MBP staining (**e**, right) were quantified as the average across cortical layers and age groups per mouse. **f**, Linear regression *R*^2^ and 95% confidence intervals (dashed lines) for the intensity of MBP expression (average intensity per sample, summed across cortical depth) and number of PV^+^ cells (average count per sample, summed across cortical depth) per animal (*R*^2^ = 0.52, *P* < 0.001, *n* = 26 mice, younger adult mice (*n* = 11, 2–6 months), older adult mice (*n* = 7, 12–20 months) and mice in old age (*n* = 8, +24 months)). Asterisk indicates significance level of **P < 0.01 and ***P < 0.001; in **d** and **e**, values from Tukey–Kramer corrections. Box plots are drawn within the interquartile range (box), medians are shown as vertical lines, and whiskers extend from the box to data points within a 5× interquartile range (**a**,**b**) or most extreme data points (**d**,**e**). mo, months. Source data

**Extended Data Fig. 3:**
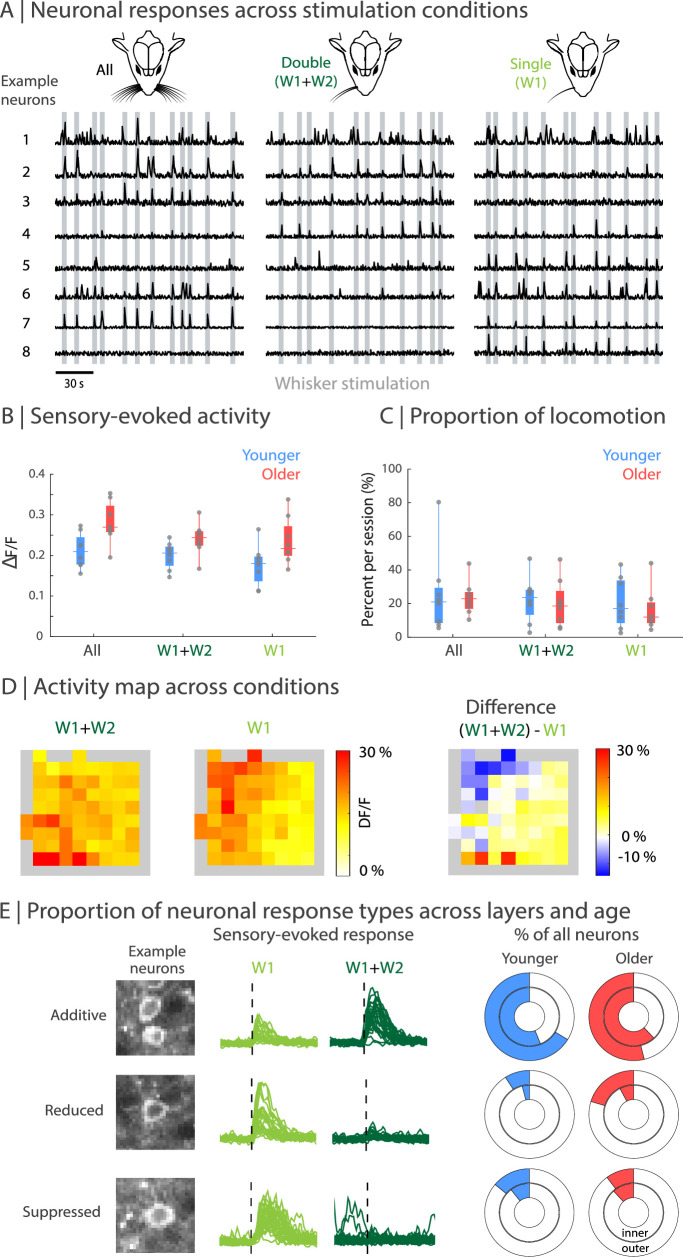
Age-related functional changes in mouse barrel cortex following single whisker and double whisker coactivation. **(a)** Calcium activity from example neurons (1-8) for three stimulation conditions, all whiskers (All), two neighboring whiskers (W1 + W2; double stimulation condition), and single whisker (W1; single stimulation condition). **(b)** Average ΔF/F responses across all neurons within a field-of-view for younger adult mice (1519 neurons from n = 8 mice) and older adult mice (1958 neurons from n = 8 mice) during sensory-evoked airpuff stimulation across conditions. Significant effect of age (F_(2,42)_ = 18.18, *p* < 0.001), with older adult mice showing larger sensory-evoked excitatory neuronal responses (effect of stimulation condition, F_(2,42)_ = 3.22, *p* = 0.050; no significant interaction, F_(2,42)_ = 0.38, *p* = 0.689; two-way mixed-effects ANOVA, constrained (type III) sum of squares; Tukey-Kramer correction). **(c)** Percentage of time that mice spent running during experimental sessions did not differ significantly across age (*p* = 0.525) or stimulation condition (*p* = 0.556; with no interaction (*p* = 0.978) two-way mixed-effects ANOVA, constrained (type III) sum of squares), indicating that locomotion alone is unlikely to contribute to the differences in sensory-evoked activity observed across age (see Fig. 5a). **(d)** Sensory-evoked ΔF/F for an example field-of-view, averaged across 50 × 50 pixel spatial bins during double whisker (left) and single whisker (middle) stimulation and the difference of these two conditions (right). **(e)** Example neurons and sensory-evoked ΔF/F that show additive, reduced, or suppressed responses to single (W1) or double (W1 + W2) whisker stimulation; percentage of all neurons (younger adult mice [total 1519 neurons from n = 8 mice; 1081 outer layer neurons, 438 inner layer neurons] and older adult mice [total 1958 neurons from n = 8 mice; 1446 outer layer neurons, 512 inner layer neurons]) showing these response types across outer (layer II/III, outer ring) and inner (layer V, inner ring) cortical layers. **(b, c)** Box plots drawn within the interquartile range (box), medians shown as vertical lines, whiskers connect the minimum and the maximum with the lower and the upper quartiles. Source data

To examine sensory-driven neuronal activity in younger compared with older adult mice, a cranial window was implanted above the barrel cortex in transgenic mice expressing a genetically encoded calcium indicator (CGaMP6f)^17^ driven by a Thy1 promoter^18^. We examined sensory-evoked responses of excitatory neurons across the outer (layer II/III) and inner (layer V) cortical layers after spontaneous activity and direct air puff stimulation to all whiskers as well as subsets of whiskers (Fig. 1c—that is, similar stimulation paradigm to humans: ‘single stimulation condition’, one whisker (W1); ‘double stimulation condition’, two neighboring whiskers co-activated (W1 + W2); Extended Data Fig. 3). Changes in fluorescence (Δ*F*/*F*) of the calcium indicator were extracted and used as a proxy readout of neuronal activity. We observed a more pronounced increased amplitude of excitatory sensory-evoked responses in older adult mice during direct whisker stimulation of all whiskers as well as during voluntary whisking compared with spontaneous neuronal responses during rest (Fig. 5a,b and Extended Data Fig. 3; younger adult (*n* = 1,519 neurons) versus older adult (*n* = 1,958 neurons): air puff whisker stimulation: *t*_3,475_ = 14.6, *P* < 0.001, Cohen’s *d* = 0.498; natural voluntary whisking: *t*_3,475_ = 16.9, *P* < 0.001, Cohen’s *d* = 0.577; spontaneous neuronal responses: *t*_3,475_ = 10.7, *P* < 0.001, Cohen’s *d* = 0.367; independent-sample *t*-test, data from eight mice per age group; see also Extended Data Fig. 3b). This indicates more pronounced sensory input signals in older adult mice, analogous to that observed in humans (Fig. 4f,g).

The human data on co-activated functional inhibition are inconclusive, with no clear evidence for the expected reduced co-activated inhibition in older adults. In mice, we found suppressive influences of the co-activated double stimulation condition in comparison to the single stimulation condition in spatially averaged activity maps (Extended Data Fig. 3b), although it should be noted that barrel fields were stereotaxically targeted to include the preserved whiskers without precise receptive field mapping (Methods). We observed changes in neuronal responses to whisker stimulation conditions on the single-cell level and in the percentage of responsive neurons across age and cortical layers (Extended Data Fig. 3). Particularly in the outer cortical layers (layer II/III), younger adult mice showed an increased proportion of individual neurons with additive responses (younger: 65%, older: 51%), whereas older adult mice showed a higher proportion of neurons with reduced sensory-evoked responses (younger: 13%, older: 23%) in the double stimulation condition compared with the single stimulation condition, indicating increased inhibitory influence of the neighboring whisker in older adult mice. The more pronounced sensory input signals in older mice as well as increased functional inhibition of neighboring whiskers may help to interpret the human data mechanistically (Fig. 4).

Next, we examined cytoarchitectural changes across layers in the barrel cortex of younger adult (14.8 ± 1.7 weeks, *n* = 17, between 2 months and 6 months) and older adult (68.7 ± 3.8 weeks, *n* = 13, between 12 months and 20 months) mice as well as mice into further old age (114.7 ± 1.1 weeks, *n* = 8, >24 months) (Fig. 5c–e). To clarify the hypothesis that lower qT1 values (indicating higher myelination) in deep layers of older adults could prevent the decrease of co-activated functional inhibition (that is, ‘altered modulation hypothesis’) and to assess changes specific to inhibitory drive, we examined the proportion of neurons expressing parvalbumin (PV^+^) in these mice, which is a calcium binding protein and molecular marker of the largest class of inhibitory interneurons in the cortex^19^. We found a significant effect of age driven by increased PV^+^ density in older mice (*F*_2,105_ = 21.7, *P* < 0.001) and layer (*F*_2,105_ = 39.5, *P* < 0.001) but no interaction (*F*_4,105_ = 1.48, *P* = 0.214) (two-way mixed-effects ANOVA).

To assess the neurophysiological basis for the finding in humans that cortical aging is associated with lower qT1 values (indicating higher myelination) in middle and deep SI layers, we also examined the intensity of myelin basic protein (MBP) immunohistochemistry staining across cortical layers and age in a subset of these mice (younger adult mice (*n* = 11, 2–6 months), older adult mice (*n* = 7, 12–20 months) and mice in old age (*n* = 8, +24 months)). We found that older mice showed increased MBP expression in layer IV and deeper cortical layers; however, in very old age, these cortical layers showed a loss of MBP expression (Fig. 5e; significant effect of age (*F*_2,69_ = 24.0, *P* < 0.001) and layer (*F*_2,69_ = 110.6, *P* < 0.001) but no interaction (*F*_4,69_ = 2.23, *P* = 0.075) (two-way mixed-effects ANOVA)). These results are consistent with previous studies that showed an inverted U-shape relationship between myelination and age^20^. We also found a positive correlation between MBP expression and PV^+^ density across mice (*R*^2^ = 0.52, *P* < 0.001, *n* = 26 mice; Fig. 5f).

Finally, the dynamics of microglia are also known to change with age, and it has been shown that myelin debris accumulates within microglia with aging^20,21^. We found significant differences in microglia density (as measured by Iba1 expression) across cortical layers in this cohort of younger adult mice but not in older ages (with an increased microglial density in upper cortical layers in younger adults that was absent in older adults) (Supplementary Fig. 8). However, we found no significant correlation between microglia and MBP expression (*R*^2^ = 0.09, *P* = 0.120) or PV^+^ neuron density (*R*^2^ = 0.05, *P* = 0.278) across animals (Supplementary Fig. 8), suggesting that the specific functional changes that we observed across aging and cortical layers are more likely linked to changes in excitatory–inhibitory balance and sensory-related altered modulation rather than specific neuroinflammatory dynamics.

### Layer-specific changes and human sensorimotor impairments

**Fig. 6:**
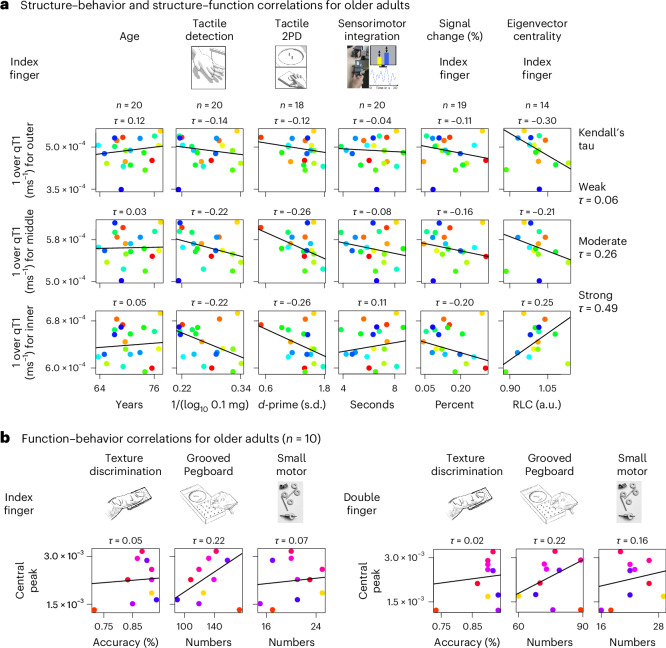
Relation between layer-specific changes and sensorimotor impairments in older adults. **a**, Structure–function and structure–behavior correlations shown for the index finger representation only. Regression lines were generated based on robust Theil–Sen regression estimates^43,44^. Correlation coefficients are given as Kendall’s tau. Cutoffs for the interpretation of Kendall’s tau were approximated by applying the formula *τ* = 2 / π arcsin(*r*) to cutoffs for the Pearson correlation coefficient (*r*)^45,46^. To facilitate the interpretation of the correlations, we ensured that higher values indicate better performance or more myelin; hence, tactile detection and qT1 values (given in milliseconds) were reversed. Network centrality is given as rectified linear unit correlation (RLC), and tactile 2PD is given as *d*-prime. No significant correlations were observed (uncorrected *P* > 0.1, two-sided tests). **b**, Function–behavior correlations shown for index finger and index–middle finger pair. Correlation coefficients are given as Kendall’s tau. No significant correlations were observed (uncorrected *P* > 0.1, two-sided tests). Source data

Finally, we investigated whether interindividual variation in layer-specific microstructure and function is related to age-specific functional and behavioral decline in humans. We confirmed that older adults compared with younger adults show worse tactile and motor performance (for exact results, see Extended Data Tables 3 and 4). We then calculated Kendall’s tau correlations for older adults (Fig. 6), where the following correlations show a moderate relationship: (1) lower qT1 values (higher myelin content) in the superficial layer compartment are associated with lower network centrality of the index finger (*τ* = −0.30) and (2) lower qT1 values (higher myelin content) in middle and deep layer compartments of the index finger representation are associated with worse tactile 2PD performance of the index finger (*τ* = −0.26). Please note, however, that these correlations do not exceed the significance level (*P* > 0.1).

## Discussion

**Fig. 7:**
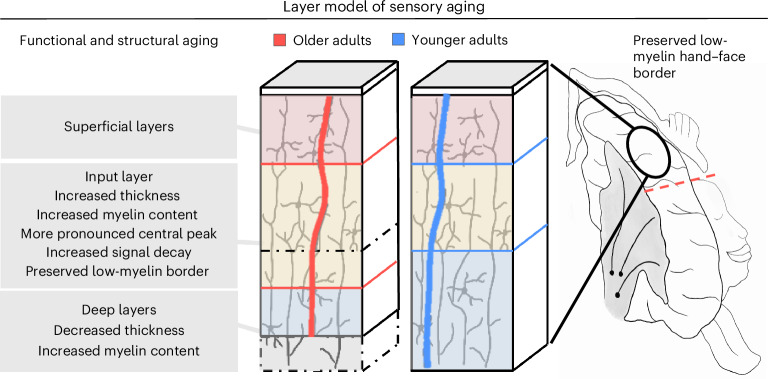
Layer model of sensory aging. The layer model of sensory aging suggests that cortical aging, as one example of cortical dysfunction, has a unique layer-specific profile. Critical characteristics are a widened input channel, a layer-specific profile of cortical thinning driven by deeper layer degeneration (note that, for superficial layers, there is only anecdotal evidence for the null hypothesis of no group difference, BF_10_ = 0.635) and altered modulatory influences on functional representations. Black dotted lines indicate changes in layer-specific thickness in older adults compared with younger adults. Red and blue vertical lines schematically represent layer-specific functional activation for older and younger adults, respectively (with lower to higher activation from left to right—that is, along the *x* axis).

To develop a detailed understanding of alterations in the layer-specific architecture and their associated phenotypes, we studied the layer-specific structural and functional architecture of SI using in vivo ultra-high-field 7T MRI data of two cohorts of healthy younger and older adults. We also investigated SI whisker representations in younger and older mice using in vivo two-photon calcium imaging in combination with histology to derive more information on potential underlying neuronal mechanisms. Altogether, our data reject the idea that the principal layer architecture of the sensory cortex is preserved with aging. Rather, cortical thinning is driven by deeper layers, and the middle compartment, encompassing layer IV, appears thicker in older compared with younger adults. Whereas a previous study uncovered that MI shows vulnerability in the output layer V and superficial layers to aging^5^, in SI, age-related changes are most pronounced in input and deep modulatory layers. These insights (summarized in Fig. 7) suggest that layer-specific degeneration is dependent on the specific microstructural architecture of the cortex and needs to be described separately for different cortical areas.

Older adults present with a thicker and more myelinated input layer that exhibits a more pronounced sensory signal peak. Older mice also show increased MBP expression in layer IV; however, in more extreme old age, this cortical layer shows a loss of MBP expression, which is congruent with previous findings of changes in myelination across aging in animal models^20^ and cross-species studies^22^ as well as in relation to sensory activation^6,23^. A combination of age-driven and experience-driven mechanisms influencing the composition of layer IV is a potential explanation for these findings^6^. The more pronounced layer IV in older age may reflect plasticity over the individual lifespan, followed by degeneration in very old age. Layer IV structural alterations may allow individuals to compensate for peripheral signal loss, by shifting short-range connections toward inhibition to sharpen sensory signals and/or stabilize established functional networks^24,25^. This view confirms the ‘altered input channel hypothesis’ specifying a wider input channel in SI of older adults. An alternative view is that separate mechanisms underlie a thicker layer IV in older adults and potential experience-driven changes of layer IV architecture. Possible contributing mechanisms could be changes in synaptic plasticity^26^ and/or architectural differences of oligodendrocytes, microglia and other neuroglial dynamics with aging^21,27,28^.

Second, we show that age-related cortical thinning in humans is layer specific. Therefore, the usual across-layer estimate of cortical thickness, often used to describe the human cortex in health and disease, may not be as informative as previously assumed. This is true, in particular, given that the middle compartment of SI is thicker despite overall cortical thinning in older adults. Layer-specific computations of cortical thickness, therefore, derive mechanistic information regarding the architecture of the cortex, such as plasticity-related expansion and age-related degeneration. Calculating the average across layers disregards this critical intracortical information.

Deeper layers are thinner but more myelinated in older adults. At the same time, the spatial selectivity of the index finger representation is coarser while there is no clear evidence that co-activated functional inhibition is reduced in older adults. These measures may be connected given that deeper layers in SI are involved in subcortical signal integration, signal modulation and inhibition^29,30^. Whereas the BOLD effect has limitations with respect to its interpretation as excitatory or inhibitory^29^, we replicated sensory-driven overactivation and found increased functional inhibition in older mice using single-neuron activity. In addition, we found higher PV^+^ cell density in older mice. Previous work in mice showed that a large fraction of myelin in cortical layers II/III and IV ensheaths axons of GABAergic interneurons, particularly of PV^+^ basket cells in SI^30^. In humans, PV^+^ interneurons contribute substantially to the total myelin content in the cerebral cortex^31^. One explanation is to assume that the decreased qT1 signal reflects changes in myelination of cortical output with axons oriented through the deeper layers to enter long-range white matter tracts as well as an increase in PV^+^ density. Substantial evidence, and results from the current study in mice, show that cortical myelination continues throughout adulthood and does not begin to decline until well into old age^20,22,32^. This increase in myelination may also be linked to an increase in sensory-evoked activation and subsequent PV^+^ cell density, which may act as compensatory mechanisms for maintaining excitatory–inhibitory balance^33,34^. Thinner deep layers, on the other hand, may reflect processes of reduced overall cell density that are proceeding at these stages of age and may even contribute to overactivation due to changes in local circuit dynamics and reduced inhibition. This may provide a mechanistic explanation for the altered sensory-evoked functional responses in SI in older humans and mice and would support the ‘altered sensory modulation hypothesis’.

It is worth noting that, in older compared with younger adult mice, PV^+^ cell density was increased as a main effect in all layers (most pronounced in layer IV), whereas, in humans, increased myelination was restricted to middle and deep layers. If our interpretation of the ‘altered modulation hypothesis’ is correct, this suggests that cortical layer inhibition is differently affected in humans and mice with aging. An alternative explanation for the qT1 effect is that myelin sheaths are degrading (that is, becoming less compact) due to sheath splitting and the formation of myelin balloons, which is accompanied by the production of redundant myelin and an increase in oligodendrocytes in older age^27^. Future studies focusing on cytoarchitectural changes in relation to changing myelin patterns, neurovascular coupling and sensory processing can address this: of particular interest would be cross-species longitudinal studies. Whereas longitudinal resting-state MRI studies have shown changes in functional connectivity of somatosensory networks with age in mice^35^, detailed MRI approaches to assess layer IV microstructural changes^36^ have yet to be investigated in mouse models using a longitudinal approach across aging.

The present findings have multiple clinical implications. Our study reveals that the combined investigation of layer-specific thickness, microstructural layer compositions and layer-specific functional readouts, as well as the parallel investigation of humans and mice, uncovers key aspects of cortex organization and potential dysfunction. In Alzheimer’s disease, cortical thinning is related to cell loss and regarded as an early marker of the disease^37^. Whereas we show specific degradation of deep layers with aging, superficial layers may be altered earlier in Alzheimer’s disease. In multiple sclerosis, where neuroinflammation causes myelin damage in specific cortical layers^38^, postmortem investigations in humans and mice showed a decrease in PV^+^ interneurons^39,40^, suggesting an interaction between layer-specific myelination and excitation–inhibition modulation. Losing myelin progressively can result in epilepsy-like brain activity, as inhibition of slow brain waves decreases^41^, and epileptogenic hyperexcitability and lesions often present layer specific^42^. Multimodal layer-specific investigations could uncover underlying mechanisms, also in multiple sclerosis^38^. Investigating participants with congenital or acquired hand loss may provide further insights into the mechanisms of layer-specific plasticity in SI, specifically to disentangle age-related from input-related changes. Although we report a case study of a healthy, middle-aged participant with congenital arm loss in Extended Data Fig. 2 and Supplementary Fig. 5, extended and thorough investigations of this special population may derive key insights into underlying mechanisms. In addition, further studies with higher sample sizes will allow investigating subtle differences between age groups in more detail. The results presented here motivate the investigation of high-precision cortical pathology, allowing for the tailoring of interventions to specific patients.

## Methods

### General procedure

Human data acquisition and analyses were organized in two cohorts: cohort 1 and cohort 2. In Extended Data Table 5, a detailed overview of which data were acquired in which session and used for which analyses is provided.

### Human participants

Cohort 1 is composed of 46 healthy, right-handed volunteers who underwent structural 7T MRI, functional localizers of individual fingers and behavioral tests of tactile finger performance. Due to severe motion artifacts in the MRI data, six participants were excluded, leaving a total of 40 participants for cohort 1 (20 younger adults: 10 females, 21–29 years, mean ± s.d.: 25.1 ± 2.7 years; and 20 older adults: 10 females, 63–77 years, mean ± s.d.: 70.5 ± 4.0 years). Cohort 2 is composed of 38 healthy, right-handed volunteers (21 younger adults and 17 older adults) who underwent behavioral measurements of finger performance, of which 27 volunteers (14 younger adults and 13 older adults) also underwent 7T fMRI with a focused investigation of the index and middle finger representations. Due to severe motion artifacts in the MRI data, six participants were excluded, leaving a total of 21 participants for cohort 2 (11 younger adults: five females, 25–35 years, mean ± s.d.: 28.18 ± 3.06 years; and 10 older adults: three females, 60–80 years, mean ± s.d.: 68.40 ± 6.20 years).

Chronic illness, central acting medications and MRI contraindications (for example, active implants, non-removable metallic objects, tattoos, claustrophobia, tinnitus, consumption of alcohol and drugs and pregnancy) were a priori exclusion criteria for both cohorts. A study-specific health screening revealed no anomalies of sensory perception (for example, numbness, tingling sensations, hypersensitivity and hyperalgesia) and motor control (for example, restricted finger movements) in distal extremities. Given altered sensory processing in string and piano players^47^, no professional musicians were enrolled. Two of 20 older adults in cohort 1 underwent successful carpal tunnel surgery on either the right hand only or on both hands. Their tactile performance was absent of clinical signs (no outliers). Participants reported no other medical conditions. None of the participants in cohort 1 showed signs of cognitive impairments as indicated by the Montreal Cognitive Assessment (MoCA; score ≥26 used as criterion for healthy aging; younger adults mean ± s.d.: 29.0 ± 1.1 and older adults mean ± s.d.: 27.8 ± 2.2)^48^, except for one older adult with a MoCA score of 21. Because this participant performed equally well in the behavioral tasks compared with all other older adults, data were kept in the analysis. Younger and older adults from both cohorts were recruited from the participant bank of the German Center for Neurodegenerative Diseases (DZNE) in Magdeburg, Germany.

We also collected data of *n* = 1 healthy adult with congenital arm loss (male, age 52 years; affected side: right; level of deficiency: complete arm missing from the shoulder; no experience of phantom sensations and pain; cosmetic prosthesis worn more than 8 hours per day; prosthesis never involved in daily life routines) who was recruited from the database of the Central Institute of Mental Health in Mannheim, Germany.

All participants gave written informed consent and were paid for attendance. The study was approved by the ethics committee of the Otto von Guericke University Magdeburg. Data of younger and older adults in cohort 1 were partly published in previous studies^3,10^.

### Human MR scanning

#### Human 7T MRI

7T MRI data were collected on a whole-body 7T MAGNETOM scanner (Siemens Healthcare) and a 32-channel Nova Medical head coil. The following data were acquired: MP2RAGE^49^ with whole brain coverage (for both cohorts: sessions 1 and 6; see Extended Data Table 5 for session indexing): 0.7 mm^3^, 240 sagittal slices, field of view (FoV) read = 224 mm, TR = 4,800 ms, TE = 2.01 ms, inversion time TI1/TI2 = 900/2,750 ms, flip angle (*α*) = 5°/3°, bandwidth = 250 Hz/Px, GRAPPA 2; MP2RAGE with part brain coverage (targeting SI, cohort 1: session 1): 0.5 mm^3^, 208 transversal slices, FoV read = 224 mm, TR = 4,800 ms, TE = 2.62 ms, inversion time TI1/TI2 = 900/2,750 ms, flip angle (*α*) = 5°/3°, bandwidth = 250 Hz/Px, GRAPPA 2, phase oversampling = 0%, slice oversampling = 7.7%; susceptibility-weighted images (SWIs) with part brain coverage (targeting SI, cohort 1: session 1): three-dimensional (3D) gradient-recalled echo (GRE) pulse sequence^50^, 0.5 mm^3^, 208 transversal slices, FoV read = 192 mm, TR = 22 ms, TE = 9.00 ms, flip angle = 10°, bandwidth = 160 Hz/Px, GRAPPA 2, phase oversampling = 0% and slice oversampling = 7.7%. Structural scanning lasted, in total, approximately 60 minutes in session 1 and 20 minutes in session 6.

To acquire fMRI data, we first performed shimming and acquired two echo-planar images (EPIs) with opposite phase-encoding polarity before the functional time series were collected using GRE EPI pulse sequences (cohort 1, session 2: 1 mm^3^, FoV read: 192 mm, TR = 2,000 ms, TE = 22 ms, GRAPPA 4, interleaved acquisition, 36 slices; cohort 1, session 3: 1 mm^3^, FoV read = 212 mm, TR = 2,000 ms, TE = 25 ms, GRAPPA 2, interleaved acquisition, 81 slices; cohort 2, sessions 6 and 7: 0.9 mm^3^, 30 slices, interleaved acquisition, FoV read = 216 mm, TR = 2,000 ms, TE = 22 ms, GRAPPA 4). The phase-encoding EPIs were distortion corrected using point spread function (PSF) mapping^51^. We applied a weighted image combination to combine the distortion-corrected phase-encoding EPIs while controlling for differences in spatial information. Resulting EPIs were motion corrected to timepoint zero. PSF mapping was performed on motion-corrected EPIs to allow geometrically accurate image reconstruction. The same sequence was used for all functional tasks (see below).

#### Human 3T MRI

For cohort 2 (session 8), 3T MRI data were acquired on a Philips 3T Achieva dStream MRI scanner. A standard structural 3D MPRAGE was acquired with the following parameters: 1 mm^3^, 192 slices, FoV read = 192 mm × 256 mm, slab thickness = 256 mm, TI = 650 ms, echo spacing = 6.6 ms, TE = 4.73 ms, flip angle = 8° and bandwidth = 191 Hz/Px.

#### Human physiological data recording

A pulse oximeter (NONIN Pulse Oximeter 8600-FO) clipped to the participant’s left index finger captured the pulse, and a breathing belt captured respiration during fMRI (cohort 1: session 2; cohort 2: sessions 6 and 7). Signals were digitally recorded with a sampling frequency of 200 Hz using an in-house-developed setup (National Instruments USB 6008 module with a Honeywell 40PC001B1A pressure sensor).

### Human fMRI tasks

For both human cohorts, independently controlled, MR-compatible modules were used for tactile stimulation of the fingers^3^ (each with eight piezoelectric-controlled pins arranged in a 2 × 4 matrix, covering 3.5 × 8.5 mm^2^ of skin; Quaerosys, http://www.quaerosys.com; Fig. 1a). Individually adjusted vibrotactile stimulation was applied at a frequency of 16 Hz. Participants were asked to look at a centrally presented fixation cross and count randomly occurring stimulation pauses and report them after each run.

#### Five-finger functional localizer (cohort 1)

In session 2 of cohort 1, we applied a previously established tactile stimulation paradigm to localize the fingers of the right hand in contralateral SI^3,10^. Five modules were used to stimulate the five fingertips of the right hand (one module each), with two pins raising at a time and 16 pin combinations per second^3^. First, a phase-encoded protocol was applied (two runs of 20 cycles; each fingertip stimulated 20 times for 5.12 seconds) in forward order (thumb to little finger, 50% forward-run first) and reverse order (little finger to thumb, 50% reverse-run first). One run took 8 minutes and 32 seconds (256 scans, TR of 2 seconds). Second, a blocked-design protocol was used to stimulate the fingers in a pseudo-random order (two runs; six conditions: stimulation to thumb, index, middle, ring, little finger and no stimulation). One run took 6 minutes and 56 seconds (each fingertip was stimulated 10 times for 2 seconds, followed by a 22-second resting phase; interstimulus interval (ISI): 2 seconds in 70% of trials and 6 seconds in 30% of trials, counterbalanced between fingers; 208 scans). Total scan time was approximately 35 minutes. The localizer was used to localize the five fingertip representations in SI in the left hemisphere for younger and older adults.

#### Hand-and-face functional localizer (cohort 1)

In session 3, cohort 1 underwent a blocked-design paradigm where participants moved the left or right hand, the left or right foot or the tongue. Participants were trained outside the MR scanner and wore fingerless braces to stabilize the hand while carrying out the movements. Movements were carried out for 12 seconds followed by a 15-second rest period. Movements were repeated four times, each resulting in 20 trials and taking approximately 9 minutes in total. This paradigm was used previously to localize hand and face areas^5^. The localizer was used to extract cortical thickness in major body part representations of SI and to detect low-myelin borders between the hand and the face. For the one-handed adult, we applied a modified version of the original motor paradigm using mental imagery of finger movements for the missing limb (Supplementary Fig. 1).

#### Two-finger functional localizer (cohort 2)

In session 6 of cohort 2, the modules were used to stimulate the distal phalanges and intermediate phalanges of the index and middle finger of the right hand. One module was attached to each phalanx using a custom-built, metal-free applicator that fitted individual finger sizes (Fig. 1a). To minimize adaptation-related differences in map activity between individuals, three randomly chosen pins were raised once at a time, yielding 56 pin combinations per second. Participants underwent two blocked-designed runs to localize the representation of the distal and intermediate phalanges of the index and middle finger in SI in the left hemisphere for each individual. The blocked-design run comprised three conditions, including stimulation of the index finger, stimulation of the middle finger and a rest condition with no stimulation. Each finger was stimulated for 8 seconds in a pseudo-random sequence, where one finger was stimulated maximally two times in a row. In 70% of the trials, there was a 4-second pause, and, in 30% of the trials, there was an 8-second pause between two subsequent stimulations, counterbalanced across fingers. Each finger was stimulated 20 times. One run comprised 264 scans and lasted for 8 minutes and 48 seconds. The run was repeated twice, lasting approximately 20 minutes in total.

#### Two-finger functional response profile (cohort 2)

In session 7, cohort 2 underwent 12 phase-encoded runs, which were used for percent signal change and pRF modeling analyses. The phase-encoded runs included three different conditions: (1) stimulation of only the index finger, (2) stimulation of only the middle finger and (3) stimulation of both the index finger and the middle finger together. Each condition comprised four runs, each consisting of eight stimulation cycles and two rest conditions of 32 seconds (one before and one after stimulation). Each stimulation cycle lasted 32 seconds, and stimulation was applied to each section of the phalanx four times for 8 seconds. Half of the stimulation runs of each condition were delivered in a forward order (finger top → finger bottom), the other half in a reverse order (finger bottom → finger top) (Fig. 1a). Half of the participants of each age group started with the forward-order run, the other half with the reverse-order run. One run comprised 160 scans (128 scans for stimulation and 32 scans for rest), lasting 320 seconds (TR = 2 seconds). Participants were instructed to covertly count short randomly distributed interrupts embedded in the tactile stimulation (duration 180 ms; see Liu et al.^3^). There was the same number of gaps in each run (32 gaps in total). All phase-encoded runs took approximately 60 minutes.

#### Resting state (cohorts 1 and 2)

For both cohort 1 and cohort 2, we acquired resting-state data in a 5-minute scan while participants looked at a fixation cross and were asked to think about nothing in particular.

### Human MRI analyses

#### Reconstruction of QSM images (cohort 1)

QSM data were first reconstructed from SWIs (that is, magnitude and phase images) using the Bayesian multi-scale dipole inversion (MSDI) algorithm^52^ as implemented in QSMbox (version 2.0, freely available for download at https://gitlab.com/acostaj/QSMbox). No normalization was applied to QSM values, because aging effects are assumed to be similar for normalized and non-normalized 7T MRI data^16^.

#### Structural image processing (cohorts 1 and 2)

First, the quality of collected images was evaluated. QSM and qT1 images of *n* = 6 participants from cohort 1 and qT1 images of *n* = 2 participants from cohort 2 were excluded due to severe motion artifacts. Only data showing no artifacts or mild truncation artifacts (not affecting SI) were processed.

For cohort 1, we used the structural image preprocessing approach as described in Doehler et al.^10^, employing CBS Tools (version 3.0.8) as a plugin for MIPAV (version 7.3.0). In short, after background noise cleaning, inhomogeneity correction and skull stripping, we co-registered qT1 slab images to upsampled qT1 whole brain images (combining linear and nonlinear registration using ANTs version 1.9.x-Linux, embedded in CBS Tools) in one single step (nearest neighbor interpolation). To register QSM slab images to qT1 images, a combination of rigid and affine automated registration was applied using ITK-SNAP (version 3.8.0). Registered qT1 slab and upsampled qT1 whole brain images were fused. Resulting images were skull stripped, and dura was removed. Dura estimates were manually refined where required to ensure complete removal of non-brain matter from the region of interest (ROI). For cohort 2, the images were sent to a pipeline including background noise cleaning, skull stripping and inhomogeneity correction. Background noise in all T1-weighted images was removed using the code of José P. Marques (https://github.com/JosePMarques/MP2RAGE-related-scripts) and the method described in O’Brien et al.^53^. Skull stripping was performed on T1-weighted images using the FreeSurfer (version 7.3.0) mri_synthstrip routine. Inhomogeneity correction was done for both qT1 and T1-weighted images using the segment routine as implemented in SPM12 (Statistical Parametric Mapping, Wellcome Department of Imaging Neuroscience, University College London) in MATLAB (MathWorks, R2018b (2018)).

For both cohorts, we used the TOADS algorithm^54^ to segment the cortex from the rest of the brain before we applied the CRUISE algorithm^55^ to estimate tissue boundaries using the level set framework^56^. Cortex estimates were thresholded between the maximum of the inner and outer level set images (−2.8 and −0.2, respectively). The cortex was divided into 21 cortical depths using the validated equivolume model^57^. Intracortical qT1 values were used as proxy for myelin^14^, QSM values were used as proxies for iron (pQSM)^15^, calcium/diamagnetic contrast (nQSM)^58^ and mineralization (aQSM)^16^. All values were sampled along the extracted cortical depths in reference to individual cortical folding patterns. Individual cortical surfaces were estimated based on level set images of the middle cortical depth. Layer-specific quantitative values of the original high-resolution qT1 image (cohort 1 and cohort 2) and the QSM image (cohort 1) were mapped onto the inflated cortical surfaces (method of closest point)^59^. Sampled values at 21 cortical depths were extracted from the five fingertip representations (five-finger localizer) and from the face–hand representation (hand and face localizer) in area 3b for cohort 1 and from the index finger and middle finger representations (two-finger localizer) in area 3b for cohort 2. Quantitative values were averaged across vertices within individuals (giving one value per body part and depth).

For 3T MRI processing (T1-weighted 3D MPRAGE), csurf (http://www.cogsci.ucsd.edu/~sereno/.tmp/dist/csurf) recon-all (implanted from FreeSurfer (version 7.3.0) (http://surfer.nmr.mgh.harvard.edu/)) was used for brain segmentation and cortical surface reconstruction. As a fully automated processing pipeline, recon-all performs steps including intensity correction, transformation to Talairach space, normalization, skull stripping, subcortical and white matter segmentation, surface tessellation, surface refinement, surface inflation, sulcus-based nonlinear morphing to a cross-subject spherical coordinate system and cortical parcellation^60^. Skull stripping, segmentation and surface inflation quality were checked for each participant.

#### Definition of layer compartments

First and second derivatives of depth-dependent qT1 profiles (sampled across 21 cortical depths) were calculated using the gradient function as implemented in MATLAB R2017b. Depth-dependent structural profiles were averaged based on a previously introduced approach^10^, using ex vivo and in vivo validated myelin profiles of area 3b^14^ to identify anatomically relevant layer compartments in in vivo MRI data (Fig. 2b,d). After removing the two deepest layers in both age groups, minima and maxima of the first derivative of raw qT1 profiles indicated three data-driven layer compartments: an inner, a middle and an outer compartment. Based on Dinse et al.^14^, we assume that the input layer IV is located in the middle compartment and that deep layers V/VI are located in the inner compartment. However, we note that these compartments are based on in vivo MRI data and may, therefore, not match exactly with the anatomical layers as described by ex vivo myeloarchitecture and cytoarchitecture.

#### Functional data surface mapping (cohorts 1 and 2)

All functional images were registered to the qT1 image (0.5-mm isotropic resolution for cohort 1 and 0.7-mm isotropic resolution for cohort 2) using the automated registration tool in ITK-SNAP (version 3.6.0, non-rigid transformation, 9 degrees of freedom). Manual refinement was applied where required to ensure registration accuracy. Registration matrices were applied to the functional parameter maps (that is, for cohort 1: five-finger localizers, hand–face localizer, pRF center location maps and ECMs; for cohort 2: two-finger localizer and two-finger functional response profiles) in a single step (ANTs, version 2.1.0, nearest neighbor interpolation). All ROI analyses were performed on non-smoothed functional data (in original individual space) before statistical maps were registered to individual structural data space. Area 3b masks (see below for definition) were applied to all functional data of cohort 1, and ROI masks resulting from functional localizer were applied to the functional parameter maps of cohort 2. Registered individual functional parameter maps were mapped onto the individual cortical surfaces (method of closest point). The most superficial 20% of cortical values were excluded (to account for superficial veins affecting the BOLD signal). The mean of the remaining cortical values (covering 20–100% of cortical depth) was used to compute statistics independent of cortical depth.

Functional data of cohort 2 were also registered to the 3T T1-weighted image (same image used for recon-all brain segmentation) using csurf tkregister (12 degrees of freedom, non-rigid registration). Here, the *x*, *y* and *z* location of each surface vertex was mapped into functional voxel coordinates with the obtained registration matrix. The floating point coordinates of points at varying distances along the surface normal to a vertex were used to perform nearest neighbor sampling of the functional volume voxels (that is, the 3D functional data were associated with each vertex on the surface by finding which voxel that point lay within).

#### Definition of area 3b (cohorts 1 and 2)

For cohort 1, where analyses focused on structural mapping, area 3b was manually delineated based on an operational definition using anatomical landmarks extracted from cytoarchitectonic^61^, fMRI^62^ and multimodal parcellation studies^63^—that is, following a standardized procedure that was used previously^5,10,64^. Resulting masks cover the anterior wall of the postcentral gyrus (mainly covering area 3b and parts of area 3a). All masks were plotted in reference to co-registered FreeSurfer labels (normalized probabilistic maps of area 3a and area 3b) on the individual cortical surfaces to allow comparability (Supplementary Fig. 4).

For cohort 2, where analyses focused on functional mapping, area 3b and the hand area were defined for each individual based on the atlas provided in csurf^65^.

#### Estimating body part representations (cohorts 1 and 2)

The data quality was evaluated to ensure that there were no severe artifacts for both cohorts, and data showing severe artifacts (that is, *n* = 4 participants in cohort 2) were excluded. Data of cohort 1 were slice-time corrected using SPM8 (Statistical Parametric Mapping, Wellcome Department of Imaging Neuroscience, University College London). Data of cohort 2 were slice-time corrected, and spatial smoothing was applied to the blocked-design data with 1-mm kernel width using SPM12.

For cohort 1, we calculated first-level fixed-effects models for the two blocked-design runs (session 2) using a general linear model (GLM) on individual data as implemented in SPM8. BOLD activation driven by each finger’s stimulation was modeled as an independent measure, because each finger was treated individually^66^. Five regressors of interest (stimulation to thumb, index, middle, ring and little finger) were modeled per session, resulting in five linear contrasts (for example, the contrast [−1, 4, −1, −1 −1] for stimulation to index finger). Peak clusters of *t*-values were extracted (*P* < 0.01, minimum cluster size of *k* = 3). Resulting *t*-value maps were taken forward for surface mapping and were used to extract seeds for the low-myelin border analysis.

We used Bayesian pRF modeling to localize the five fingertips^3,10^. Phase-encoded fMRI data (cohort 1, session 2) were used to perform a two-stage analysis using the BayespRF Toolbox (https://github.com/pzeidman/BayespRF) in MATLAB (SPM12, MATLAB R2017b). We conducted a first-level GLM analysis, constructing five regressors for the five fingers of the right hand. Only voxels passing a threshold of *P* < 0.05 uncorrected were included in the second stage of pRF modeling^3^, which was performed on a voxel-by-voxel basis on the inferior-to-superior dimension (*x* dimension) of topographic alignment, using a Gaussian response function and a posterior model probability >0.95 (code available for download at https://gitlab.com/pengliu1120/bayesian-prf-modelling.git)^3^. The analysis was restricted to area 3b to reduce processing time. We extracted pRF center location (x) to locate activated finger-specific voxels (finger-specific ROIs) and pRF width (*σ*, s.d. of the Gaussian response function) to estimate the pRF size of activated voxels in one-dimensional stimulus space. The most superficial 20% of the remaining data points were disregarded to minimize the effects of superficial veins. The resulting pRF center location maps (restricted to area 3b) served as localizer for individual finger representations. A ‘winner-takes-it-all’ approach was applied to sample vertices only once. Overlapping vertices (introduced by mapping splitted single finger pRF center location maps) were exclusively assigned to the finger map with the highest variance explained (obtained from pRF modeling). Combining the five single finger maps to one ROI defined the hand area in area 3b of younger and older adults.

For the motor paradigm (cohort 1, session 3), we used first-level GLM analysis implemented in SPM12 to estimate functional activation maps (*t*-value maps) of tongue and finger movements using contrast estimates for each body part. We thresholded peak clusters at *P* < 0.01 with a minimum size of *k* = 3. Resulting *t*-maps were taken forward for surface mapping. For the participant with congenital arm loss, we estimated *t*-value maps of finger movements or imagining finger movements of the left hand. Resulting *t*-maps were taken forward for surface mapping and were thresholded in surface space. Hand and face activation maps were used to localize the hand–face area in younger and older adults and in the participant with congenital arm loss. A ‘winner-takes-it-all’ approach was applied to the extracted values to sample vertices only once. For the participant with congenital arm loss, the lowest 30% of *t*-values were removed to ensure comparable map sizes within and between participants. To exclude spatial outliers, vertices located ±2 s.d. away from the location of the main cluster (in *z* direction and in *y* direction) were removed from the final data.

For cohort 2, we also calculated first-level fixed-effects models for the functional localizer of index and middle finger using SPM12. Two regressors of interest were modeled explicitly (stimulation to index finger and stimulation to middle finger), and the rest condition was modeled implicitly. The linear contrast estimate for index and middle finger was computed: *F* contrast [1 0/0 1], replicated for each session. On the individual subject level, voxels that survived a significance threshold of *P* < 0.05 (uncorrected) were mapped onto individual cortical surfaces generated by csurf. The overlapping area between the hand area^65^ and the index and middle finger representation in area 3b of each individual served as ROI masks for further analyses.

#### Resting-state analyses (cohort 1)

Resting-state functional data (session 2) were corrected for pulse-induced and respiration-induced noise. To prepare the physiological data for noise correction and to remove acquisition artifacts, we used the open-source Python-based software ‘PhysioNoise’^67^. Resulting respiratory and cardiac phase data were used to correct the resting-state time series for pulse-induced and respiration-induced noise by performing RETROspective Image CORrection (RETROICOR) on a slice-by-slice basis^68^. Residuals were taken as cleaned data to regress out motion-related noise parameters (extracted from the raw data) using the program vresiduals as implemented in LIPSIA (freely available for download at github.com/lipsia-fmri/lipsia)^69^. The resulting data were high-pass filtered at 0.01 Hz (allowing frequencies faster than 0.01 Hz to pass) and smoothed (Gaussian kernel with a full width at half maximum of 2 mm) using the program vpreprocess implemented in LIPSIA. For *n* = 6 participants, physiological data were not successfully recorded due to loosening the pulse oximeter and/or breathing belt during scanning, which interrupted successful data sampling. For *n* = 5 participants, severe motion artifacts were detected in the resting-state data. Therefore, resting-state analyses are presented for *n* = 29 (14 younger and 15 older) participants only.

ECMs were calculated in native space as a measure of network centrality (that is, maps reflecting the degree of connectedness of nodes within a network^70^) using the program vecm as implemented in LIPSIA^69^. The method of rectified linear unit correlation (RLC)^71^ was applied, which is suitable for ultra-high-resolution fMRI data.

#### Cortical thickness estimation (cohort 1)

The profile geometry module from the CBS Tools (version 3.0.8) for MIPV (version 7.3.0) was used to calculate overall cortical thickness across cortical depths (after removing the two deepest cortical depths) and layer-specific cortical thickness of extracted layer compartments.

#### Low-myelin border detection (cohort 1 and one-handed person)

To investigate low-myelin borders in human area 3b, a multimodal surface-based mapping approach was developed and applied to each individual dataset. First, multidimensional sampling (inferior to superior, anterior to posterior) of layer-specific qT1 values was performed within area 3b (PyVista implementation of the Dijkstra algorithm^72^)^10^. The peak activation of the thumb (as identified by tactile stimulation using five-finger localizer task) served as seed region to sample geodesic paths in inferior-to-superior direction, connecting the upper face representation with the little finger representation. Start and end points were defined along the *y* axis of thumb and small finger activation peaks and approximately 15 mm (geodesic distance on shortest path) below the thumb activation peak (estimation derived from the location of the forehead as described previously^73^ and by ensuring that all sampling points are situated within the face area). Considered vertices were scattered within one vertex-to-vertex distance of approximately 0.28 mm around the *y* axis. Only in cases where the underlying qT1 pattern did not match *y* axis sampling, start and end points were defined along the *x* axis.

Five equally distant geodesic paths were sampled for each participant (Fig. 3a) to extract qT1 values from middle cortical depth (where the detection of low-myelin borders in area 3b is expected^9^). Peak detection was then performed on the five extracted qT1 signals (Fig. 3a). To control for a possible gradient in cortical myelin content along the inferior-to-superior axis^9^, all qT1 signals were detrended before the find_peaks algorithm from the SciPy signal processing toolbox (version 1.10.1) was applied to find the most prominent peaks (local maxima with a prominence >2 s.d. from the mean of absolute detrended qT1 values) in each detrended qT1 signal by comparing neighboring values. Resulting peaks were considered a low-myelin border (reflected by a row of high qT1 values) when they occurred in at least three of five detrended qT1 signals based on a nearest neighbor approach. The nearest peaks on first and second neighbor signals were grouped together based on geodesic distances. Peaks with a geodesic distance of less than 5 mm were considered near. Resulting low-myelin borders were then back-projected to individual cortical surfaces and visualized in reference to individual pRF finger maps to categorize low-myelin borders in hand–face borders and within-hand borders. Finally, several different features were extracted for each peak, including prominence, full width at half maximum, qT1 intensity, ECM, signed QSM intensity, nQSM intensity, pQSM intensity and aQSM intensity. For each feature, all values belonging to a single border were averaged to obtain one feature vector for each border. Feature vectors of within-hand borders were further averaged across all within-hand borders. In this way, we extracted two feature vectors (one for within-hand borders and one for the hand–face border) in each individual, which were used to calculate group statistics (between younger and older adults).

For the participant with congenital arm loss, the activation peak of the hand representation (as identified by movement of the intact hand and imagery of movement of the missing hand) served as seed region to sample geodesic paths in inferior-to-superior direction, connecting the upper face representation with the superior border of the hand representation. Start and end points were defined along the *y* axis at the superior border of the hand representation and approximately 15 mm (geodesic distance on shortest path) below the hand activation peak (estimation derived from the location of the forehead as described previously^73^ and by ensuring that all sampling points are situated within the face area).

#### Percent signal change calculation (cohorts 1 and 2)

Individual time series of the averaged forward-order and reverse-order runs from phase-encoded protocol in the five-finger localizer task (session 2 for cohort 1) and functional response profile task (session 7 for cohort 2)^3,66^ were used to calculate mean response amplitudes by using csurf (http://www.cogsci.ucsd.edu/~sereno/.tmp/dist/csurf). The time series were averaged timepoint by timepoint by reversing the direction of time on a scan-by-scan basis, as the time series of different cycle directions (forward or reverse) were mirror symmetric to each other. The time-reversed cycle direction was time shifted before averaging by 4 seconds (two TRs) to compensate for hemodynamic delay. Averaging was performed in three dimensions without any additional registration. Neither normalization nor smoothing was performed on the data during this procedure. Discrete Fourier transformations were performed on the timecourse of each 3D voxel, before calculating the phase and the significance of the periodic activation. Cycles of stimulations (20 for cohort 1 and eight for cohort 2) were used as input frequencies. Frequencies lower than 0.005 Hz (known to be dominated by movement artifacts) were excluded, whereas higher frequencies up to the Nyquist limit (1/2 the sampling rate) were included. For display, a vector was generated whose amplitude was the square root of the *F*-ratio calculated by comparing the signal amplitude at the stimulus frequency to the signal amplitude at other noise frequencies and whose angle was the stimulus phase. To estimate mean response amplitudes of the ROIs (in percent), we estimated the discrete Fourier transform response amplitude (hypotenuse given real and imaginary values) for each voxel. This value was multiplied by 2 to account for positive and negative frequencies, again multiplied by 2 to estimate peak-to-peak values, divided by the number of timepoints over which averaging was performed to normalize the discrete Fourier transform amplitude and divided by the average brightness of the functional dataset (excluding air). Finally, the value was multiplied by 100 to estimate the percentage response amplitude^3,66^.

The data were sampled onto the individual FreeSurfer surface for each participant. Note that those analyses were used as two-finger localizers but not for layer-specific analyses. Clusters that survived a surface-based correction for multiple comparisons of *P* < 0.05 (correction was based on the cluster size exclusion method as implemented by surfclust and randsurfclust within the csurf FreeSurfer framework)^74^ and a cluster-level correction of *P* < 0.001 were defined as significant. For each participant and condition, the complex-valued phasing-mapping data (real and imaginary values) were sampled onto the individualized inflated 3D cortical surface, and the values within the ROI were extracted. Mean percent signal changes of the tactile maps of each condition were calculated.

After registering the two-finger functional response profile estimates (the complex-valued phasing-mapping data—that is, real and imaginary maps) to qT1 images, percent signal change was calculated for each of 21 cortical depths, which led to a curve drawn across the cortical depth. The curves were normalized for younger and older adults, respectively, using minimum–maximum normalization. The resting-state BOLD signals were also extracted for each cortical depth and normalized using minimum–maximum normalization. The normalized BOLD signals were then used to calibrate the normalized percent signal change to control for baseline fluctuations. The central peak and surrounding signal decay were detected by calculating the area under the curve of the calibrated percent signal change curve across cortical depth.

#### pRF mapping (cohort 2)

pRF mapping of the index and middle fingers was performed on phase-encoded data (session 7) using a MATLAB-based toolbox, SamSrf version 9.4 (freely available for download at https://github.com/samsrf/samsrf). The toolbox provides a generic framework to model pRFs with stimulus space in any dimension^75^. The phase-encoding dataset was used for pRF mapping, with an additional rest condition to provide baseline information. The forward-order runs and reverse-order runs were converted into FreeSurfer MGH format and projected onto the recon-all reconstructed surface. After converting from MGH to .mat format, the data were concatenated and averaged to increase the signal-to-noise ratio. The data were *z*-scored and linearly detrended. A stimulus aperture was created to define the somatosensory space. To represent the finger areas that were stimulated, the aperture was set up with the dimension of 100 × 100 × 320 (320 = number of TR). Four finger areas were constructed (two sections in distal phalanx and two sections in intermediate phalanx), with each one associated with each of the stimulation units, based on the stimulation timing. Modeling ROI was defined manually by choosing vertices in the inflated surface model with restricting *y* coordinates from −55 to 5 and *z* coordinates from 30 to 80 in FreeSurfer space, which covered the full SI area. Because the stimulation was applied along one dimension of the fingers, a two-dimensional Gaussian tuning curve vertical model was used to perform the model fitting only on the *y* dimension (within-digit dimension), with the *x* dimension set as a constant. The pRF profile determines the predicted response for a given location. The predicted time series is convolved with a canonical hemodynamic response function (HRF).

The parameter modeling employed a two-stage coarse-to-fine procedure to obtain the best possible fit of the predicted time series with the observed data. The coarse fit process involved an extensive grid search by correlating the observed time series against a set of predicted time series derived from a combination of *y*_0_ and *σ*, covering the plausible range for each parameter. The search space was set at [−1.25, 1.25], which covers more than the stimulus space [−1, 1]. The parameters giving rise to the maximal correlation can survive and be sent to the second stage. The fine fit is an optimized procedure to refine the parameters by further maximizing the correlation between observed and predicted time series. The noise ceiling was calculated as an estimate of the maximum goodness of fit that could theoretically be achieved from the data of each voxel^75^, using a similar procedure as Urale et al.^76^. The Spearman–Brown prophecy formula^77^ was used to account for the accurate estimate of the true reliability of the time series. Six other parameters were estimated during modeling, including the center location (*μ*), the sizes (*σ*), goodness of fit (*R*^2^), normalized goodness of fit (n*R*^2^), baseline and beta (*β*). *R*^2^ is the coefficient of determination of the correlation between the observed and predicted time series, and n*R*^2^ is the normalized coefficient of determination relative to the noise ceiling. During modeling, baseline (intercept) and *β* parameters (that is, BOLD response amplitudes) were estimated for each voxel. After extracting the modeled parameters within the ROI, the resulting data were cleaned by preserving only those vertices with positive *β* values. The average pRF size (*σ*) of each condition and of each participant was calculated based on the cleaned data.

### Human behavioral measurements

#### Tactile detection task (cohort 1)

The detection of finger touch was assessed using fine hair stimuli (sessions 4 and 5). We used a subset of standardized tactile monofilaments (Semmes–Weinstein monofilaments; Baseline, Fabrication Enterprises) to apply different mechanical forces (0.008*g*, 0.02*g*, 0.04*g*, 0.07*g*, 0.16*g*, 0.4*g*, 0.6*g*, 1.0*g*, 1.4*g*, 2.0*g*, 4.0*g*, 6.0*g*) to the skin surface of the fingertips (Fig. 1). Stimuli were manually applied (guided by auditory instructions via headphones, controlled by the Psychophysics Toolbox extension in MATLAB R2017b) to a predefined skin area (circle with a diameter of approximately 2 mm), touching the skin surface at an angle of approximately 90°, for 1 second^78^. All participants listened to white noise via headphones. The right hand (palm facing upwards) was fixated on a small pillow behind a paper wall, preventing participants from seeing their own hand and the experimenter.

In a two-alternative forced choice (2AFC) paradigm, participants were asked to choose the time intervals that contained the stimulation (randomly applied in the first or second interval)^79^. Application of tactile monofilaments followed a 3-down/1-up staircase approach with two interleaved staircases, one starting at 0.02*g* and the other starting at 0.4*g*^80^. The experiment was finished when for the last 30 trials the standard deviation in stimulus intensity was one step or less^79^ or when the maximum number of 100 trials was reached. The participant’s tactile detection threshold was defined as the mean stimulus intensity across reversal points (change of response from correct to incorrect or incorrect to correct) within the period of stable performance (that is, the last 10 trials). The experiment took approximately 12 minutes per finger.

Before averaging, stimulus intensities were transformed logarithmically on a 1/10th milligram scale (log_10_ 0.1 mg). Lower values indicate higher tactile sensitivity to mechanical forces. We also estimated the skin indentation in millimeters based on the examined detection threshold. Detection thresholds were taken as proxies of corresponding values in milliNewton (mN) that were provided by the manufacturer. Afterwards, the skin indentation *δ* was calculated according to the following equation^81^:

$$\delta =[(F/A)(1/b)] \times {\hat{\delta }}$$

where *F* is the estimated force in Newton (N), *A* (=0.2368 N) and *b* (=2.0696) are material/structural constants, and $\hat{\delta }$ (=1.00 mm) is the reference indentation^81^. Finally, the result (*δ*) was multiplied by 3 to get an indentation value clearly above threshold, which was used to increase the amplitude of pin movement in the 2PD task. All calculations were performed in MATLAB R2017b.

#### Finger discrimination task (cohort 1)

The same Semmes–Weinstein monofilaments were used to apply tactile stimulation, targeting the same stimulation sites as described for the tactile detection task. In a 5AFC design, tactile stimulation (lasting 1 second) was applied to one of five possible fingertips (session 5; Fig. 1). Participants were asked to name the finger where they felt the touch. Answers were given verbally within a limited response interval (lasting 7 seconds). In case participants perceived no touch (note that tactile stimulation was applied at individual detection thresholds and was, therefore, expected to be perceived in only approximately 50% of the cases), they were motivated to guess. Each fingertip was stimulated 20 times, using unique pseudo-randomized sequences (with fingertips being stimulated not more than two times in a row). To extract the finger discrimination sensitivity (sensitivity of one finger being correctly discriminated from other fingers), we applied signal detection theory and calculated the *d*-prime as bias-free index of discrimination sensitivity^82^ by computing the amount of times a specific finger was touched and detected (hit) or was not touched but falsely detected (false alarm). Hits and false alarms were first converted to *z*-scores before subtracting false alarms from hits. *d*-prime values were obtained for each finger separately.

#### 2PD task (cohort 1)

We assessed the tactile discrimination performance of the right index finger^83^ (session 4; Fig. 1). Stimulation was applied by two rounded pins (diameter = 0.4 mm) simultaneously touching the surface of the fingertip. A custom-made, fully automatic stimulation device moved the pins up and down, controlled by the software package Presentation (version 16.5, Neurobehavioral Systems). The amplitude of pin movement was adjusted to the individual detection threshold (as assessed before in the tactile detection task) but was set to at least 1.2 mm. Spacing between pins ranged from 0.7 mm to 2.8 mm (in steps of 0.3 mm) for younger adults and from 0.7 mm to 6.3 mm (in steps of 0.8 mm) for older adults. A single pin was included as control condition. Pin spacing was vertically adjusted by rotating a disc containing all nine pin spacing conditions. In a 2AFC paradigm (‘one pin’ versus ‘two pins’), pin spacing conditions were pseudo-randomly presented. Participants were instructed to give the answer ‘two pins’ only if they were certain. The right index finger was fixated on the stimulator, and the hand was covered by a white box during the task to prevent effects caused by seeing the stimulated finger^84^. Each task block included 90 trials (10 repetitions per pin condition). To prevent order effects, unique sequences of pin spacing conditions were used per participant and run. All participants completed two runs. Intertrial intervals were pseudo-randomized and varied between 1 second and 5 seconds. 2PD thresholds were calculated per participant and run. Answers ‘two pins’ were fitted as percentages across ascending pin distances (for example, 0.7–2.8 mm). A binary logistic regression was used to fit the data using the glmfit function (iterative weighted least square algorithm) from the Statistics Toolbox as implemented in MATLAB R2017b. The 2PD threshold was taken from the pin distance where the 50% level crossed the fitted sigmoid curve^83^. Lower values indicate higher spatial acuity.

*z*-transformed false alarm and hit rates were calculated for each participant to derive *d*-prime values as bias-free indices of 2PD sensitivity. Hit rates were calculated as the proportion of ‘two pins’ responses when the stimulus indeed consisted of two pins. False alarm rates were calculated as the proportion of ‘two pins’ responses when the stimulus consisted of only one pin. False alarm rates were adjusted to 0.1 by default, if no false alarm was detected^83^.

#### Precision grip task (cohort 1)

Sensorimotor integration was assessed with a custom-made pressure sensor that was held between the thumb and index finger of the right hand, adjusted to individual strength^85^ (session 4; Fig. 1). Reference forces that were to be matched ranged from 5% to 25% of the individual maximum grip force to avoid muscle fatigue^85^. Participants solved a visuo-motor matching task^85^, demanding them to continuously adjust the grip force. Applied forces were sampled at a frequency of 100 Hz and projected on screen at a refresh rate of 60 Hz. The task was controlled by the software package Presentation (version 16.5, Neurobehavioral Systems). Each task repetition contained a unique pseudo-randomized sequence of 10 position changes at five different amplitudes (5%, 10%, 15%, 20% and 25% of maximum grip force), leading to a mean frequency of 0.25 Hz. After a period of task familiarization^85^, all participants performed the task for a total duration of 20 seconds. One run contained 15 trials divided by intertrial intervals of 10 seconds, leading to a total duration of approximately 8 minutes per run. All participants performed two runs, separated by a 5-minute resting period. After each trial, participants received feedback about their individual performance level on screen. We monitored the time (in seconds) that the controllable bar was within a given percentage above (2.5%) and below (2.5%) the target line (upper edge of the reference bar)^85^. Higher values reflect better sensorimotor integration.

#### Tactile detection task (cohort 2)

We measured the tactile detection threshold of the index and middle finger of the right hand using the same MR-compatible piezoelectric stimulator used during MR scanning (session 9). The tested hand was occluded from view, and the module was mounted below the finger. The tactile threshold was detected with a 2AFC task. At each trial, two intervals were presented with only one of them containing a stimulation, which was one pin rising up during a stimulation with a certain amplitude, lasting for 1 second. Participants were asked to detect the stimulation interval by pressing the respective key on the keyboard in a self-paced manner (‘1’ or ‘2’). A randomized sequence (different for each participant) was used to determine which interval contained the stimulation. The adaptive thresholding procedure followed a 3-down/1-up staircase algorithm. For each finger, the stimulation amplitude started at 0.73 mm. Every time the participant chose the correct interval three times in a row, the amplitude went down for 0.03 mm, whereas, if the participant chose the wrong interval, the amplitude went up for 0.03 mm. Participants performed the task until an accuracy above 80% was reached. The task took 45–60 minutes.

#### Texture roughness discrimination (cohort 2)

The texture roughness discrimination test was used to detect individual tactile sensation of surface roughness (session 9; Fig. 1). The test comprises a plastic board with 15 columns, and the size of each column is 1.0 × 1.5 cm. Each column consists of small pins arranged with densities at different levels, resulting in a roughness ranking from rough (level 1) to smooth (level 10). During testing, participants were asked to sit on a chair with the arm positioned on a foam cushion. The tested hand was occluded from view. At each trial, two intervals were presented containing stimulation with two different roughness stimuli. Participants were asked to detect the column with higher roughness (that is, less density). They were asked to press the respective key on the keyboard in a self-paced manner (‘1’ or ‘2’). For stimulus application, the experimenter followed auditory instructions via headphones. Neither the hand nor the experimenter was visible to the participant during testing. The plastic board was positioned under the participants’ right hand during the stimulation and withdrawn afterwards. The test was composed of two conditions, each including 30 trials. During condition 1, tactile sensation of the index finger was tested; during condition 2, tactile sensation of index and middle finger combined was tested. The stimulation pairs were chosen under three different conditions, with the roughness level increasing consecutively (for example, level 3 and level 4) or in increments of two (for example, level 3 and level 5) or three (for example, level 3 and level 6). Stimulation pairs too easy or too difficult to distinguish were excluded. The stimulation order for each pair and for the sequence was randomized for each participant. The test took approximately 45–60 minutes.

#### Hand dexterity

Two standard tests (that is, the Grooved Pegboard Test and the O’Conner Finger Dexterity Test) were used to test individual levels of hand motor function^3^, each consisting of two conditions (session 9; Fig. 1). During one testing condition, participants were asked to use only their thumb and index finger of the right hand, whereas, during the other testing condition, they were asked to use their thumb, index finger and middle finger.

We also performed the Small Motor Test, which also consisted of two conditions (session 9; Fig. 1). During condition 1, participants were asked to use only their thumb and index finger of the right hand to pick up the pins, whereas, during condition 2, they were allowed to use their thumb, index finger and middle finger. It was composed of hexagonal-shaped nuts and small metal pins. The task was to pick up a pin using two fingers or three fingers to fill in the hole on a hexagonal-shaped nut held by the left hand and, after success, to then move on to the next pair. We measured the number of pins and nuts that were successfully paired with each other (*n*).

### Human data statistical analyses

For cohort 1, all statistical analyses were carried out in R (version 4.2.2, R Core Team, 2022). All sample distributions were analyzed for outliers using box plot methods and tested for normality using the Shapiro–Wilk test. Homogeneity of variances was tested with Levene’s test. We applied two-sided tests. The significance level was set to 0.05. Bonferroni-corrected thresholds were applied for multiple testing to correct for family-wise error accumulation. To account for skewed and heteroscedastic data, we calculated robust permutation tests^86^. Group differences between younger and older adults (that is, cortical thickness and myelin border analyses) were investigated with independent-sample random permutation Welch *t*-tests (100,000 Monte Carlo permutations, two-sided, equal-tail permutation *P* value) using the MKinfer package (version 1.1, https://cran.r-project.org/package=MKinfer). Permutation *P* values (*P*_perm_) are set to a minimum value of 10^−5^, limited by the number of permutations (that is, minimum value of *P*_perm_ = 1 / number of permutations). To investigate interaction effects between the factors age (between-subjects factor, levels: younger, older) and layer (within-subjects factor, levels: outer, middle, inner) as well as among the factors age, layer and body part (within-subjects factor, levels: thumb, index, middle, ring, little finger or levels: hand, face), we calculated mixed-effects permutation ANOVAs (type III) using the ‘Rd_kheradPajouh_renaud’ method^87^ for random effects models as implemented in the permuco package (version 1.1.2) written for R (version 4.2.2, R Core Team, 2022). The number of permutations was set to 100,000. For comparability reasons, we report results of both parametric mixed-effects ANOVAs (using Greenhouse–Geisser-corrected degrees of freedom and *P* values in case of sphericity violations) and non-parametric permutation mixed-effects ANOVAs. Generalized eta squared (*η*_G_^2^) was calculated as an effect size estimator for parametric ANOVAs.

To follow-up significant interaction effects, we calculated Welch *t*-tests using bootstrapped samples to estimate confidence intervals and *P* values (as implemented in the MKinfer package (version 1.1))^88^, accounting for non-normality in underlying conditions. For comparability, we report both results—that is, with and without bootstrapping.

To regress out the effect of map size on layer-specific structural values, we calculated group-wise linear regression models using the lme 4 package (version 1.1.33) while controlling for the effect of individuals (random intercept model). Intercepts were re-added to the resulting residuals before they were taken forward for between-finger and hand–face comparisons. Bayesian independent-sample *t*-tests were used to compare cortical thickness between younger and older adults using two-tailed tests; the Bayes factors are specified as BF_10_ (see below for detailed explanation on Bayesian analyses).

For cohort 2, the JASP software package (version 0.17.1, JASP Team, 2023) was used to calculate Bayesian independent-sample *t*-tests for each stimulation condition on percent signal change and pRF size (*σ*) to compare younger with older adults. Shapiro–Wilk tests were used to test on data normality. Student *t*-tests were used when data were normally distributed, and Mann–Whitney tests with five chains of 1,000 iterations were used when data were not normally distributed. The choice of priors (Cauchy, scale = 0.707) and the Markov chain Monte Carlo settings were the default as implemented in JASP (for all Bayesian tests)^89^. For pRF size and activation comparisons between younger and older adults, one-tailed tests were used to test for the alternative hypothesis *σ*_younger_ < *σ*_older_, as previous studies found both enlarged pRF sizes and greater activation in older adults compared with younger adults^2,3^. As reduced co-activated inhibition has been evidenced with aging^2^, the alternative hypothesis was set as *σ*_older_ < *σ*_younger_ for pRF size difference between the double finger condition and the average of single finger conditions and %_older_ < %_younger_ for percent signal changes difference between double finger condition and the sum of single finger conditions. The Bayesian factors are specified as BF_+0_ for one-tailed tests. Following Lee and Wagenmakers^90^, we interpret the Bayes factor in support of H_1_ (BF_10_) in the following way: BF_10_ = 1–3 anecdotal evidence, BF_10_ = 3–10 moderate evidence, BF_10_ = 10–30 strong evidence, BF_10_ = 30–100 very strong evidence and BF_10_ > 100 extreme evidence; in support of H_0_: BF_10_ = 0.33–1 anecdotal evidence, BF_10_ = 0.1–0.33 moderate evidence, BF_10_ = 0.03–0.1 strong evidence, BF_10_ = 0.01–0.03 very strong evidence and BF_10_ < 0.01 extreme evidence.

Behavioral measures of cohort 1 (tactile detection thresholds, 2PD thresholds and 2PD sensitivity, finger discrimination sensitivity and precision grip accuracy) and functional outcome variables (percent signal change and network centrality) were compared between age groups using independent-sample random permutation Welch *t*-tests (100,000 Monte Carlo permutations, two-sided, equal-tail permutation *P* value) as implemented in the MKinfer package (version 1.1) for R (version 4.2.2, R Core Team, 2022).

Tactile detection thresholds and 2PD sensitivity of the right index finger, precision grip accuracy (that is, sensorimotor integration) involving both the right thumb and the right index finger as well as percent signal change (that is, responsivity) and network centrality (that is, eigenvector centrality) of the contralateral index finger representation were correlated with layer-specific qT1 values of the contralateral index finger representation. Correlation analyses were performed using Kendall’s tau correlation coefficient. Uncorrected results are reported. Before calculating correlations, the data were partly transformed (using the reciprocal of tactile detection thresholds and qT1 values), so that, in the final scatter plots, higher values always indicate better performance in behavior, higher responsivity and more connectivity in fMRI markers as well as higher substance concentration in structural MRI markers. Regression lines in the scatter plots (Fig. 6) were generated based on robust linear Theil–Sen regression estimates^43^, because the estimation of the regression coefficients is based on Kendall’s tau^44^. Cutoffs for the interpretation of Kendall’s tau correlation coefficients were approximated with the following equation, where *r* denotes the Pearson correlation coefficient^45^:

$${\tau}=2/{\uppi \; {\mathrm {arcsin}}}(r)$$

To derive the cutoff values, we applied the equation to a previously stratified convention for the Pearson correlation coefficient^46^.

For cohort 2, behavioral statistics included data of 21 healthy younger adults and 17 healthy older adults. Non-parametric independent-sample Mann–Whitney *U*-tests were used to compare texture roughness and hand dexterity measures between age groups. Effect sizes are given by the rank biserial correlation.

The functional signal central peak at layer IV of the index finger and double finger (index and middle finger together) representation was correlated with the texture roughness discrimination accuracy, the number of holes filled in the Grooved Pegboard Test and the number of pairs completed in the Small Motor Test for the index finger and the double finger condition, respectively. Correlation analyses were performed using Kendall’s tau correlation coefficient. Uncorrected results are reported.

### Animal experiments

Calcium imaging experiments were performed in younger adult mice (2–6 months; *n* = 8; two females, six males) and older adult mice (12–20 months; *n* = 8; four females, four males (ages chosen for equivalent ranges to the human cohorts 1 and 2; see also Wang et al.^91^)) of a transgenic line expressing a genetically encoded calcium indicator (GCaMP6f; C57BL/6J-Tg (Thy1-GCaMP6f) GP5.5Dkim/J; RRID: IMSR_JAX:024276). Mice were housed in individually ventilated cages (Techniplast, Green Line system) under controlled conditions (22 ± 2 °C, 55% ± 10% humidity, 12-hour light/dark cycle, with lights on at 6:00) with food and water available ad libitum. Histological analysis was performed in 12 of these mice in relation to the expression of PV^+^ neurons and in an additional 26 mice in relation to the expression of PV^+^ neurons, MBP expression as an indication of myelination and ionized calcium-binding adaptor molecule 1 (Iba1) expression as a marker for microglia (younger adult mice (*n* = 11; six females, five males, 2–6 months), older adult mice (*n* = 7; four females, three males; 12–20 months) and mice in old age (*n* = 8; three females, five males; +24 months)). All experiments were performed with reference to the National Institutes of Health Guide for the Care and Use of Laboratory animals (2011)^92^ and in accordance with the European Communities Council Directive (2010/63/EU) and approved by local authorities of Sachsen‐Anhalt/Germany (42502‐2‐1479 DZNE).

#### Mice cranial window

For cranial window implantation, mice were anesthetized with isoflurane (4% for induction and 2% maintenance). Eye cream was applied to protect the eyes (Bepanthen; Bayer), and analgesics and anti-inflammatory drugs were injected subcutaneously (carprofen 5 mg kg^−1^ and dexamethasone 2 mg kg^−1^). After shaving and disinfection of the scalp, a section of skin was removed. A craniotomy was then made over the left S1 barrel field, stereotaxically targeting the D1–D3 barrel fields (from bregma: AP −1.22 mm; ML 3 mm^93^). A 3-mm-diameter glass coverslip was then placed on top of the craniotomy and fixed with glue (Gel Control; Loctite). A custom-built headplate was implanted on the exposed skull with glue and sealed with dental acrylic (Paladur; Heraeus Kulzer).

#### Mice two-photon calcium imaging

Two-photon calcium imaging was performed using an 8-kHz resonant scanning microscope (HyperScope; Scientifica) with an Ultrafast Laser (InSight X3 Dual output laser; Spectra-Physics; <120-fs pulse width, 80-MHz repetition rate) tuned to 940 nm. Images were acquired at 30 Hz (using a ×16 objective, 0.8 NA, Nikon; tilted 10° from vertical for S1 barrel field imaging with ScanImage software (Vidrio Technologies)). Imaging of layer II/III S1 barrel field, stereotaxically targeted to the D1–D3 barrels and spanning a 500–700-µm FoV, was performed at cortical depths between 150 μm and 250 μm and layer V imaging between 420 μm and 550 µm; each FoV was imaged under three whisker stimulation conditions. The size of the imaging FoV (500–700 µm) potentially spanned approximately two neighboring barrel fields including dividing septa within the stereotaxically targeted cranial window. Note that the imaging FoV was delineated based on the optimal imaging parameters to maximize the number of neurons but without precise spatial receptive field mapping of the individual barrel fields. For all imaging sessions, mice were awake, head restrained and placed on an air-suspended polystyrene 20-cm ball (Jet Ball system; PhenoSys GmbH; sampling frequency of 30 Hz). The sampling frequency of the calcium imaging was resampled to precisely match the sampling frequency of the optical encoders, sensory stimulation, 30-Hz camera recordings (ImagingSouce, DMK22BUC03; side view of the face region) and behavioral readouts. Whisker stimulation was performed by an implemented air puff system (PhenoSys GmbH). Animals were in the dark for all trials. Signals from the calcium imaging and whisker pad tracking-estimations (see below) from the video tracking (DeepLabCut^94^) were aligned to the behavioral data file. This was done by downsampling and interpolating, ensuring that the aligned datasets had the same total number of frames, with an overall sampling frequency of approximately 30 Hz.

#### Mice whisker stimulation

All three stimulation conditions consisted of randomized air puffs directed to the right-side whiskers and two-photon imaging performed in the left barrel field. Two FoVs were imaged for each mouse under each whisker stimulus condition—one in the outer layers (layer II/III) and one in the inner layers (layer V). First, all whiskers were stimulated (all-whisker condition); after trimming the whiskers except for only two whiskers remaining on the right whisker pad (two of D1–D3 whiskers), the second session was performed (double stimulation condition; W1 + W2), and, after trimming one of the remaining two whiskers, the last session of stimulation was performed (single stimulation condition; W1). The stimulation protocol consisted of approximately 30 air puffs (200-ms duration) delivered at randomized ISIs (range, 6–20 seconds) for each FoV flanked by 2-minute imaging of spontaneous activity (10-minute total per FoV). Frame-by-frame whisker pad tracking-estimations were conducted on the video recordings captured during the session. The side view of the animal’s face was analyzed using DeepLabCut^94^. In brief, we trained a model for pose estimation on the side view videos to track the whisker pad. The pose estimation tracking was subsequently used to identify frames in which the animal was moving its whisker pad, with whisker movement defined on a frame-by-frame basis as periods with an instantaneous speed ≥0.5 cm s^−1^, 0.25-Hz low-pass filtered speed ≥0.5 cm s^−1^ and an average speed ≥0.5 cm s^−1^ over a 1-second window centered at this point in time. Any intermovement interval shorter than 500 ms was also labeled as movement.

#### Mice histology

Animals were deeply anesthetized with ketamine (20 mg per 100 g of body weight, intraperitoneally) and xylazine (1 mg per 100 g of body weight, intraperitoneally) and perfused transcardially with 20 ml of 0.1 M PBS (pH 7.4), followed by 200 ml of 4% paraformaldehyde. The brains were extracted, post-fixed overnight in 4% paraformaldehyde at 4 °C and then cryoprotected in 30% sucrose in PBS for 48 hours. Brains were cut into 50-μm-thick coronal sections (CryoStar NX70; Thermo Fisher Scientific) and collected in PBS (free floating). For immunohistochemistry, serial series of floating sections were then blocked in normal donkey serum (10% and 0.4% Triton in PBS) for 1 hour and incubated in primary antibodies overnight at 4 °C to visualize PV (monoclonal mouse anti-parvalbumin, 1:4,000, Swant, PV 235, RRID: AB_10000343; MBP, monoclonal mouse anti-myelin basic protein, 1:500, Santa Cruz Biotechnology, RRID:AB_10655672; Iba1, rabbit recombinant monoclonal anti-Iba1 antibody, 1:2,000, Abcam, RRID: AB_2636859). After rinsing in PBS, sections were incubated for 2 hours with donkey anti-mouse Cy3 (1:200, Jackson ImmunoResearch Labs, cat. no. 715-165-150, RRID: AB_2340813) or for the Iba1 staining donkey anti-rabbit Cy3 (1:200, Jackson ImmunoResearch Labs, RRID: AB_233800). Finally, sections were rinsed again in PBS, mounted on gelatin-coated slides and coverslipped with mounting media containing DAPI counterstain (VECTASHIELD). High-resolution images were captured using an epifluorescence slide scanner microscope (Axioscan 7; Zeiss) under a ×10 objective and merged (resulting in an approximately 15,000 × 11,000-pixel image per coronal section).

#### Mice data analysis

Two-photon calcium imaging datasets (30 Hz) were motion corrected, and cell detection and signal extraction were performed using Suite2p^95^. To calculate the change in fluorescence (Δ*F*/*F*), the pixel intensity is averaged within each ROI, representing the change in intensity for a single neuron, to create a raw fluorescence time series *F*(t). Baseline fluorescence *F*_0_ was computed for each neuron by taking the 5th percentile of the smoothed *F*(t) (1-Hz low-pass, zero-phase, 60th-order FIR filter), and the change in fluorescence relative to baseline (Δ*F*/*F*_0_) was calculated (*F*(t) − *F*_0_/*F*_0_). We used non-negative matrix factorization to remove neuropil contamination as implemented in FISSA^96^. All further analyses were performed using custom-written scripts in MATLAB. Histological analysis was performed on three representative sections of a 1-mm medio-lateral extent for each mouse brain across the entire cortical depth. Images were aligned to the cortical depth matching the surface, layer IV and the ventral white matter border as demarcated by DAPI. DAPI staining was also used to demarcate the cortical layers in the aligned images, namely outer layers, supragranular (I/II/III); middle layers, granular (IV); and inner layers, infragranular (V/VII) of the barrel cortex (using Fiji, ImageJ software)^97^. Cells were then automatically counted using the open-source software Cellpose^98^ with the nuclei model (DAPI) and the Cyto model (PV neurons or Iba1^+^ microglia) or the raw intensity summed across each row of pixels along the axis perpendicular to the cortical surface (MBP expression). For PV^+^ and Iba1^+^ counts, cell density was then calculated by normalizing to the area for the given demarcated cortical depths for each layer and across the given anterior posterior sections, and the percentage of all cells was calculated by normalizing to the corresponding total cell counts (DAPI nuclei).

### Reporting summary

Further information on research design is available in the Nature Portfolio Reporting Summary linked to this article.

## Online content

Any methods, additional references, Nature Portfolio reporting summaries, source data, extended data, supplementary information, acknowledgements, peer review information; details of author contributions and competing interests; and statements of data and code availability are available at 10.1038/s41593-025-02013-1.

## Supplementary information

Supplementary Figs. 1–8, Tables 1–14 and References.

Reporting Summary

Supplementary Data 1 Source data for Supplementary Information.

## Source data

Source Data Figs. 1–6 and Table 1 Statistical source data.

Source Data Extended Data Figs. 1–3 and Extended Data Tables 1–4 Statistical source data.

## Figures and Tables

**Extended Data Table 1:**
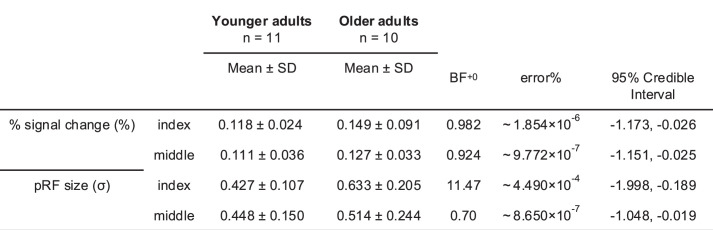
Age group comparison of percent signal change and pRF size of index finger and middle finger SI representations

Bayesian independent-sample *t*-tests were performed on younger (*n* = 11) and older (*n* = 10) adults. The alternative hypothesis H_1_ is specified as young < old, and the null hypothesis H_0_ is specified as no difference between younger and older adults.

Source data

**Extended Data Table 2:**
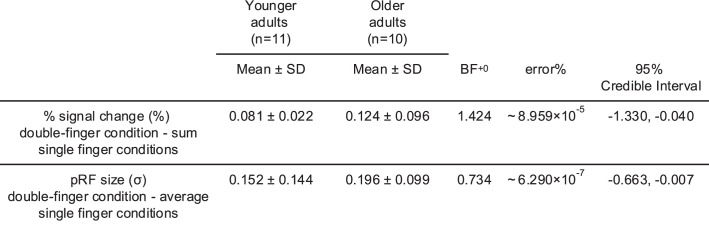
Age group comparison of percent signal change and pRF size of index and middle finger SI representations calculated as difference between double finger and single finger stimulation

Bayesian independent-sample *t*-tests were performed on younger (*n* = 11) and older (*n* = 10) adults. For percent signal change, the difference between double finger stimulation and the sum of single finger stimulation was calculated; the alternative hypothesis H_1_ is specified as %_older_ < %_younger_. For pRF size (*σ*), the difference between double finger stimulation and the average of single finger stimulation was calculated. The alternative hypothesis H_1_ is specified as *σ*_older_ < *σ*_younger_, and the null hypothesis H_0_ indicates no difference between younger and older adults on the pRF size difference and percent signal change difference.

Source data

**Extended Data Table 3:**
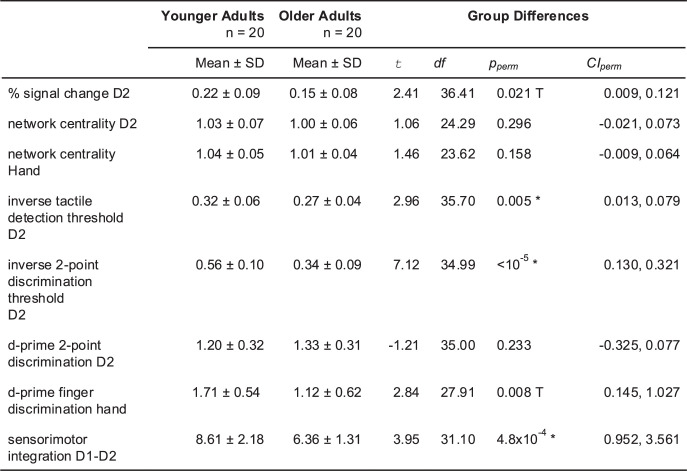
Comparison of functional map features and behavioral markers between age groups

Network centrality (based on ECM) given in a.u.; *d*-prime 2-point discrimination (based on 2PD task) and *d*-prime finger discrimination (based on finger discrimination task, averaged across all fingers) given in s.d. units; inverse tactile detection threshold given in 1 / (log_10_ 0.1 mg); inverse 2PD threshold given in 1 / mm; and sensorimotor integration (based on precision grip task) given in seconds. To facilitate the interpretation of the correlations, we ensured that higher values always indicate better performance; hence, tactile detection and 2PD thresholds were reversed. For percent signal change and network centrality, the reported body part (D1, thumb; D2, index finger, hand) indicates the localizer in area 3b from which values were extracted. We report group means (mean) and s.d.; independent-sample random permutation Welch *t*-tests were calculated to investigate group differences (*t*, test statistic; *df*, degrees of freedom; *P*_perm_, Monte Carlo permutation *P* value; CI_perm_, 95% Monte Carlo permutation confidence interval; number of permutations = 100,000; minimum value of *P*_perm_ = 1 / number of permutations). Significant differences with Bonferroni-corrected threshold of *P* < 0.006 are marked by ‘*’; trends above Bonferroni-corrected threshold are marked by ‘T’.

Source data

**Extended Data Table 4:**
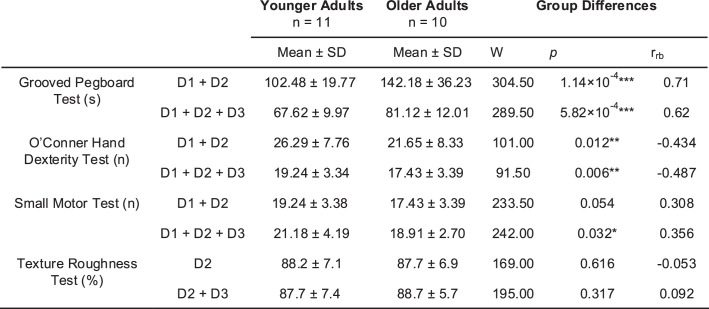
Non-parametric Wilcoxon signed-rank tests comparing measures of hand dexterity between younger (*n* = 21) and older (*n* = 17) adults using different finger(s)

(1) Time in seconds used to complete the Grooved Pegboard Test; (2) number of holes (*n*) filled within the given time interval in the O’Connor Dexterity Test; (3) number of pairs completed (*n*) within the given time interval in the Small Motor Test; and (4) accuracies on distinguishing texture in the Texture Roughness Test. D1, thumb; D2, index finger; D3, middle finger. Significant effects with *P* < 0.01 are marked by ‘*’, with *P* < 0.001 marked by ‘**’ and *P* < 0.0001 marked by ‘***’.

Source data

**Extended Data Table 5:**
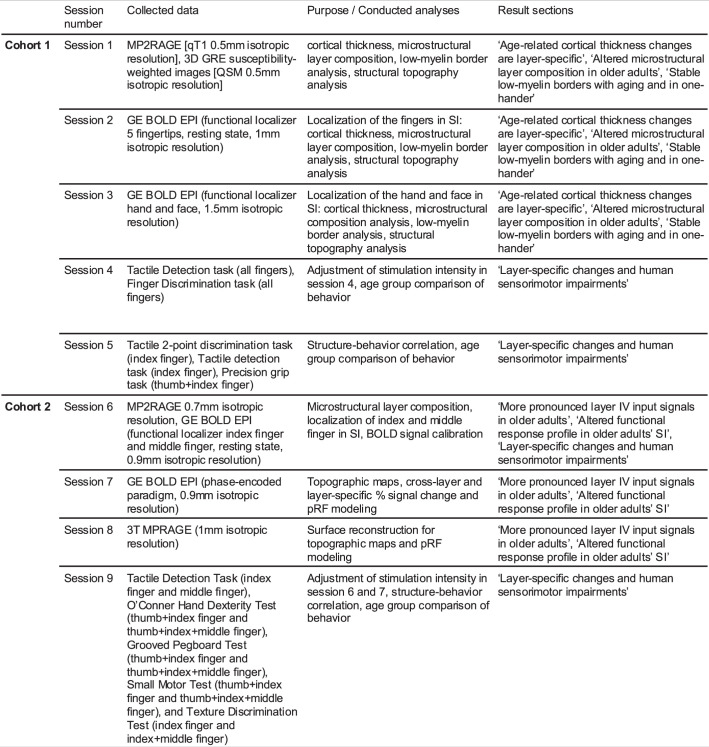
Overview of collected human 7T MRI data and its use for conducted analyses

Detailed information of the collected data of each session and the corresponding analyses in the Methods and Results sections.

Source data

## Data Availability

Owing to data protection policies, raw MRI data from human studies are available upon reasonable request; requirements are a formal data-sharing agreement and the need to submit a formal project outline. Any additional information required to reanalyze the data reported in this paper is available upon reasonable request. Source data used to make all figures are available as Source data files for main figures and extended data figures. Source data are provided with this paper.

## Code availability

Code used for human MRI data processing is available at https://github.com/PengLiu1120/cortical_layer_7T.git.
