# Supplementary material for: Layer-specific changes in sensory cortex across the lifespan in mice and humans
Source: Nat Neurosci. 2025 Aug 11;28(9):1978–89. doi: 10.1038/s41593-025-02013-1 (PMC12411234; doi:10.1038/s41593-025-02013-1)
Supplement: Supplementary file 1 — Supplementary Figs. 1–8, Tables 1–14 and References. [file 41593_2025_2013_MOESM1_ESM.pdf]

nature neuroscience

Supplementary information https://doi.org/10.1038/s41593-025-02013-1

Layer-specific changes in sensory cortex across the lifespan in mice and humans

In the format provided by the authors and unedited

Supplementary Information

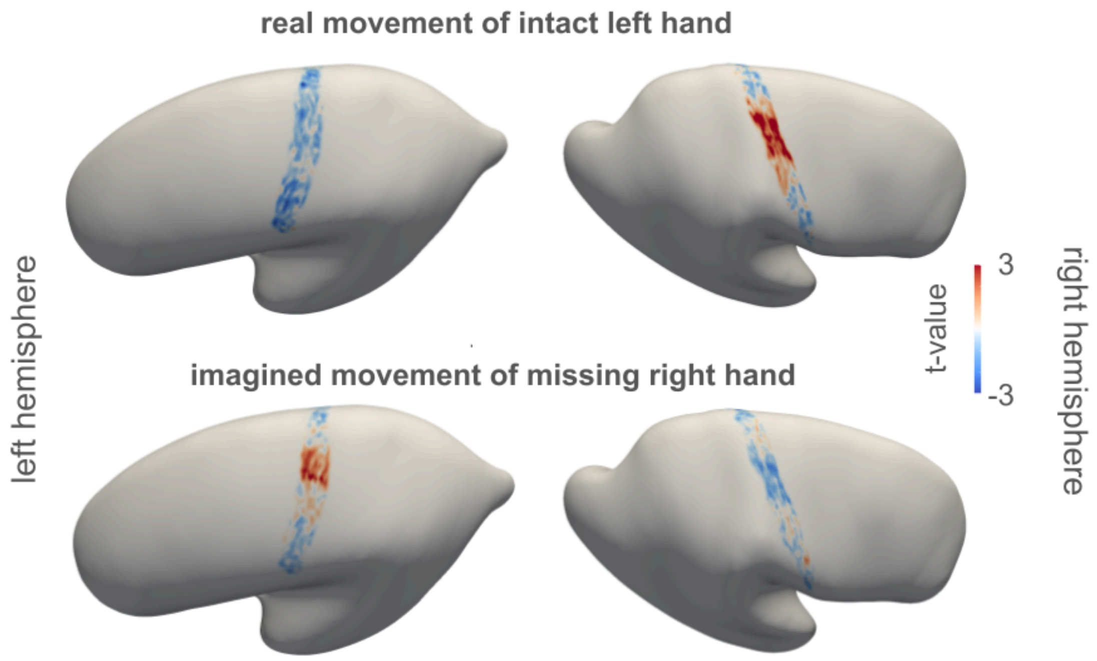

Supplementary Figure 1. Functional localizers of the hand area in area 3b of a healthy adult (male, age=52 years) with congenital arm loss on the right side. Similar to younger and older adults of cohort 1 (session 3), the participant with congenital arm loss underwent a functional localizer paradigm during 7T MRI scanning (see methods section: 'Functional localizer: hand and face'). Specifically, the participant was also instructed to move the left or right foot, the tongue or the intact left hand, while being instructed to imagine moving the missing right hand (other than cohort 1 who moved the right hand). Real movement of the intact left hand induced strong activation in area 3b of the contralateral right hemisphere, whereas imagined movement of the missing right hand induced strong activation in area 3b of the contralateral left hemisphere. Critically, the cluster of highest t-values overlaps an area where we, based on anatomical landmarks[1–3], know the hand area to be located.

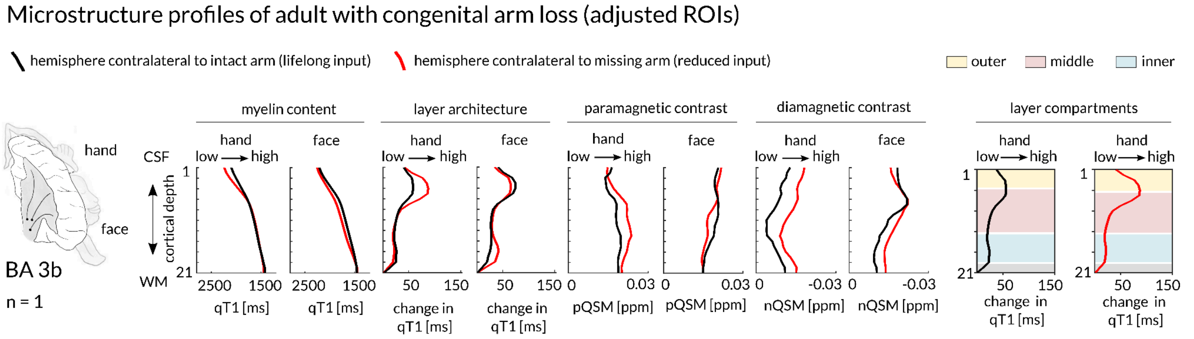

	myelin hand	myelin face	layer hand	layer face	para hand	para face	dia hand	dia face	compartments hand	compartments hand
#vertices included (black)	1652	1672	1652	1672	1558	1164	94	508	1652	—
#vertices inlcuded (red)	1661	1687	1661	1687	1337	938	324	749	—	1661

Supplementary Figure 2. **Microstructural profiles of the SI hand and face areas for the participant with congenital arm loss after controlling for map size**. Larger differences between ROI sizes contralateral and ipsilateral to the missing arm for pQSM and nQSM values are due to differences in the number of positive and negative vertices per hemisphere (because a vertex can either be positive or negative). qT1 values are given in milliseconds (ms), nQSM and pQSM values are given in part per million (ppm). Three anatomically-relevant layer compartments were extracted based on localizing maxima and minima of the first derivative of raw qT1 values. For qT1 and nQSM, lower values indicate higher substance concentration.

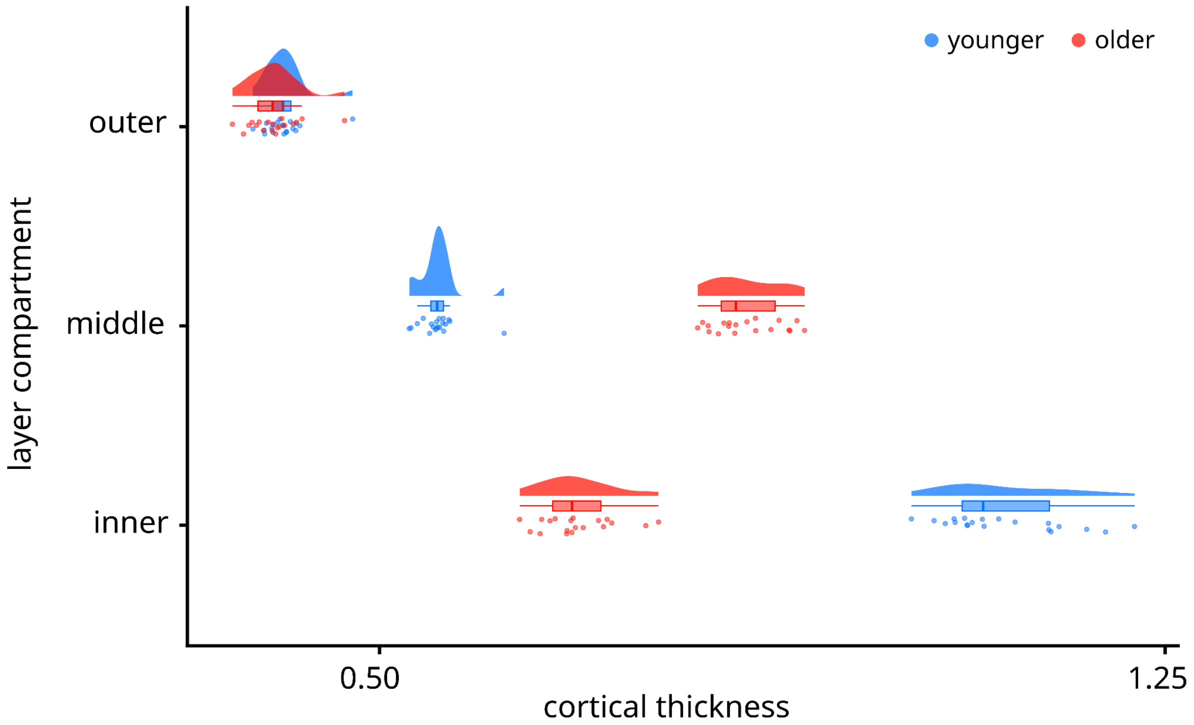

Supplementary Figure 3. Distribution of Cortical Thickness in Individual Layers of Human SI. Individual data shown as colored dots: n=20 younger adults in blue, n=19 older adults in red. Box plots are drawn within the interquartile range (box), medians are shown as vertical lines, whiskers connect the minimum and the maximum with the lower and the upper quartiles.

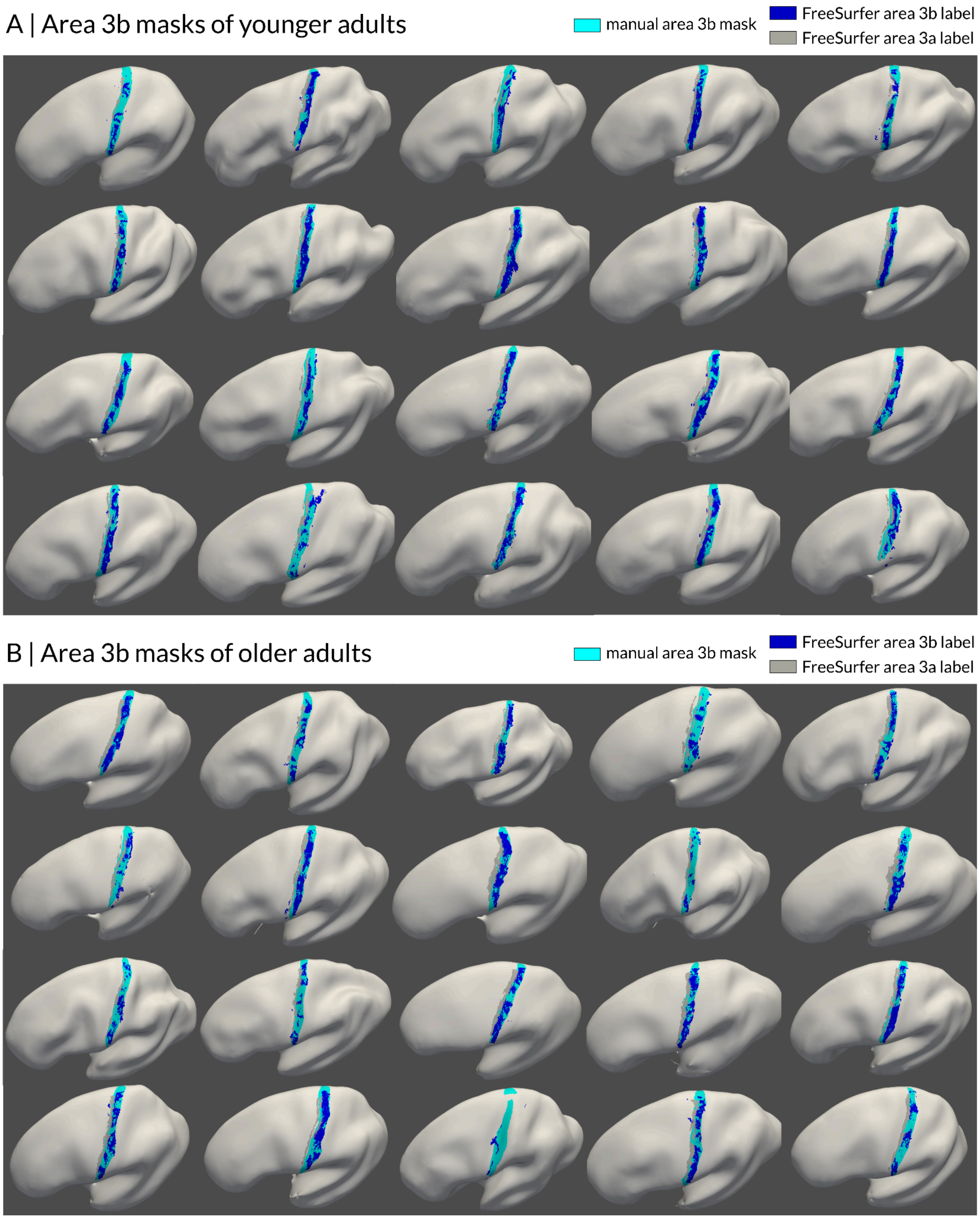

Supplementary Figure 4. Individual BA 3b masks in reference to co-registered Freesurfer labels. (A) Shown are manual area 3b masks of n=20 younger adults of cohort 1 (which were used to delineate the region of interest) on individual inflated cortical surfaces together with co-registered Freesurfer labels of area 3b and area 3a. **(B)** Manual area 3b masks of n=20 older adults of cohort 1 together with co-registered Freesurfer labels of area 3b and area 3a.

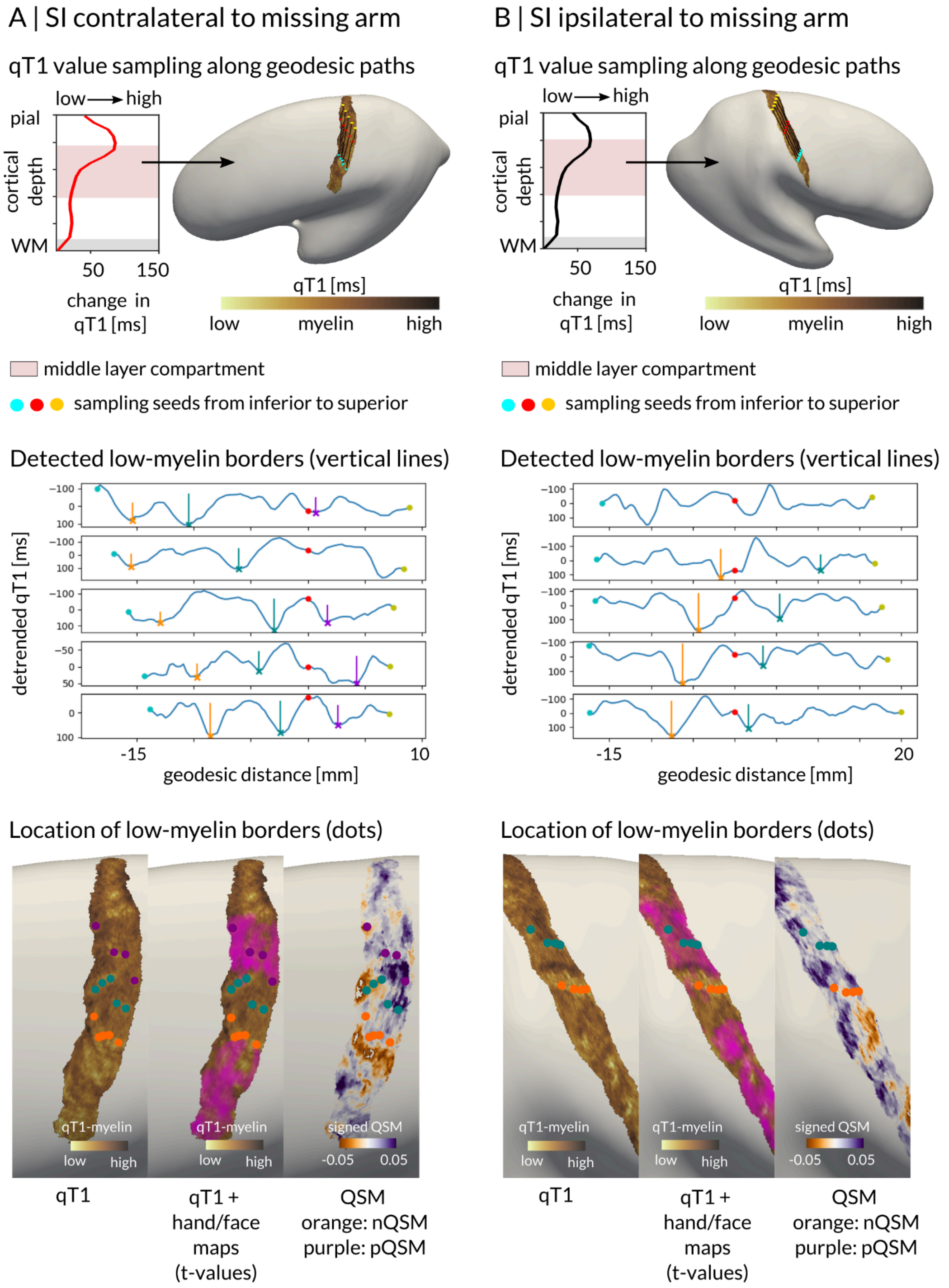

Supplementary Figure 5. Low-myelin borders in SI in the participant with congenital arm loss using an automated detection approach. (A) Detected low-myelin borders in SI (analyses focused on area 3b) contralateral to the missing arm. Analyses were based on qT1 values extracted from the middle layer compartment presumably encompassing layer IV, sampled from inferior to superior (approximately connecting the upper face representation with the superior border of the hand representation) and from anterior to posterior (along multiple geodesic paths). Additional seeds were placed along the activation peak (maximum t-value) of the hand representation (anchor to calculate geodesic distances). Detected low myelin borders in the detrended qT1 signal (vertical colored lines) in BA 3b were back-projected to cortical surfaces (colored dots in enlarged surface plots) and are shown together with different contrasts (from left to right): middle qT1 map (lighter areas indicate lower myelin content), middle qT1 map together with hand and face activation maps (t-values) localized by imagined movements of the missing right hand and real movements of the tongue, see **Supplementary Figure 1** for more details on the localizer. The quantitative susceptibility map (QSM) indicates diamagnetic (negative values, nQSM) and paramagnetic (positive values, pQSM) areas. **(B)** Same as in (A) but calculated for SI ipsilateral to the missing arm.

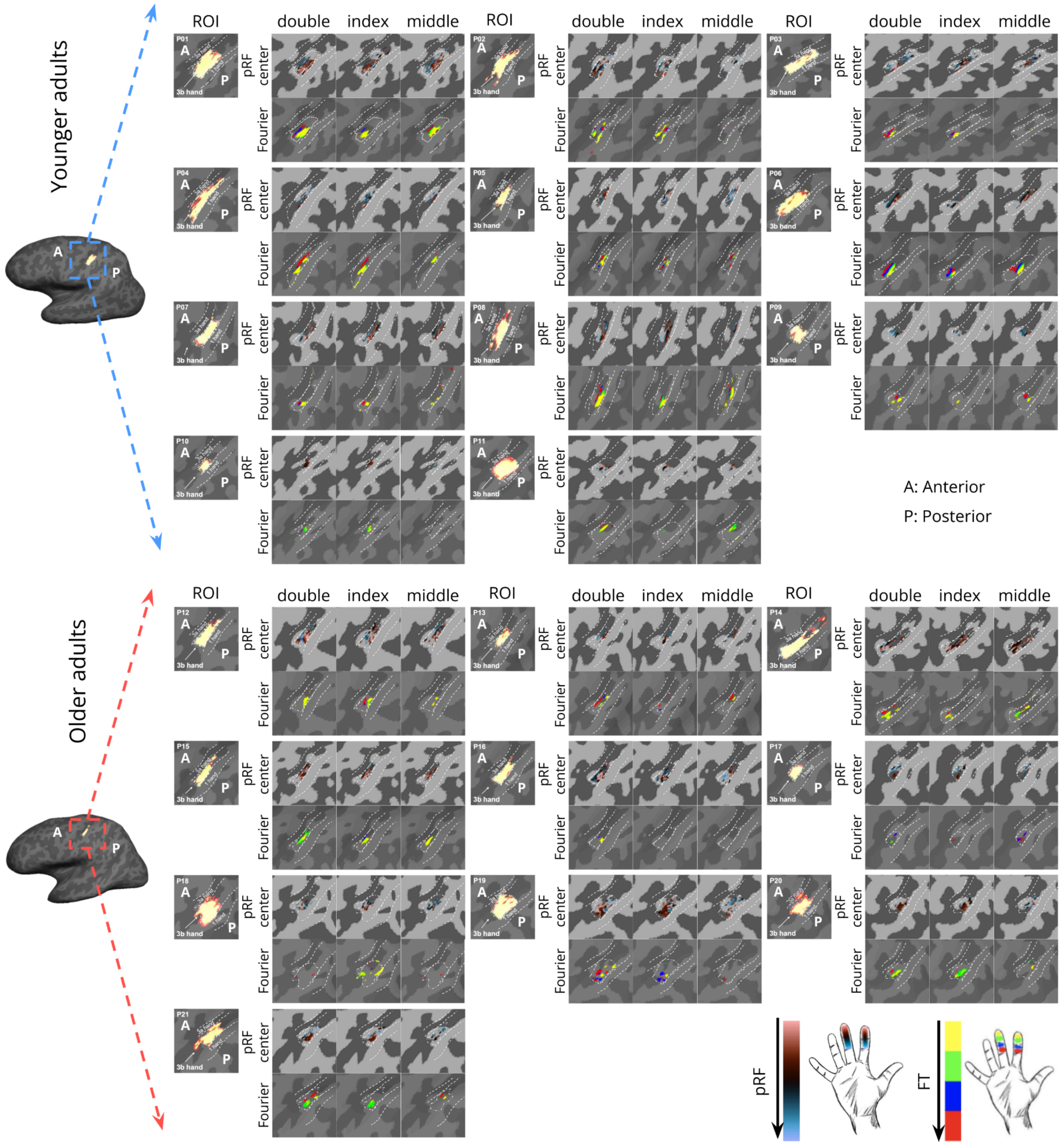

Supplemental Figure 6. Topographic maps of the index finger and the middle finger representation in area 3b of younger adults (n=11) and older adults (n=10). Each map is plotted onto the individual surface. Shown are topographic maps extracted using population receptive field (pRF) modeling (first rows in each panel) and Fourier-based mapping (second rows in each panel). ROI = Region-of-interest (hand area of contralateral area 3b).

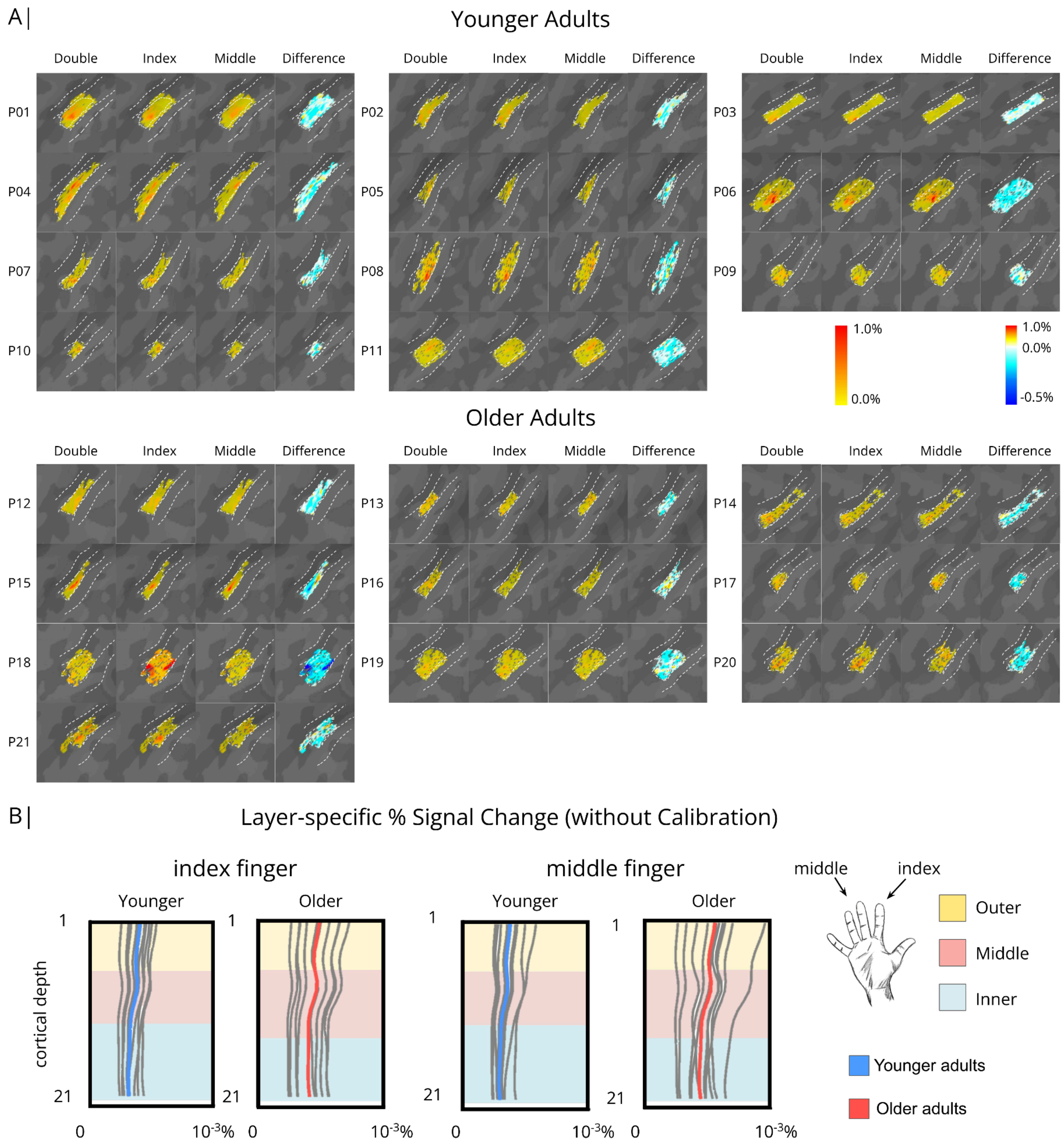

Supplementary Figure 7. Mesoscale functional architecture of finger representations in area 3b. A. % signal change map for double finger condition (index and middle finger), index finger condition, middle finger condition, and the inhibition maps (Difference=index finger condition+middle finger condition-double finger condition) during coactivation for younger (n=11) and older (n=10) adults. Each map was plotted onto the individual surface. **B.** Layer-specific %signal change of index finger and middle finger representations in SI extracted at different cortical depths for younger and older adults. Note that the calculation was performed on the BOLD signals before calibration.

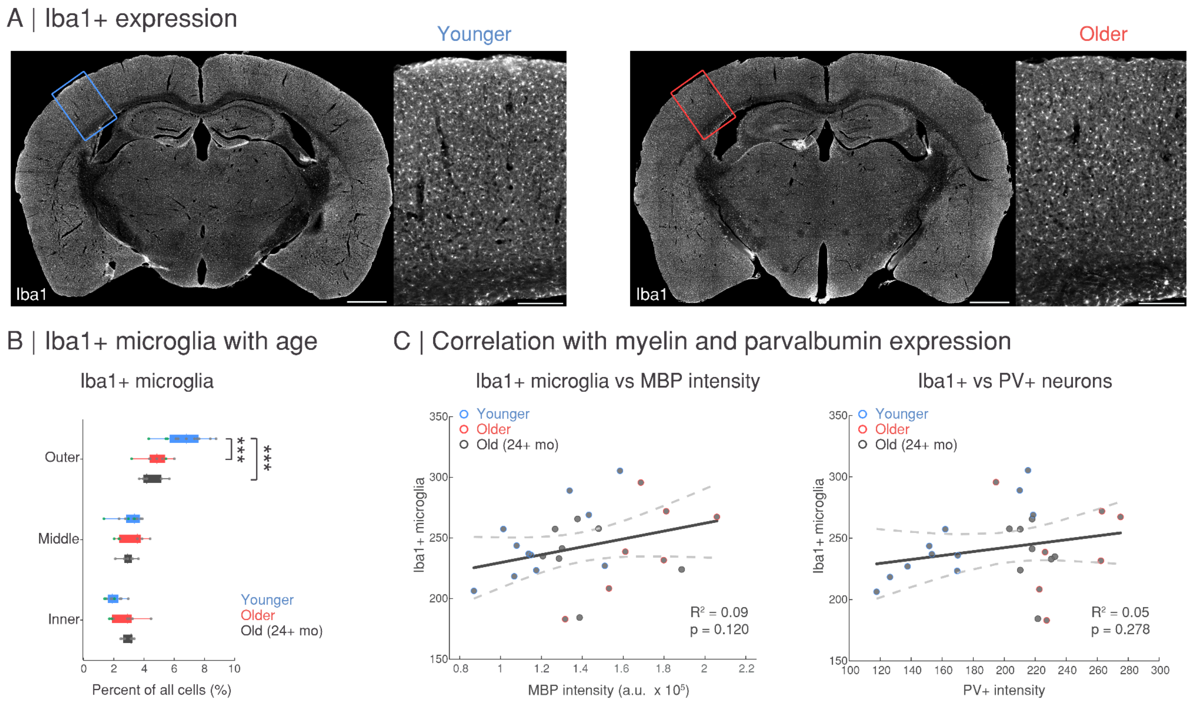

Supplementary Figure 8. Age-related changes in Iba1 immunohistochemistry as a readout of microglial density in mouse barrel cortex. (A) Representative Iba1 expression in a younger adult and older adult mouse. **(B)** The percentage of Iba1+ microglia (normalized to the DAPI total cell count) across age and outer (layer II/III), middle (layer IV) and inner (layer V/VI) cortical layers from the somatosensory barrel cortex. Significant effect of layer ($F_{(2,69)}$=78.25, *p*<0.001) but not age ($F_{(2,69)}$=2.85, *p*=0.064), and a significant interaction ($F_{(4,69)}$=10.80, *p*<0.001, two-way mixed-effects ANOVA, asterisk indicates significance level of **p*<0.05, ***p*<0.01, ****p*<0.001, values from Tukey-Kramer corrections). Data from mice with cranial window implantations (5 total, 3 younger and 2 older adult mice) are indicated as green dots. Box plots drawn within the interquartile range (box), medians shown as vertical lines, whiskers connect the minimum and the maximum with the lower and the upper quartiles. **(C)** Linear regression R^2 and 95% confidence intervals (dashed lines) for the number of Iba1+ microglia (average count per sample, summed across cortical depth) and intensity of myelin basic protein (MBP; average intensity per sample, summed across cortical depth) expression (left; R^2=0.09, *p*=0.120) or the number of parvalbumin (PV+) expressing cells (average count per sample, summed across cortical depth; right; R^2=0.05, *p*=0.278) per animal (n=26 mice: younger adult mice [n=11, 2-6 months], older adult mice [n=7, 12-20 months], and mice in old age [n=8, +24 months]). Scale bars in **(A)**, 1mm on coronal section and 250µm inset.

Supplementary Table 1. Bayesian independent-sample t-tests of layer-specific cortical thickness differences of the SI hand area between younger and older adults. Shown are total and layer-specific (outer, middle, inner) mean cortical thickness values (Mean) and standard deviations (SD) in millimeters for the SI hand area. Bayesian independent-sample t-tests were performed on younger (n=20) and older (n=19) adults. The alternative hypothesis H_1 is specified as $thickness_{young} \neq thickness_{old}$ for total, outer, middle and inner, and the null hypothesis H_0 is specified as no difference between younger and older adults on total cortical thickness and the thickness of each cortical layer compartment.

	Age				
	Younger adults (n=20)	Older adults (n=19)			
	Mean ± SD	Mean ± SD	BF_{10}	error%	95% Credible Interval
total	2.06 ± 0.07	1.94 ± 0.08	1383.792	7.538×10^{-9}	0.41, 1.82
outer	0.41 ± 0.02	0.40 ± 0.02	0.635	0.005	0.04, 1.05
middle	0.56 ± 0.02	0.85 ± 0.03	8.931×10^{21}	5.925×10^{-28}	-2.21, -0.68
inner	1.10 ± 0.06	0.69 ± 0.04	2.045×10^{26}	4.522×10^{-24}	0.72, 2.25

Supplementary Table 2. Layer-specific cortical thickness of the SI hand area without outlier removal. Shown are total and layer-specific (outer, middle, inner) mean cortical thickness values (Mean) and standard deviations (SD) in millimeters for the SI hand area. Independent-samples random permutation Welch t-tests were calculated to investigate group differences (t=test statistic, df=degrees of freedom, p_{perm}=Monte-Carlo permutation p-value, CI_{perm}=95% Monte-Carlo permutation confidence interval, number of permutations=100000, minimum value of p_{perm}=1/number of permutations). Significant differences with Bonferroni-corrected p<0.0125 (correcting for 4 tests) are marked by *.

hand area	All n = 40	**Younger** n = 20	**Older** n = 20	**Group Differences**			
	Mean ± SD	Mean ± SD	Mean ± SD	t	*df*	p_{perm}	CI_{perm}
total	2.01 ± 0.10	2.06 ± 0.07	1.95 ± 0.10	3.8	33.4	5.4×10^{-4} *	0.04, 0.17
outer	0.46 ± 0.06	0.41 ± 0.02	0.51 ± 0.04	-10.1	26.8	$< 10^{-5}$ *	-0.14, -0.07
middle	0.65 ± 0.10	0.56 ± 0.02	0.75 ± 0.04	-18.7	26.0	$< 10^{-5}$ *	-0.26, -0.13
inner	0.89 ± 0.21	1.10 ± 0.06	0.69 ± 0.04	26.6	31.0	$< 10^{-5}$ *	0.27, 0.54

Supplementary Table 3. Cortical thickness comparison for the SI (i.e. BA 3b) hand and face areas localized by motor movements in relation to anatomically-relevant layer compartments. Given are layer-specific (outer, middle, inner) mean cortical thickness values (Mean) and standard deviations (SD) in millimeters for hand and face regions of younger and older adults (localized by movements of corresponding body parts). Independent-samples random permutation Welch t-tests were calculated to investigate group differences (t=test statistic, df=degrees of freedom, p_{perm}=Monte-Carlo permutation p-value, CI_{perm}=95% Monte-Carlo permutation confidence interval, number of permutations=100000, minimum value of p_{perm}=1/number of permutations). Significant differences with Bonferroni-corrected p<0.006 (correcting for 8 tests) are marked by *.

	All n = 34	**Younger** n = 16	**Older** n = 18	**Group Differences**			
	Mean ± SD	Mean ± SD	Mean ± SD	*t*	*df*	p_{perm}	CI_{perm}
**hand total**	2.01 ± 0.10	2.06 ± 0.05	1.96 ± 0.11	3.24	25.3	0.003 *	0.03, 0.16
hand outer	0.47 ± 0.06	0.42 ± 0.02	0.52 ± 0.04	-10.1	22.1	$<10^{-5}$*	-0.14, -0.06
hand middle	0.67 ± 0.10	0.56 ± 0.01	0.75 ± 0.04	-18.6	20.5	$<10^{-5}$ *	-0.26, -0.12
hand inner	0.87 ± 0.20	1.08 ± 0.05	0.69 ± 0.05	24.4	31.4	$<10^{-5}$ *	0.25, 0.52
**face total**	2.13 ± 0.16	2.22 ± 0.10	2.05 ± 0.16	3.74	29.1	6.0^{-4} *	0.06, 0.28
face outer	0.51 ± 0.06	0.46 ± 0.04	0.56 ± 0.05	-6.95	31.1	$<10^{-5}$ *	-0.14, 0.05
face middle	0.70 ± 0.11	0.60 ± 0.03	0.78 ± 0.08	-9.46	23.0	$<10^{-5}$ *	-0.25, -0.11
face inner	0.92 ± 0.23	1.16 ± 0.05	0.71 ± 0.07	22.01	31.8	$<10^{-5}$ *	0.29, 0.60

Supplementary Table 4. Thickness comparison for the SI (i.e. BA 3b) hand area (localized by pRF center location maps) using equal layer compartments (layer definition of younger adults). Given are layer-specific (outer, middle, inner) mean cortical thickness values (Mean) and standard deviations (SD) in millimeters for the BA 3b hand region (localized by vibro-tactile stimulation to the five fingertips of the right hand). Independent-samples random permutation Welch t-tests were calculated to investigate group differences (t=test statistic, df=degrees of freedom, p_{perm}=Monte-Carlo permutation p-value, CI_{perm}=95% Monte-Carlo permutation confidence interval, number of permutations=100000, minimum value of p_{perm}=1/number of permutations). Significant differences with Bonferroni-corrected p<0.016 (correcting for 3 tests) are marked by *. Trends above Bonferroni-corrected threshold are marked by a T.

	All n = 40	**Younger** n = 20	**Older** n = 20	**Group Differences**			
	Mean ± SD	Mean ± SD	Mean ± SD	t	*df*	p_{perm}	CI_{perm}
hand outer	0.41 ± 0.03	0.41 ± 0.02	0.40 ± 0.03	0.5	30.8	0.640	-0.01, 0.02
hand middle	0.55 ± 0.03	0.56 ± 0.02	0.55 ± 0.04	0.7	28.0	0.538	-0.01, 0.02
hand inner	1.05 ± 0.07	1.10 ± 0.06	1.00 ± 0.05	5.7	36.7	$<10^{-5}$ *	0.05, 0.14

Supplementary table 5. Microstructural layer composition of SI hand and face areas of a participant with congenital arm loss after controlling for map size. Cortical fields (i.e. hand and face regions) were localized by motor movements or imagery of motor movements (for the missing limb condition). Given are layer-specific (outer, middle, inner) qT1 (in milliseconds), nQSM (in parts per million), and pQSM (in parts per million) values. Lower qT1 and nQSM values indicate higher substance concentration. For microstructure profiles plotted along the dimension of cortical depth see **Table 1 - supplemental table 3**.

	qT1	nQSM	pQSM	qT1	nQSM	pQSM
	contralateral to missing limb			ipsilateral to missing limb		
hand outer	2107.4	-0.017	0.015	2081.1	-0.012	0.016
hand middle	1678.5	-0.013	0.021	1705.2	-0.007	0.017
hand inner	1499.5	-0.011	0.022	1508.2	-0.008	0.019
face outer	2142.4	-0.017	0.021	2140.1	-0.019	0.020
face middle	1794.3	-0.019	0.017	1750.3	-0.018	0.020
face inner	1559.9	-0.014	0.015	1527.2	-0.010	0.016

Supplementary Table 6. **Comparison of myelin, calcium, iron and mineralization content between age groups, fingers and layer compartments.** Permutation mixed-effects ANOVAs (“type III” sum of square) with between-subjects factor age (levels: younger, older) and within-subjects factors layer (levels: inner, middle, outer) and finger (D1, D2, D3, D4, D5) were performed on residual qT1 (myelin), nQSM (calcium), pQSM (iron) and aQSM (mineralization) values after controlling for finger map size (F=test statistic, df=degrees of freedom, p=parametric p-value, df_{GG}=Greenhouse-Geiser corrected degrees of freedom, p_{GG}=Greenhouse-Geiser corrected parametric p-value, η_G^2=effect size estimator generalized Eta-squared, p_{perm}=permutation p-value using the method by Kherad-Pajouh and Renaud[4] for non-spherical data with 100000 permutations). Please note that the minimum value of p_{perm} is given by 1/number of permutations. For qT1 analysis n=2 participants (1 older and 1 younger), for nQSM n=12 participants (5 younger, 7 older), for pQSM n=4 participants (2 younger, 2 older), for aQSM n=2 participants (1 younger, 1 older) were excluded because of missing finger maps. Significant effects with Bonferroni-corrected p<0.0125 (correcting for 4 ANOVAs) are marked by *. Trends above Bonferroni-corrected threshold are marked by a T.

effect	*F*	*DFn, DFd*	*p*	*DFn_{GG}, DFd_{GG}*	*p_{GG}*	*η_G^2*	*p_{perm}*
**qT1 (n=38)**							
age	3.3	1, 36	0.077 T	1.00, 36.00	0.077 T	0.049	0.076 T
layer	286.1	2,72	5.5×10^{-35} *	1.05, 37.87	1.5×10^{-19} *	0.690	$<10^{-5}$ *
finger	2.3	4, 144	0.059 T	3.16, 113.64	0.075 T	0.006	0.057 T
age x layer	5.1	2, 72	0.009 *	1.05, 37.87	0.028 T	0.038	0.008 *
age x finger	0.1	4, 144	0.976	3.16, 113.64	0.955	3.1^{-4}	0.976
layer x finger	1.2	8, 288	0.295	3.57, 128.47	0.311	0.002	0.298
age x layer x finger	0.9	8, 288	0.541	3.57, 128.47	0.473	0.002	0.545
**nQSM (n=22)**							
age	4.0	1, 20	0.060 T	1.00, 20.00	0.060 T	0.039	0.059 T
layer	7.1	1, 40	0.002 *	1.44, 28.71	0.007 *	0.070	0.002 *
finger	0.2	4, 80	0.930	4.00, 80.00	0.930	0.003	0.929
age x layer	5.8	2, 40	0.006 *	1.44, 28.71	0.014 T	0.058	0.006 *
age x finger	0.4	4, 80	0.806	4.00, 80.00	0.806	0.006	0.806
layer x finger	1.0	8, 160	0.462	4.58, 91.59	0.436	0.014	0.462
age x layer x finger	0.6	8, 160	0.795	4.58, 91.59	0.702	0.008	0.797
**pQSM (n=30)**							
age	35.3	1, 28	2.1×10^{-6} *	1.00, 28.00	2.1×10^{-6} *	0.262	$<10^{-5}$ *
layer	11.2	2, 56	8.2×10^{-5} *	2.00, 56.00	8.2×10^{-5} *	0.046	9.0×10^{-5} *
finger	1.5	4, 112	0.205	2.36, 66.19	0.227	0.019	0.204
age x layer	1.4	2, 56	0.257	2.00, 56.00	0.257	0.006	0.258
age x finger	1.2	4, 112	0.331	2.36, 66.19	0.325	0.015	0.334

layer x finger	0.4	8, 224	0.892	4.45, 124.52	0.794	0.004	0.892
age x layer x finger	1.0	8, 224	0.463	4.45, 124.52	0.435	0.008	0.467
**aQSM (n=32)**							
age	43.8	1, 30	2.5×10^{-7} *	1.00, 30.00	2.5×10^{-7} *	0.291	$<10^{-5}$ *
layer	8.2	2, 60	7.3×10^{-4} *	2.00, 60.00	7.3×10^{-4} *	0.040	8.7×10^{-4} *
finger	1.5	4, 120	0.194	2.74, 82.18	0.213	0.015	0.195
age x layer	0.4	2, 60	0.649	2.00, 60.00	0.649	0.002	0.650
age x finger	1.1	4, 120	0.342	2.74, 82.18	0.336	0.011	0.344
layer x finger	1.4	8, 240	0.179	4.53, 135.92	0.217	0.012	0.178
age x layer x finger	0.3	8, 240	0.962	4.53, 135.92	0.892	0.003	0.962

Supplementary Table 7. Comparison of qT1, nQSM, pQSM and aQSM values between age groups, fingers and layer compartments using the layer definition of younger adults for both age groups. Permutation mixed-effects ANOVAs ("type III" sum of square) with between-subjects factor age (levels: younger, older) and within-subjects factors layer (levels: inner, middle, outer) and finger (D1, D2, D3, D4, D5) were performed on residual qT1 (myelin), nQSM (calcium), pQSM (iron) and aQSM (mineralization) values after controlling for finger map size (F=test statistic, df=degrees of freedom, p=parametric p-value, df_{GG}=Greenhouse-Geiser corrected degrees of freedom, p_{GG}=Greenhouse-Geiser corrected parametric p-value, η_G^2=effect size estimator generalized Eta-squared, p_{perm}=permutation p-value using the method by Kherad-Pajouh and Renaud[4] for non-spherical data with 100000 permutations. Please note that the minimum value of p_{perm} is given by 1/number of permutations. For qT1 analysis n=2 participants (1 older, 1 younger), for nQSM n=13 participants (5 younger, 8 older), for pQSM n=4 participants (2 younger, 2 older), for aQSM n=2 participants (1 younger, 1 older) were excluded because of missing finger maps. Significant effects with Bonferroni-corrected p<0.0125 (correcting for 4 ANOVAs) are marked by *. Trends above Bonferroni-corrected threshold are marked by a T.

effect	*F*	*DFn, DFd*	*p*	*DFn_{GG}, DFd_{GG}*	p_{GG}	η_G^2	p_{perm}
**qT1 (n=38)**							
age	0.1	1, 36	0.765	1.00, 36.00	0.765	0.002	0.771
layer	277.4	2, 72	1.5×10^{-34} *	1.06, 38.31	1.6×10^{-19} *	0.654	$<10^{-5}$ *
finger	2.4	4, 144	0.056 T	3.06, 110.07	0.074 T	0.006	0.055 T
age x layer	2.8	2, 72	0.070 T	1.06, 38.31	0.102	0.019	0.069 T
age x finger	0.2	4, 144	0.940	3.06, 110.07	0.902	0.001	0.940
layer x finger	1.2	8, 288	0.275	3.70, 133.30	0.297	0.002	0.276
age x layer x finger	0.7	8, 288	0.648	3.70, 133.30	0.551	0.001	0.650
**nQSM (n=21)**							
age	11.2	1, 19	0.003 *	1.00, 19.00	0.003 *	0.091	0.003 *
layer	15.1	2, 38	1.5×10^{-5} *	1.36, 25.81	0.002 *	0.147	3.0×10^{-5} *
finger	0.3	4, 76	0.878	4.00, 76.00	0.878	0.005	0.878
age x layer	2.0	2, 38	0.146	1.36, 25.81	0.163	0.023	0.147
age x finger	1.0	4, 76	0.419	4.00, 76.00	0.419	0.016	0.421
layer x finger	1.6	8, 152	0.123	4.10, 77.84	0.176	0.025	0.124
age x layer x finger	0.8	8, 152	0.645	4.10, 77.84	0.562	0.012	0.645
**pQSM (n=30)**							
age	37.9	1, 28	1.2×10^{-6} *	1.00, 28.00	1.2×10^{-6} *	0.270	$<10^{-5}$ *
layer	5.9	2, 56	0.005 *	2.00, 56.00	0.005 *	0.022	0.005 *
finger	1.4	4, 112	0.227	2.44, 68.41	0.243	0.020	0.228
age x layer	0.1	2, 56	0.917	2.00, 56.00	0.917	3.3×10^{-4}	0.917
age x finger	1.1	4, 112	0.354	2.44, 68.41	0.343	0.015	0.356

layer x finger	0.5	8, 224	0.876	4.41, 123.50	0.775	0.004	0.878
age x layer x finger	1.0	8, 224	0.421	4.41, 123.50	0.404	0.008	0.423
**aQSM (n=32)**							
age	45.6	1, 30	1.7×10^{-7} *	1.00, 30.00	1.7×10^{-7} *	0.310	$<10^{-5}$ *
layer	6.2	2, 60	0.004 *	2.00, 60.00	0.004 *	0.026	0.004 *
finger	1.2	4, 120	0.298	2.87, 86.00	0.300	0.014	0.300
age x layer	0.5	2, 60	0.596	2.00, 60.00	0.596	0.002	0.598
age x finger	0.8	4, 120	0.528	2.87, 86.00	0.492	0.009	0.527
layer x finger	1.8	8, 240	0.083 T	4.71, 141.23	0.127	0.014	0.082 T
age x layer x finger	0.4	8, 240	0.918	4.71, 141.23	0.836	0.003	0.919

Supplementary Table 8. **Comparison of qT1, nQSM, pQSM and aQSM values between age groups, body parts (hand, face) and layer compartments.** Permutation mixed-effects ANOVAs ("type III" sum of square) with between-subjects factor age (levels: younger, older) and within-subjects factors layer (levels: inner, middle, outer) and body part (hand, face) were performed on residual qT1 (myelin), nQSM (calcium), pQSM (iron) and aQSM (mineralization) values after controlling for body part map size (F=test statistic, df=degrees of freedom, p=parametric p-value, df_{GG}=Greenhouse-Geiser corrected degrees of freedom, p_{GG}=Greenhouse-Geiser corrected parametric p-value, η_G^2=effect size estimator generalized Eta-squared, p_{perm}=permutation p-value using the method by Kherad-Pajouh and Renaud[4] for non-spherical data with 100000 permutations. Please note that the minimum value of p_{perm} is given by 1/number of permutations. Significant effects with Bonferroni-corrected p<0.0125 (correcting for 4 ANOVAs) are marked by *. Trends above Bonferroni-corrected threshold are marked by a T.

effect	*F*	*DFn, DFd*	*p*	*DFn_{GG}, DFd_{GG}*	*p_{GG}*	η_G^2	*p_{perm}*
**qT1 (n=34)**							
age	1.1	1, 32	0.312	1.00, 32.00	0.312	0.022	0.316
layer	416.8	2,64	2.0×10^{-37} *	1.04, 33.25	1.3×10^{-20} *	0.778	$<10^{-5}$ *
body part	0.1	1, 32	0.735	1.00, 32.00	0.735	1.2×10^{-4}	0.737
age x layer	15.3	2, 64	3.7×10^{-6} *	1.04, 33.25	3.7×10^{-4} *	0.114	$<10^{-5}$ *
age x body part	1.5	1, 32	0.231	1.00, 32.00	0.231	0.001	0.232
layer x body part	5.3	2, 64	0.007 *	1.21, 38.84	0.021 T	0.002	0.007 *
age x layer x body part	2.1	2, 64	0.132	1.21, 38.84	0.153	0.001	0.131
**nQSM (n=30)**							
age	32.8	1, 28	3.7×10^{-6} *	1.00, 28.00	3.7×10^{-6} *	0.313	$<10^{-5}$ *
layer	36.9	2, 56	6.0×10^{-11} *	1.23, 34.50	1.4×10^{-7} *	0.315	$<10^{-5}$ *
body part	2.7	1, 28	0.113	1.00, 28.00	0.113	0.008	0.112
age x layer	2.3	2, 56	0.107	1.23, 34.5	0.131	0.028	0.106
age x body part	8.5	1, 28	0.007 *	1.00, 28.0	0.007 *	0.026	0.006 *
layer x body part	4.4	2, 56	0.016 T	1.34, 37.4	0.032 T	0.027	0.017 T
age x layer x body part	3.1	2, 56	0.053 T	1.34, 37.4	0.075 T	0.019	0.052 T
**pQSM (n=30)**							
age	45.3	1, 28	2.6×10^{-7} *	1.00, 28.00	2.6×10^{-7} *	0.478	$<10^{-5}$ *
layer	32.0	2, 56	5.4×10^{-10} *	1.11, 31.01	1.7×10^{-6} *	0.229	$<10^{-5}$ *
body part	18.3	1, 28	2.0×10^{-4} *	1.00, 28.00	2.0×10^{-4} *	0.053	1.4×10^{-4} *
age x layer	16.2	2, 56	2.8×10^{-6} *	1.11, 31.01	2.3×10^{-4} *	0.131	$<10^{-5}$ *
age x body part	0.01	1, 28	0.939	1.00, 28.00	0.939	1.8×10^{-5}	0.939

layer x body part	7.6	2, 56	0.001 *	1.29, 36.14	0.006 *	0.023	0.001 *
age x layer x body part	0.8	2, 56	0.440	1.29, 36.14	0.396	0.003	0.446
**aQSM (n=30)**							
age	83.2	1, 28	7.1×10^{-10} *	1.00, 28.00	7.1×10^{-10} *	0.622	$<10^{-5}$ *
layer	97.3	2, 56	6.0^{-19} *	1.27, 35.46	7.7^{-13} *	0.463	$<10^{-5}$ *
body part	14.6	1, 28	6.8×10^{-4} *	1.00, 28.00	6.8×10^{-4} *	0.046	4.7×10^{-4} *
age x layer	21.7	2, 56	1.1×10^{-7} *	1.27, 35.46	1.3×10^{-5} *	0.161	$<10^{-5}$ *
age x body part	0.2	1, 28	0.657	1.00, 28.00	0.657	0.001	0.659
layer x body part	10.5	2, 56	1.4×10^{-4} *	1.42, 39.63	8.4×10^{-4} *	0.038	9.0×10^{-5} *
age x layer x body part	4.3	2, 56	0.018 T	1.42, 39.63	0.032 T	0.016	0.017 T

Supplementary Table 9. **Post-hoc comparisons to follow up significant main effects, significant interaction effects and trends on qT1, nQSM, pQSM and aQSM related to cortical fields (hand, face) and layer compartments.** Bootstrap Welch paired-sample t-tests were performed on qT1 (myelin) (n=16 younger adults, n=18 older adults), nQSM (calcium) (n=14 younger adults, n=16 older adults), pQSM (iron) (n=14 younger adults, n=16 older adults) and aQSM (mineralization) (n=14 younger adults, n=16 older adults) values to follow up significant main effects of body part (tested differences: hand-face) and interactions between body part and layer (tested differences: hand outer - face outer, hand middle - face middle, hand inner - face inner), Bootstrap Welch two-sample t-tests were performed to follow up significant interaction effects between age (younger, older) and body part (tested differences: younger hand - older hand, younger face - older face) as well as between age, body part and layer (y=younger, ol=older; o=outer, m=middle, i=inner). Results are given as mean difference values (Mean) and standard errors (SE) in milliseconds (for qT1) or in ppm (for QSM-based estimates), test statistic (t), degrees of freedom (df), p-value (p), bootstrapped p-value (p_{boot}), 95% confidence interval (CI), bootstrapped 95% confidence interval (CI_{boot}). Number of bootstrap samples=100000, minimum value of p_{boot}=1/number of bootstrap samples. Significant effects with Bonferroni-corrected p<0.002 (correcting for 25 post-hoc tests) are marked by *. Trends above Bonferroni-corrected threshold are marked by a T.

comparison	Mean ± SE	*t*	*df*	*p*	p_{boot}	*CI*	CI_{boot}
**qT1 (n=34)**							
face outer > hand outer	5.4 ± 12.3	0.4	33	0.665	0.652	-19.7, 30.5	-17.9, 30.0
face middle > hand middle	16.1 ± 7.4	2.2	33	0.036 T	0.037 T	1.1, 31.1	1.7, 30.2
face inner < hand inner	-11.8 ± 7.5	-1.6	33	0.128	0.114	-27.1, 3.6	-26.5, 2.5
**nQSM (n=30)**							
younger face > older face	0.004 ± $6.3x10^{-4}$	6.8	20.7	$9.9x10^{-4}$ *	$<10^{-5}$ *	$3.0x10^{-3}$, $5.7x10^{-3}$	$3.1x10^{-3}$, $5.5x10^{-3}$
younger hand > older hand	0.003 ± $6.5x10^{-4}$	4.1	19.8	$6.0x10^{-4}$ *	$5.6x10^{-4}$ *	$1.2x10^{-3}$, $4.0x10^{-3}$	$1.4x10^{-3}$, $3.9x10^{-3}$
y face o > ol face o	0.005 ± $1.0x10^{-3}$	5.2	23.9	$2.7x10^{-5}$ *	$6.0x10^{-5}$ *	$3.2x10^{-3}$, $7.4x10^{-3}$	$3.3x10^{-3}$, $7.2x10^{-3}$
y face m > ol face m	0.005 ± $8.1x10^{-4}$	6.7	21.0	$1.3x10^{-6}$ *	$<10^{-5}$ *	$3.7x10^{-3}$, $7.1x10^{-3}$	$3.9x10^{-3}$, $6.9x10^{-3}$
y face i > o face i	0.002 ± $7.0x10^{-4}$	3.2	28.0	0.003 T	0.004 T	$8.3x10^{-4}$, $3.7x10^{-3}$	$9.4x10^{-4}$, $3.6x10^{-3}$
y hand o > ol hand o	0.002 ± $1.2x10^{-3}$	1.6	17.5	0.129	0.102	$-6.2x10^{-4}$, $4.4x10^{-3}$	$-2.8x10^{-4}$, $4.3x10^{-3}$
y hand m > ol hand m	0.004 ± $8.2x10^{-4}$	4.3	19.8	$3.4x10^{-4}$ *	$1.8x10^{-4}$ *	$1.8x10^{-3}$, $5.3x10^{-3}$	$2.0x10^{-3}$, $5.1x10^{-3}$
y hand i > ol hand i	0.002 ± $9.3x10^{-4}$	2.6	23.3	0.016 T	0.007 T	$4.9x10^{-4}$, $4.3x10^{-3}$	$7.3x10^{-4}$, $4.2x10^{-3}$
**pQSM (n=30)**							
face < hand	-0.001 ± $3.4x10^{-4}$	-4.4	29	$1.5x10^{-4}$ *	$1.5x10^{-4}$ *	$-2.2x10^{-3}$, $-7.9x10^{-4}$	$-2.1x10^{-3}$, $-8.1x10^{-4}$
face outer <	$-8.8x10^{-5}$ ±	-0.1	29	0.900	0.915	$-1.5x10^{-3}$,	$-1.4x10^{-3}$,

hand outer	6.9×10^{-4}					1.3×10^{-3}	1.3×10^{-3}
face middle < hand middle	-0.002 ± 2.9×10^{-4}	-7.3	29	4.8×10^{-8} *	$<10^{-5}$ *	-2.7×10^{-3}, -1.5×10^{-3}	-2.6×10^{-3}, -1.5×10^{-3}
face inner < hand inner	-0.002 ± 4.0×10^{-4}	-5.6	29	4.3×10^{-6} *	$<10^{-5}$ *	-3.1×10^{-3}, -1.4×10^{-3}	-3.1×10^{-3}, -1.5×10^{-3}
**aQSM (n=30)**							
face < hand	-0.001 ± 2.6×10^{-4}	-3.8	29	6.0×10^{-4} *	2.4×10^{-4} *	-1.5×10^{-3}, -4.7×10^{-4}	-1.5×10^{-3}, -5.0×10^{-4}
face outer > hand outer	3.4×10^{-4} ± 4.8×10^{-4}	0.7	29	0.490	0.491	-6.5×10^{-4}, 1.3×10^{-3}	-5.9×10^{-4}, 1.3×10^{-3}
face middle < hand middle	-1.6×10^{-3} ± 3.1×10^{-4}	-5.0	29	2.3×10^{-5} *	$<10^{-5}$ *	-2.2×10^{-3}, -9.4×10^{-4}	-2.2×10^{-3}, -1.0×10^{-3}
face inner < hand inner	-1.7×10^{-3} ± 3.6×10^{-4}	-4.8	29	3.9×10^{-5} *	$<10^{-5}$ *	-2.5×10^{-3}, -1.0×10^{-3}	-2.4×10^{-3}, -1.1×10^{-3}
y face o < ol face o	-9.2×10^{-3} ± 9.3×10^{-4}	-9.9	23.1	8.0×10^{-10} *	$<10^{-5}$ *	-1.1×10^{-2}, -7.3×10^{-3}	-1.1×10^{-2}, -7.6×10^{-3}
y face m < ol face m	-5.5×10^{-3} ± 6.5×10^{-4}	-8.5	22.6	1.7×10^{-8} *	$<10^{-5}$ *	-6.9×10^{-3}, -4.2×10^{-3}	-6.7×10^{-3}, -4.3×10^{-3}
y face i < ol face i	-3.1×10^{-3} ± 5.5×10^{-4}	-5.6	25.6	7.3×10^{-6} *	$<10^{-5}$ *	-4.2×10^{-3}, -1.9×10^{-3}	-4.1×10^{-3}, -2.0×10^{-3}
y hand o < ol hand o	-7.0×10^{-3} ± 1.1×10^{-3}	-6.5	18.2	3.6×10^{-6} *	$<10^{-5}$ *	-9.7×10^{-3}, -5.0×10^{-3}	-9.7×10^{-3}, -5.4×10^{-3}
y hand m < ol hand m	-5.9×10^{-3} ± 8.0×10^{-4}	-7.4	22.8	1.6×10^{-7} *	$<10^{-5}$ *	-7.6×10^{-3}, -4.3×10^{-3}	-7.4×10^{-3}, -4.4×10^{-3}
y hand i < ol hand i	-3.8×10^{-3} ± 7.3×10^{-4}	-5.2	21.1	3.7×10^{-5} *	$<10^{-5}$ *	-5.3×10^{-3}, -2.3×10^{-3}	-5.2×10^{-3}, -2.4×10^{-3}

Supplementary Table 10. Comparison of qT1, nQSM, pQSM and aQSM values between age groups and layer compartments. Permutation mixed-effects ANOVAs (“type III” sum of square) with between-subjects factor age (levels: younger, older) and within-subjects factor layer (levels: inner, middle, outer) were performed on qT1 (myelin), nQSM (calcium), pQSM (iron) and aQSM (mineralization) values (F=test statistic, df=degrees of freedom, p=parametric p-value, df_{GG}=Greenhouse-Geiser corrected degrees of freedom, p_{GG}=Greenhouse-Geiser corrected parametric p-value, η_G^2=effect size estimator generalized Eta-squared, p_{perm}=permutation p-value using the method by Kherad-Pajouh & Renaud[4] for non-spherical data with 100000 permutations, minimum value of p_{perm}=1/number of permutations). Significant effects with Bonferroni-corrected p < 0.0125 (correcting for 4 ANOVAs) are marked by *.

effect	*F*	*DFn, DFd*	*p*	*DFn_{GG}, DFd_{GG}*	p_{GG}	η_G^2	p_{perm}
**qT1 (n=40)**							
age	2.01	1, 38	0.164	1.00, 38.00	0.164	0.034	0.167
layer	338.01	2, 76	1.5×10^{-38} *	1.06, 40.46	1.1×10^{-21} *	0.747	$<10^{-5}$ *
age x layer	7.24	2, 76	0.001 *	1.06, 40.46	0.009 *	0.059	7.5×10^{-4} *
**nQSM (n=34)**							
age	11.01	1, 32	0.002 *	1.00, 32.00	0.002 *	0.135	0.002 *
layer	15.34	2, 64	3.6×10^{-6} *	1.16, 37.24	2.0×10^{-4} *	0.207	$<10^{-5}$ *
age x layer	0.20	2, 64	0.822	1.16, 37.24	0.698	0.003	0.824
**pQSM (n=34)**							
age	22.68	1, 32	4.0×10^{-5} *	1.00, 32.00	4.0×10^{-5} *	0.301	5.0×10^{-5} *
layer	12.76	2, 64	2.2×10^{-5} *	1.36, 43.45	2.7×10^{-4} *	0.135	3.0×10^{-5} *
age x layer	1.06	2, 64	0.351	1.36, 43.45	0.330	0.013	0.353
**aQSM (n=34)**							
age	26.70	1, 32	1.2×10^{-5} *	1.00, 32.00	1.2×10^{-5} *	0.349	2.0×10^{-5} *
layer	17.65	2, 64	7.8×10^{-7} *	1.55, 49.71	9.2×10^{-6} *	0.164	$<10^{-5}$ *
age x layer	1.16	2, 64	0.321	1.55, 49.71	0.312	0.013	0.323

Supplementary Table 11. Post-hoc comparisons to follow up significant layer-specific age differences in qT1, nQSM, pQSM and aQSM values. Bootstrap Welch paired-sample t-tests were performed on qT1 (myelin), nQSM (calcium), pQSM (iron) and aQSM (mineralization) values to follow up significant main effects of layer (tested differences: outer-middle, middle-inner), Bootstrap Welch two-sample t-tests were performed to follow up significant main effects of layer (inner, middle, outer; tested differences; outer-middle, middle-inner) and significant interaction effects between age (younger, older) and layer (tested differences: younger outer-older outer, younger middle-older middle, younger inner-older inner). Results are given as mean difference values (Mean) and standard errors (SE) in milliseconds (for qT1) or ppm (for QSM-based estimates), test statistic (t), degrees of freedom (df), p-value (p), bootstrapped p-value (p_{boot}), 95% confidence interval (CI), bootstrapped 95% confidence interval (CI_{boot}). Number of bootstrap samples=100000, minimum value of p_{boot}=1/number of bootstrap samples. Significant effects with Bonferroni-corrected p<0.0045 (correcting for 11 post-hoc tests) are marked by *.

comparison	Mean ± SE	*t*	*df*	*p*	p_{boot}	*CI*	CI_{boot}
**qT1 (n=40)**							
outer > middle	279.8 ± 22.3	12.6	39.0	$2.8x10^{-15}$ *	$<10^{-5}$ *	234.8, 324.9	240.2, 325.8
middle > inner	236.9 ± 8.6	27.6	39.0	$3.2x10^{-27}$ *	$<10^{-5}$ *	219.5, 254.2	221.6, 254.8
younger outer < older outer	-41.2 ± 61.3	-0.7	25.0	0.508	0.467	-167.3, 85.0	-164.7, 69.7
younger middle > older middle	91.2 ± 25.4	3.6	30.8	0.001 *	0.002 *	39.3, 143.1	41.6, 139.2
younger inner > older inner	88.7 ± 19.3	4.6	36.8	$4.8x10^{-5}$ *	$4.0x10^{-5}$ *	49.7, 127.8	51.6, 125.2
**nQSM (n=34)**							
outer < middle	$-2.9x10^{-3}$ ± $3.2x10^{-4}$	-9.2	33.0	$1.4x10^{-10}$ *	$<10^{-5}$ *	$-3.5x10^{-3}$, $-2.3x10^{-3}$	$-3.5x10^{-3}$, $-2.3x10^{-3}$
middle > inner	$3.4x10^{-4}$ ± $5.4x10^{-4}$	0.6	33.0	0.541	0.569	$-7.7x10^{-4}$, $1.4x10^{-3}$	$-7.5x10^{-4}$, $1.3x10^{-3}$
**pQSM (n=34)**							
outer > middle	$5.0x10^{-4}$ ± $6.3x10^{-4}$	0.8	33	0.428	0.392	$-7.7x10^{-4}$, $1.8x10^{-3}$	$-6.6x10^{-4}$, $1.8x10^{-3}$
middle > inner	$2.2x10^{-3}$ ± $3.5x10^{-4}$	6.3	33	$4.5x10^{-7}$ *	$<10^{-5}$ *	$1.5x10^{-3}$, $2.9x10^{-3}$	$1.6x10^{-3}$, $2.9x10^{-3}$
**aQSM (n=34)**							
outer > middle	$1.1x10^{-3}$ ± $4.5x10^{-4}$	2.5	33	0.019 T	0.024 T	$1.9x10^{-4}$, $2.0x10^{-3}$	$2.3x10^{-4}$, $2.0x10^{-3}$
middle > inner	$1.4x10^{-3}$ ± $3.1x10^{-4}$	4.5	33	$8.5x10^{-5}$ *	$<10^{-5}$ *	$7.7x10^{-4}$, $2.0x10^{-3}$	$8.2x10^{-4}$, $2.0x10^{-3}$

Supplementary Table 12. Comparison of qT1, nQSM, pQSM and aQSM values of the SI hand area between age groups and layer compartments using the layer definition of younger adults for both age groups. Permutation mixed-effects ANOVAs ("type III" sum of square) with between-subjects factor age (levels: younger, older) and within-subjects factor layer (levels: inner, middle, outer) were performed on qT1 (myelin) (n=20 younger adults, n=20 older adults), nQSM (calcium) (n=18 younger adults, n=16 older adults), pQSM (iron) (n=18 younger adults, n=16 older adults) and aQSM (mineralization) (n=18 younger adults, n=16 older adults) values (F=test statistic, df=degrees of freedom, p=parametric p-value, df_{GG}=Greenhouse-Geiser corrected degrees of freedom, p_{GG}=Greenhouse-Geiser corrected parametric p-value, η_G^2=effect size estimator generalized Eta-squared, p_{perm}=permutation p-value using the method by Kherad-Pajouh and Renaud[4] for non-spherical data with 100000 permutations, minimum value of p_{perm}=1/number of permutations). Significant effects with Bonferroni-corrected p<0.0125 (correcting for 4 ANOVAs) are marked by *. Trends above Bonferroni-corrected threshold are marked by a T.

effect	*F*	*DFn, DFd*	*p*	*DFn_{GG}, DFd_{GG}*	p_{GG}	η_G^2	p_{perm}
**qT1 (n=40)**							
age	0.04	1, 38	0.834	1.00, 38.00	0.834	0.001	0.837
layer	328.62	2, 76	3.9×10^{-38} *	1.08, 41.05	9.9×10^{-22} *	0.712	$<10^{-5}$ *
age x layer	4.14	2, 76	0.020 T	1.08, 41.05	0.046 T	0.030	0.016 T
**nQSM (n=34)**							
age	14.49	1, 32	6.0×10^{-4} *	1.00, 32.00	6.0×10^{-4} *	0.188	5.8×10^{-4} *
layer	18.82	2, 64	3.7×10^{-7} *	1.15, 36.76	5.6×10^{-5} *	0.224	$<10^{-5}$ *
age x layer	1.94	2, 64	0.152	1.15, 36.76	0.171	0.029	0.151
**pQSM (n=34)**							
age	27.86	1, 32	8.8×10^{-6} *	1.00, 32.00	8.8×10^{-6} *	0.347	2.0×10^{-5} *
layer	11.58	2, 64	5.1×10^{-5} *	1.28, 40.86	6.7×10^{-4} *	0.123	3.0×10^{-5} *
age x layer	0.61	2, 64	0.547	1.28, 40.86	0.610	0.007	0.549
**aQSM (n=34)**							
age	30.70	1, 32	4.1×10^{-6} *	1.00, 32.00	4.1×10^{-6} *	0.390	2.0×10^{-5} *
layer	17.10	2, 64	1.1×10^{-6} *	1.31, 42.03	4.4×10^{-5} *	0.152	$<10^{-5}$ *
age x layer	1.14	2, 64	0.325	1.31, 42.03	0.308	0.012	0.326

Supplementary Table 13. Post-hoc comparisons to follow up significant main and interaction effects of age on qT1, nQSM, pQSM and aQSM values of the SI hand area using the layer definition of younger adults for both age groups. Bootstrap Welch paired-sample t-tests were performed on qT1 (myelin) (n=20 younger adults, n=20 older adults), nQSM (calcium) (n=18 younger adults, n=16 older adults), pQSM (iron) (n=18 younger adults, n=16 older adults) and aQSM (mineralization) (n=18 younger adults, n=16 older adults) values to follow up significant main effects of layer (tested differences: outer-middle, middle-inner), Bootstrap Welch two-sample t-tests were performed to follow up significant main effects of layer (inner, middle, outer; tested differences: outer - middle, middle - inner) and significant interaction effects between age (younger, older) and layer (tested differences: younger outer - older outer, younger middle - older middle, younger inner - older inner). Results are given as mean difference values (Mean) and standard errors (SE) in milliseconds (for qT1) or in ppm (for QSM-based estimates), test statistic (t), degrees of freedom (df), p-value (p), bootstrapped p-value (p_{boot}), 95% confidence interval (CI), bootstrapped 95% confidence interval (CI_{boot}). Number of bootstrap samples=100000, minimum value of p_{boot}=1/number of bootstrap samples. Significant effects with Bonferroni-corrected p<0.0045 (correcting for 11 post-hoc tests) are marked by *. Trends above Bonferroni-corrected threshold are marked by a T.

comparison	Mean ± SE	*t*	*df*	*p*	p_{boot}	*CI*	CI_{boot}
**qT1 (n=40)**							
outer > middle	250.8 ± 17.4	14.4	39.0	3.1x10^{-17} *	<10^{-5} *	215.7, 286.0	219.5, 286.2
middle > inner	256.9 ± 12.7	20.3	39.0	2.6x10^{-22} *	<10^{-5} *	231.3, 282.5	235.6, 284.2
younger outer < older outer	-69.8 ± 63.4	-1.1	24.6	0.282	0.232	-200.5, 60.9	-196.7, 45.0
younger middle > older middle	4.7 ± 33.4	0.1	25.5	0.89	0.912	-64.0, 73.4	-61.9, 65.6
younger inner > older inner	42.2 ± 19.0	2.2	37.1	0.033 T	0.034 T	3.7, 80.7	5.7, 77.9
**nQSM (n=34)**							
outer < middle	-2.7x10^{-3} ± 2.6x10^{-4}	-10.6	33	3.8x10^{-12} *	<10^{-5} *	-3.2x10^{-3}, -2.2x10^{-3}	-3.2x10^{-3}, -2.2x10^{-3}
middle < inner	-4.1x10^{-4} ± 6.0x10^{-4}	-0.7	33	0.500	0.461	-1.6x10^{-3}, 8.1x10^{-4}	-1.6x10^{-3}, 6.9x10^{-4}
**pQSM (n=34)**							
outer > middle	6.4x10^{-4} ± 6.3x10^{-4}	1.0	33	0.322	0.282	-6.5x10^{-4}, 1.9x10^{-3}	-5.4x10^{-4}, 1.9x10^{-3}
middle > inner	2.0x10^{-3} ± 3.1x10^{-4}	6.5	33	2.3x10^{-7} *	<10^{-5} *	1.4x10^{-3}, 2.7x10^{-3}	1.4x10^{-3}, 2.6x10^{-3}
**aQSM (n=34)**							
outer > middle	1.0x10^{-3} ± 4.3x10^{-4}	2.4	33	0.023 T	0.028 T	1.5x10^{-4}, 1.9x10^{-3}	1.9x10^{-4}, 1.9x10^{-3}
middle > inner	1.4x10^{-3} ± 2.7x10^{-4}	5.3	33	8.7x10^{-6} *	<10^{-5} *	8.6x10^{-4}, 1.9x10^{-3}	9.0x10^{-4}, 1.9x10^{-3}

Supplementary Table 14. Age group comparison on central peak and signal decay at input layer IV. Bayesian independent-sample t-tests were performed on younger (n=11) and older (n=10) adults**.** The alternative hypothesis H_1 is specified as young<old, and the null hypothesis H_0 is specified as no difference between younger and older adults.

	Central peak				
	Younger adults (n=11)	Older adults (n=10)			
Condition	Mean±SD	Mean±SD	BF_{+0}	error%	95% Credible Interval
index	$1.78\times10^{-3}\pm3.548\times10^{-4}$	$2.24\times10^{-3}\pm6.445\times10^{-4}$	3.146	~ 3.333×10^{-5}	-1.454, -0.054
middle	$1.65\times10^{-3}\pm3.795\times10^{-4}$	$2.47\times10^{-3}\pm7.747\times10^{-4}$	15.533	~ 6.461×10^{-4}	-1.695, -0.089

	Signal decay				
	Younger adults (n=11)	Older adults (n=10)			
Condition	Mean±SD	Mean±SD	BF_{+0}	error%	95% Credible Interval
index	$2.32\times10^{-3}\pm4.824\times10^{-4}$	$2.91\times10^{-3}\pm8.493\times10^{-4}$	2.769	~ 2.756×10^{-5}	-1.380, -0.047
middle	$2.15\times10^{-3}\pm5.162\times10^{-4}$	$3.22\times10^{-3}\pm0.001$	13.405	~ 5.665×10^{-4}	-1.675, -0.096

References

1. Geyer, S., Schleicher, A. & Zilles, K. Areas 3a, 3b, and 1 of Human Primary Somatosensory Cortex: 1. Microstructural Organization and Interindividual Variability. *NeuroImage* **10**, 63–83 (1999).
2. Kumabe, T., Nakasato, N., Inoue, T. & Yoshimoto, T. Primary thumb sensory cortex located at the lateral shoulder of the inverted omega-shape on the axial images of the central sulcus. *Neurol. Med. Chir. (Tokyo)* **40**, 393–403 (2000).
3. Yousry, T. Localization of the motor hand area to a knob on the precentral gyrus. A new landmark. *Brain* **120**, 141–157 (1997).
4. Kherad-Pajouh, S. & Renaud, O. A general permutation approach for analyzing repeated measures ANOVA and mixed-model designs. *Stat. Pap.* **56**, 947–967 (2015).
