# Supplementary material for: Layer-specific changes in sensory cortex across the lifespan in mice and humans
Source: Nat Neurosci. 2025 Aug 11;28(9):1978–89. doi: 10.1038/s41593-025-02013-1 (PMC12411234; doi:10.1038/s41593-025-02013-1)
Supplement: Supplementary file 2 — Reporting Summary [file 41593_2025_2013_MOESM2_ESM.pdf]

nature portfolio

Corresponding author(s): Juliane Doehler

Last updated by author(s): May 16, 2025

Reporting Summary

Nature Portfolio wishes to improve the reproducibility of the work that we publish. This form provides structure for consistency and transparency in reporting. For further information on Nature Portfolio policies, see our Editorial Policies and the Editorial Policy Checklist.

Statistics

For all statistical analyses, confirm that the following items are present in the figure legend, table legend, main text, or Methods section.

n/a	Confirmed	
☐	☒	The exact sample size (*n*) for each experimental group/condition, given as a discrete number and unit of measurement
☐	☒	A statement on whether measurements were taken from distinct samples or whether the same sample was measured repeatedly
☐	☒	The statistical test(s) used AND whether they are one- or two-sided<br>*Only common tests should be described solely by name; describe more complex techniques in the Methods section.*
☐	☒	A description of all covariates tested
☐	☒	A description of any assumptions or corrections, such as tests of normality and adjustment for multiple comparisons
☐	☒	A full description of the statistical parameters including central tendency (e.g. means) or other basic estimates (e.g. regression coefficient) AND variation (e.g. standard deviation) or associated estimates of uncertainty (e.g. confidence intervals)
☐	☒	For null hypothesis testing, the test statistic (e.g. *F*, *t*, *r*) with confidence intervals, effect sizes, degrees of freedom and *P* value noted<br>*Give P values as exact values whenever suitable.*
☐	☒	For Bayesian analysis, information on the choice of priors and Markov chain Monte Carlo settings
☐	☒	For hierarchical and complex designs, identification of the appropriate level for tests and full reporting of outcomes
☐	☒	Estimates of effect sizes (e.g. Cohen's *d*, Pearson's *r*), indicating how they were calculated

Our web collection on statistics for biologists contains articles on many of the points above.

Software and code

Policy information about availability of computer code

Data collection	MATLAB R2015b(PsychToolbox), Presentation
Data analysis	MIPAV v7.3.0, CBSTools v3.0.8, ANTs v2.1.0, ITK-SNAP V3.6.0, FSL, Freesurfer v7.3.0, Csurf v0.8, MATLAB v2017b (SPM8), MATLAB v2018b (SPM12, BayespRF, SamSrf), LIPSIA v3.0, Python (Nighres, Pyvista, SciPy, PhysioNoise), Suite2p (Pachitariu et al., 2016; v0.14.0), FISSA (Keemink et al., 2018; v.1.0.0), DeepLabCut (Mathis et al., 2018; v2.3.8), ImageJ, Cellpose (Stringer et al., 2025; v3.0.10), JASP v0.17.1, R 4.2.2<br>Customized code are provided via https://github.com/PengLiu1120/cortical_layer_7T.git and https://github.com/pakanlab/Liu_et_al_NatureNeuroscience2025

For manuscripts utilizing custom algorithms or software that are central to the research but not yet described in published literature, software must be made available to editors and reviewers. We strongly encourage code deposition in a community repository (e.g. GitHub). See the Nature Portfolio guidelines for submitting code & software for further information.

Data

Policy information about availability of data

All manuscripts must include a data availability statement. This statement should provide the following information, where applicable:

- Accession codes, unique identifiers, or web links for publicly available datasets
- A description of any restrictions on data availability
- For clinical datasets or third party data, please ensure that the statement adheres to our policy

Due to data protection policies, raw MRI data from human studies are available upon request, requirements are a formal data sharing agreement and the need to submit a formal project outline. Any additional information required to reanalyze the data reported in this paper is available upon request. Source data used to make all figures are available as Source Data files for main figures and extended data figures. Source data are provided with this paper.

Research involving human participants, their data, or biological material

Policy information about studies with human participants or human data. See also policy information about sex, gender (identity/presentation), and sexual orientation and race, ethnicity and racism.

Reporting on sex and gender	Cohort 1 and cohort 2 is composed of 50% male and 50% female.<br>Sex was determined via self-report using a binary question of male/female.<br>We did not distinguish sex and gender.
Reporting on race, ethnicity, or other socially relevant groupings	We did not collect data on race, ethnicity other socially relevant groupings.
Population characteristics	We classified the participants into younger adults (age between 18 and 35) and older adults (age above 65).
Recruitment	Younger and older adults from both cohorts were recruited from the participant bank of the German Center for Neurodegenerative Diseases (DZNE) Magdeburg, Germany. The participant with congenital arm loss was recruited from the database of the Central Institute of Mental Health (CIMH) Mannheim, Germany. Participants of both cohort 1 and cohort 2 were right-handed.
Ethics oversight	The study was approved by the Ethics committee of the Otto-von-Guericke University Magdeburg.

Note that full information on the approval of the study protocol must also be provided in the manuscript.

Field-specific reporting

Please select the one below that is the best fit for your research. If you are not sure, read the appropriate sections before making your selection.

☒ Life sciences ☐ Behavioural & social sciences ☐ Ecological, evolutionary & environmental sciences

For a reference copy of the document with all sections, see nature.com/documents/nr-reporting-summary-flat.pdf

Life sciences study design

All studies must disclose on these points even when the disclosure is negative.

Sample size	The estimation of the number of human cohort 1 was motivated by previous layer-dependent 7T MRI studies using quantitative in-vivo proxies to describe the microstructural cortex architecture (Dinse et al., 2015, Kuehn et al., 2017). With a group size of 20 younger and 20 older adults, a large effect size 0.9 and a power (1-beta) of 0.8 can be detected, and is well above previously reported sample sizes. The participant for cohort 2 was motivated by our previous 7T functional MRI study (Liu et al., 2021).<br><br>Dinse, J. et al. A cytoarchitecture-driven myelin model reveals area-specific signatures in human primary and secondary areas using ultra-high resolution in-vivo brain MRI. NeuroImage 114, 71–87 (2015).<br>Kuehn, E. et al. Body Topography Parcellates Human Sensory and Motor Cortex. Cereb. Cortex 27, 3790–3805 (2017).<br>Liu, P. et al. The organizational principles of de-differentiated topographic maps in somatosensory cortex. eLife 10, e60090 (2021).
Data exclusions	Participants were excluded from the study due to severe motion artifacts in the imaging data.
Replication	Population receptive field sizes were calculated in both cohort 1 and cohort 2, and compared with our previous study (Liu et al., 2021), where we found consisted results that older adults present larger pRF sizes compared to younger adults.<br>A larger input channel at layer IV was also reproduced between cohorts, despite using different methods: cohort 1 using structural data and cohort 2 using functional data.
Randomization	The participants were allocated into two groups according to age (younger adults: age between 18 and 35 and older adults: age above 65).<br>Animals were tagged with numbers randomly and selected by sequence of animal number.

Blinding	The blinding was not possible during the data collection and analyses because age is not a factor that can be blinded.

Reporting for specific materials, systems and methods

We require information from authors about some types of materials, experimental systems and methods used in many studies. Here, indicate whether each material, system or method listed is relevant to your study. If you are not sure if a list item applies to your research, read the appropriate section before selecting a response.

Materials & experimental systems

n/a	Involved in the study
☐	☒ Antibodies
☒	☐ Eukaryotic cell lines
☒	☐ Palaeontology and archaeology
☐	☒ Animals and other organisms
☒	☐ Clinical data
☒	☐ Dual use research of concern
☒	☐ Plants

Methods

n/a	Involved in the study
☒	☐ ChIP-seq
☒	☐ Flow cytometry
☐	☒ MRI-based neuroimaging

Antibodies

Antibodies used	Antibodies were used in animal experiments for immunohistological processing. Primary antibodies used are:<br>PV, monoclonal mouse anti-parvalbumin, 1:4000, Swant, PV 235, RRID: AB_10000343;<br>MBP, monoclonal mouse anti-myelin basic protein, 1:500, Santa Cruz, RRID:AB_10655672;<br>Iba1, rabbit recombinant monoclonal anti-Iba1 antibody, 1:2000, Abcam, RRID: AB_2636859.<br><br>Secondary antibodies used are:<br>donkey anti-mouse Cy3 (1:200, Jackson ImmunoResearch Labs Cat# 715-165-150, RRID: AB_2340813, United Kingdom) or for the Iba1 staining goat anti rabbit Cy3 (1:200, Jackson ImmunoResearch Labs, RRID: AB_233800).
Validation	All antibodies used are commercially available, have been previously published and validated extensively both in these previous publications as well as with negative controls (with the same immunohistochemistry protocol without the application of the primary antibody), and when applicable are appropriately cited in the manuscript.

Animals and other research organisms

Policy information about studies involving animals; ARRIVE guidelines recommended for reporting animal research, and Sex and Gender in Research

Laboratory animals	Calcium imaging experiments were performed in younger adult (2-6 months; n=8; 2 females, 6 males) and older adult mice (12-20 months; n=8; 4 females, 4 males; ages chosen for equivalent ranges to the human cohorts 1 and 2, see also Wang et al.93), of a transgenic line expressing a genetically encoded calcium indicator (GCaMP6f; C57BL/6J-Tg (Thy1-GCaMP6f) GP5.5Dkim/J; RRID: IMSR_JAX:024276). Mice were housed in individually ventilated cages (Green line system, Tecniplast) under controlled conditions (22 ±2°C, 55%±10% humidity, 12h light–dark cycle, with lights on at 6 a.m.) with food and water available ad libitum. Histological analysis was performed in 12 of these mice in relation to the expression of PV positive (PV+) neurons and in an additional 26 mice in relation to the expression of PV+ neurons, MBP expression as an indication of myelination, and Iba1 expression as a marker for microglia (younger adult mice [n=11; 6 females, 5 males, 2-6 months], older adult mice [n=7; 4 females, 3 males; 12-20 months], and mice in old age [n=8; 3 females, 5 males; +24 months]). All experiments were performed with reference to the NIH Guide for the Care and Use of Laboratory animals (2011)94, and in accordance with the European Communities Council Directive (2010/63/EU) and approved by local authorities of Sachsen-Anhalt/Germany (42502-2-1479 DZNE).
Wild animals	No wild animals were included.
Reporting on sex	Both female and male mice were included, but sex was not a factor considered in the study design.
Field-collected samples	No field-collected samples were included.
Ethics oversight	All experiments were performed according to the NIH Guide for the Care and Use of Laboratory animals (2011) and the Directive of the European Communities Parliament and Council on the protection of animals used for scientific purposes (2010/63/EU) and were approved by the animal care committee of Sachsen-Anhalt, Germany

Note that full information on the approval of the study protocol must also be provided in the manuscript.

Plants

Seed stocks

Report on the source of all seed stocks or other plant material used. If applicable, state the seed stock centre and catalogue number. If plant specimens were collected from the field, describe the collection location, date and sampling procedures.

Novel plant genotypes

Describe the methods by which all novel plant genotypes were produced. This includes those generated by transgenic approaches, gene editing, chemical/radiation-based mutagenesis and hybridization. For transgenic lines, describe the transformation method, the number of independent lines analyzed and the generation upon which experiments were performed. For gene-edited lines, describe the editor used, the endogenous sequence targeted for editing, the targeting guide RNA sequence (if applicable) and how the editor was applied.

Authentication

Describe any authentication procedures for each seed stock used or novel genotype generated. Describe any experiments used to assess the effect of a mutation and, where applicable, how potential secondary effects (e.g. second site T-DNA insertions, mosiacism, off-target gene editing) were examined.

Magnetic resonance imaging

Experimental design

Design type

Resting-state and blocked-design

Design specifications

For cohort 1, a phase-encoded protocol was applied (2 runs of 20 cycles; each fingertip stimulated 20 times for 5.12 seconds) in forward (thumb to little finger, 50% forward-run first) and reverse order (little finger to thumb, 50% reverse-run first). One run took 8 minutes and 32 seconds (256 scans, TR of 2 seconds). A blocked-design protocol was used to stimulate the fingers in a pseudo-random way (2 runs; 6 conditions: stimulation to thumb, index, middle, ring, little finger and no stimulation). One run took 6 minutes and 56 seconds (each fingertip was stimulated 10 times for 2 seconds followed by a 22 seconds resting phase; inter-stimulus intervals of 2 seconds in 70% of trials or 6 seconds in 30% of trials were counterbalanced between fingers; 208 scans). Resting-state data in a 5-minute scan were collected. Total scan time was approximately 40 minutes.

For the motor paradigm of cohort 1, a blocked-design paradigm where participants carried out motor movements of the left and the right hand, the left and the right foot (investigation not part of this study) and the tongue. Movements were carried out for 12 seconds each followed by a 15 seconds rest period. Movements were repeated four times each resulting in 20 trials in total. The total scanning time was approximately 9 minutes for this run.

For cohort 2, The blocked-design run comprised three conditions. Each finger was stimulated for 8 seconds in a pseudo-random sequence, where one finger was stimulated maximally two times in a row. In 70% of the trials, there was a 4 seconds pause between two subsequent stimulations, in 30% of the trials, there was a 8 seconds pause between two subsequent stimulations. This was counterbalanced across fingers. Each finger was stimulated 20 times. One run comprised 264 scans, and lasted for 8 minutes and 48 seconds. The blocked-design run was repeated twice, lasting around 20 minutes in total. The phase-encoded runs included three different conditions. Each condition comprised four runs, each consisting of eight stimulation cycles and two rest conditions of 32 seconds (one before and one after stimulation). Each stimulation cycle lasted 32 seconds, and stimulation was applied to each section of the phalanx four times for 8 seconds. Half of the stimulation runs of each condition were delivered in a forward order (top→down) and the other half in a reverse order (down→top). Half of the participants of each age group started with the forward-run, the other half started with the reversed-run. One run comprised 160 scans (128 scans for stimulation and 32 scans for rest), lasting 320 seconds for a TR of 2 seconds. All phase-encoded runs took around 60 minutes.

Behavioral performance measures

For cohort 1:
Tactile detection task: mean stimulus intensity across reversal points (change of response from correct to incorrect or incorrect to correct) within the period of stable performance (i.e., the last 10 trials).
Finger discrimination task: d-prime as measure of discrimination sensitivity.
Two-point discrimination task: The two-point discrimination threshold was taken from the pin distance where the 50 percent level crossed a fitted sigmoid curve.
The precision grip task: The time (in seconds) the controllable bar was within a given percentage above (2.5%) and below (2.5%) the target line (upper edge of the reference bar).

For cohort 2:
Tactile detection task: the accuracy of successfully distinguishing tactile stimulation.
Texture roughness test: the accuracy of successfully distinguishing different tactile textures.
Grooved pegboard test: the number of successfully filled holes within fixed time
The O'Conner finger dexterity test: the time needed to fill all the holes
Small motor test: the number of successfully paired elements within fixed time

The data were checked for outliers using mean and three times the standard deviation.

Acquisition

Imaging type(s): Functional and structural

Field strength: 7T and 3T

Sequence & imaging parameters:

For functional 7T: GRE EPI pulse sequences (cohort 1, sessions 4 and 5: 1 mm isotropic resolution, FoV read: 192 mm, TR=2000 ms, TE=22 ms, GRAPPA 4, interleaved acquisition, 36 slices; cohort 2, session 8 and 9: 0.9 mm isotropic resolution, 30 slices, interleaved acquisition, FoV read=216 mm, TR=2000 ms, TE=22 ms, GRAPPA 4).

For structural 7T: MP2RAGE images with whole brain coverage for both cohorts (0.7 mm isotropic resolution, 240 sagittal slices, FoV read=224 mm, TR=4800 ms, TE=2.01 ms, inversion time TI1/TI2=900/2750 ms, flip angle (α)=5°/3°, bandwidth=250 Hz/Px, GRAPPA 2).

In addition for cohort 1: MP2RAGE images with part brain coverage (targeting the sensorimotor cortex; 0.5 mm isotropic resolution, 208 transversal slices, FoV read=224 mm, TR=4800 ms, TE=2.62 ms, inversion time TI1/TI2=900/2750 ms, flip angle (α)=5°/3°, bandwidth=250 Hz/Px, GRAPPA 2, phase oversampling=0%, slice oversampling=7.7%), and susceptibility-weighted images with part brain coverage (targeting the sensorimotor cortex) using a 3D gradient-recalled echo (GRE) pulse sequence (0.5 mm isotropic resolution, 208 transversal slices, FoV read=192 mm, TR=22 ms, TE=9.00 ms, flip angle =10°, bandwidth=160 Hz/Px, GRAPPA 2, phase oversampling=0%, slice oversampling=7.7%).

For structural 3T: standard structural 3D MPRAGE, resolution: 1.0 mm, 192 slices, FoV read=192 mm×256 mm, slab thickness=256 mm, TI=650 ms, echo spacing=6.6 ms, TE=4.73 ms, flip angle=8°, bandwidth=191 Hz/Px

Area of acquisition:

For 7T functional: part brain coverage (targeting the sensorimotor cortex)
For 7T structural: whole brain and part brain coverage (targeting the sensorimotor cortex)
For 3T structural: whole brain

Diffusion MRI: ☐ Used ☒ Not used

Preprocessing

Preprocessing software:

For 7T structural: CBS Tools (v3.0.8) as a plugin for MIPAV (v7.3.0) for registration, segmentation and layering, ITK-SNAP 3.8.0 and ANTs for registration, QSM box 2.0 for reconstruction of QSM images
For 7T functional: MATLAB (SPM8 and SPM12) and LIPSIA 3.1.0
For 3T structural: Csurf recon-all for segmentation

Normalization: No data was normalized.

Normalization template: No normalization template was used.

Noise and artifact removal:

Opposite polarity (PE) EPIs were distortion-corrected using point spread function (PSF) mapping.
For resting state data: to prepare the physiological data for noise correction and to remove acquisition artifacts, the open-source Python-based software 'PhysioNoise' was used. Resulting respiratory and cardiac phase data were used to correct the resting-state time series for pulse- and respiration-induced noise by performing RETROspective Image CORrection (RETROICOR) on a slice-by-slice basis.

Volume censoring: No volume censoring was performed.

Statistical modeling & inference

Model type and settings: For 7T functional: GLM (1st level analysis), Fourier Transformation and population receptive field modeling

Effect(s) tested: Permutation mixed-effects ANOVA, independent-samples random permutation Welch t-tests, linear random intercept model and Bayesian independent-sample t tests

Specify type of analysis: ☐ Whole brain ☒ ROI-based ☐ Both

Anatomical location(s):

For cohort 1, area 3b was manually delineated based on an operational definition using anatomical landmarks extracted from cytoarchitectonic, fMRI and multimodal parcellation studies, i.e., following a standardized procedure that has been used previously. All masks were plotted in reference to co-registered Freesurfer labels (normalized probabilistic maps of area 3a and area 3b) on the individual cortical surfaces to ensure that the locations of the manual delineations overlap with those outlined by automated approaches. Additional functional localizers were used to locate specific body parts in area 3b.

For cohort 2, area 3b and the hand area were defined for each individual based on the atlas provided in csurf. Additional functional localizers were used to locate specific body parts in area 3b.

Statistic type for inference: voxel-wise

(See Eklund et al. 2016)

Correction	FDR for GLM, Fourier Transformation and population receptive field modeling<br>Bonferroni methods for multiple comparison

Models & analysis

n/a	Involved in the study
☒	☐ Functional and/or effective connectivity
☒	☐ Graph analysis
☒	☐ Multivariate modeling or predictive analysis
